# Global, regional, and national prevalence of pterygium in 2023: a systematic review and modelling analysis

**DOI:** 10.7189/jogh.16.04271

**Published:** 2026-07-17

**Authors:** Shiyi Shan, Jing Wu, Xinyu Liu, Jiali Zhou, Yutong Zheng, Igor Rudan, Peige Song

**Affiliations:** 1 Center of Clinical Big Data and Analytics of the Second Affiliated Hospital and School of Public Health, Zhejiang University School of Medicine, Hangzhou, China; 2 School of Public Health, Zhejiang University School of Medicine, Hangzhou, Zhejiang, China; 3 Department of Epidemiology and Health Statistics, Sichuan University West China School of Public Health, Sichuan, China; 4 Centre for Global Health, Usher Institute, University of Edinburgh, Edinburgh, UK; 5 Nuffield Department of Primary Care Health Sciences, Oxford University, Oxford, UK

**Keywords:** pterygium, global, prevalence, modelling analysis

## Abstract

**Background:**

Pterygium contributes to otherwise avoidable vision loss and reduced quality of life worldwide. Despite its treatability and the availability of low-cost interventions, it is often overlooked in global health strategies. We aimed to estimate global, regional, and national pterygium prevalence and examine variations in prevalence by age and sex.

**Methods:**

We systematically searched PubMed, Embase, and MEDLINE for articles reporting on the prevalence of pterygium published from 1 January 1990 to 25 September 2024. For China-specific data, we supplemented this search with articles included in a prior systematic review and meta-analysis, and an updated search of four Chinese databases (CNKI, Wanfang, CBM, and VIP) from 1 January 2016 to 25 September 2024. We developed multilevel multivariable mixed-effects meta-regression models to estimate the global, regional, and national age- and sex-specific prevalences of pterygium in 2023, and an associated factor-based model to adjust national prevalence estimates.

**Results:**

We included 103 articles for analysis. The global prevalence of pterygium was 8.22% (95% confidence interval (CI) = 5.31–12.43) in 2023, amounting to 552.85 million (95% CI = 356.93–835.74) cases. We observed that the prevalence increased with age, and that it did not differ substantially by sex, *i.e.* among men (8.36%; 95% CI = 5.40–12.63) and women (8.08%; 95% CI = 5.21–12.22). We found the highest regional prevalence of pterygium in low- and middle-income countries of the South-East Asia Region (13.52%; 95% CI = 8.98–19.65) in 2023, and the highest national prevalence in Saint Helena (22.57%; 95% CI = 11.79–39.47).

**Conclusions:**

Our analysis indicates that pterygium represents a substantial global burden that can progress to severe visual impairment if untreated. Targeted public health strategies are urgently needed to prevent pterygium-related vision loss worldwide.

**Registration:**

PROSPERO: CRD420251056115

Pterygium is a common ocular surface disorder characterised by a wing-shaped fibrovascular proliferation of conjunctival tissue extending onto the cornea [1]. Histopathologically, it is associated with elastotic degeneration, stromal fibrosis, and chronic inflammation [1-3]. Although often considered benign, pterygium can cause ocular discomfort, photophobia, and cosmetic concerns [4]. In many settings, particularly where access to timely surgical care is limited, the condition contributes to avoidable vision loss and reduced quality of life [2,3].

Pterygium is strongly associated with cumulative exposure to ultraviolet (UV) radiation, with its prevalence consistently higher in populations residing in lower latitudes [5]. Other established risk factors include increasing age, male sex, rural residence, outdoor occupations, and socioeconomic disadvantage [6,7]. These exposures are highly concentrated in low- and middle-income countries (LMICs), where healthcare infrastructure is often limited. Previous meta-analyses have estimated the global prevalence of pterygium to be around 10–12% [8,9], but these estimates mask substantial variation across geographies and population groups. For example, the prevalence in China has been reported at 9.8% among adults aged 15–84 years [10], and as high as 38.7% among adults aged 20 years and older in Gondar City, Ethiopia [11]. Despite this growing body of epidemiological evidence, the condition remains largely absent from national eye health plans and global health strategies, despite the availability of feasible, affordable prevention through UV-protective behaviours and treatment *via* surgical excision [2,12].

The World Health Organization (WHO) World Report on Vision [13] and the Lancet Global Health Commission on Global Eye Health [14] have called for robust epidemiological data to inform equitable progress toward universal health coverage. However, the lack of standardised and up-to-date global prevalence estimates limits the ability of policymakers and clinicians to identify high-risk populations and allocate resources effectively. To address this gap, we conducted a systematic review and modelling analysis of population-based studies reporting the prevalence of pterygium in the general population. Using these data, we aimed to calculate global, regional, and national estimates of the prevalence of pterygium, and to quantify its associated factors and examine its variations by age and sex.

## METHODS

We registered this systematic review and meta-analysis in PROSPERO (CRD420251056115) and reported its findings per the PRISMA and the GATHER statements [15,16].

### Systematic review

We searched PubMed, Embase, and MEDLINE for articles reporting on the prevalence of pterygium published from 1 January 1990 to 25 September 2024. For China-specific data, we supplemented this with research included in a previously published systematic review and meta-analysis [10] and an updated search of four Chinese databases (CNKI, CBM, Wanfang, and VIP) for articles published between 1 January 2016 and 25 September 2024. The search strategy combined MeSH terms and free-text keywords relating to ‘pterygium’ and ‘prevalence’ (Appendix S1 in the **Online Supplementary Document**).

After deduplication, two researchers (JW and YZ) independently screened titles and abstracts of all retrieved records, followed by the full texts of any that remained afterwards. We considered population-based studies that reported numerical prevalence estimates for pterygium, with clear assessment and diagnostic methods. Specifically, the presence of pterygium was defined as a wing-shaped extension of the conjunctiva onto the clear cornea [17]. We excluded studies based on clinical or hospital-based samples, case reports, reviews, conference abstracts, commentaries, and studies of non-representative populations (*e.g.* diabetic population). Where multiple publications reported on the same study population, we retained the most recent or most comprehensive article with the largest sample size. We also screened the reference lists of included articles and relevant reviews [8,9] to identify additional eligible studies.

#### Data extraction and quality assessment

Using a standardised data collection template, two researchers (JW and YZ) extracted the following data from all included articles, with a subsequent check by a third researcher (SS):

− bibliographic information: title, authors, publication year;

− study-level characteristics: study location, study year, study setting (urban, rural, or mixed), inclusion and exclusion criteria, sample size, definition and examination of pterygium;

− participant demographics: age range, mean or median age, sex, proportion of females;

− prevalence data: sample size and the number of people with pterygium, stratified by age and sex (where available).

Where the study year was not reported, we imputed it as four years prior to the publication year, based on the average lag between investigation and publication observed across included studies (Appendix S3 and Table S1 in the **Online Supplementary Document**). When age groups were censored (*e.g.* ≥80 years) and when no age statistics were reported, we assumed the same age-band width as other age groups within the same study; if mean or median age was available, we defined the terminal age-band by centring it around the reported statistic. If sex distribution was not provided, we assumed an equal distribution (50% female).

We classified each study by its country or territory of investigation according to the corresponding World Bank (WB) region (high income countries (HICs) and LMICs) and WHO region (African Region (AFR), Region of the Americas (AMR), Eastern Mediterranean Region (EMR), European Region (EUR), South-East Asia Region (SEAR), and Western Pacific Region (WPR)). We then combined these two classifications to form ten distinct WB-WHO regions: HICs-AMR, HICs-EMR, HICs-EUR, HICs-WPR, LMICs-AFR, LMICs-AMR, LMICs-EMR, LMICs-EUR, LMICs-SEAR, and LMICs-WPR (Appendix S3 and Table S2 in the **Online Supplementary Document**). The longitude and latitude of study location were obtained using Google Maps. A subset of studies additionally investigated potential associated factors of pterygium. We included only those reporting adjusted odds ratios (ORs) and their uncertainty estimates from multivariable analyses, provided they used consistent definitions for the associated factors.

We assessed the study quality using a standardised appraisal tool for prevalence data, based on the Joanna Briggs Institute Critical Appraisal Checklist for Prevalence Studies and previous systematic reviews [18,19]. This tool contains nine relevant questions scored from zero (no) to one point (yes), with a total score ranging from zero to nine (Appendix S3 and Table S3 in the **Online Supplementary Document**).

Disagreements during screening, data extraction, and quality assessment were resolved by consensus through discussion or by consultations with a senior researcher (PS).

### Statistical analysis

**Figure 1:**
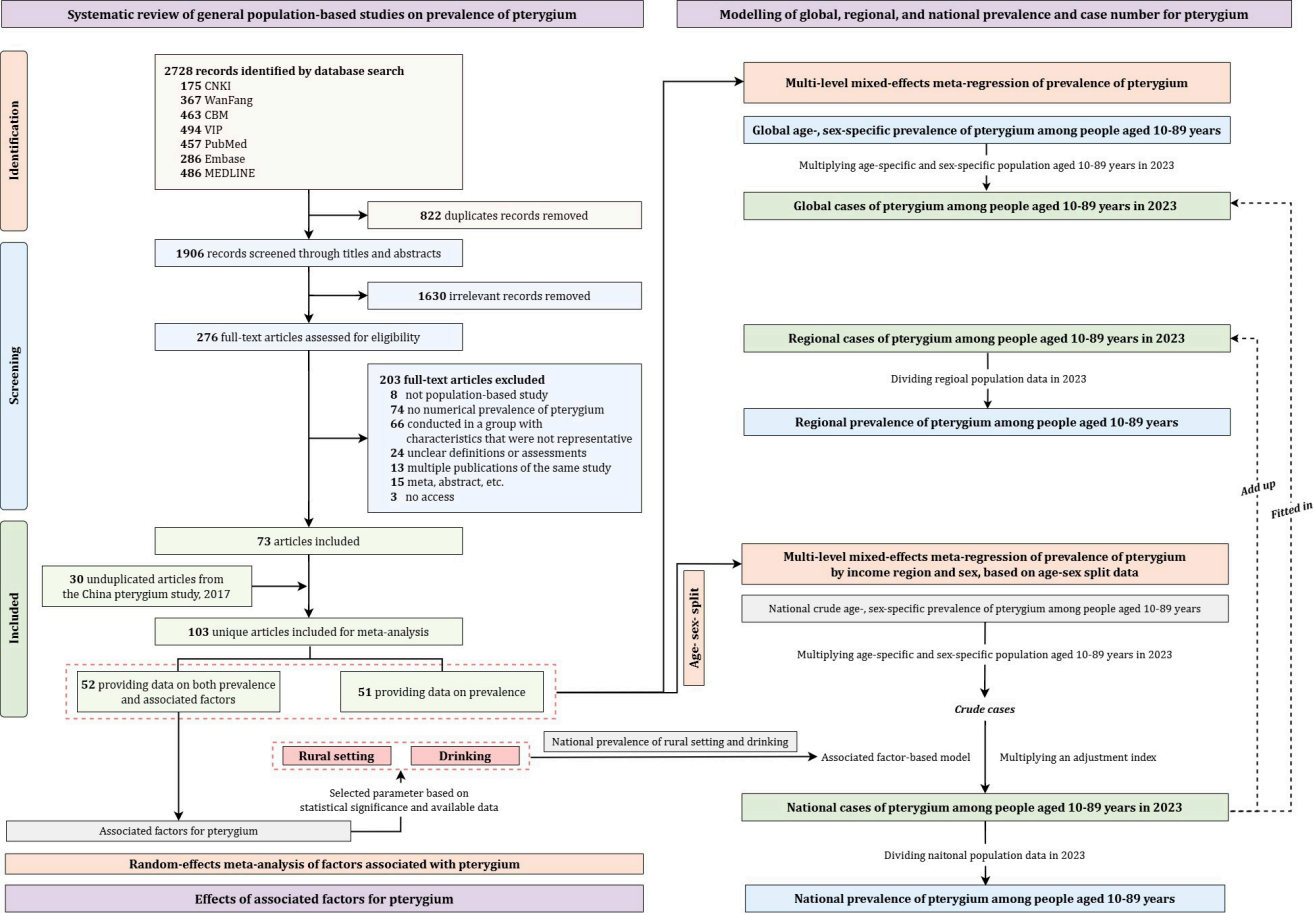
Flow diagram and analytical framework.

We first developed a global multilevel mixed-effects model to establish a global age- and sex-specific prevalence envelope. Then, we estimatedthe national prevalence using meta-regression models incorporating geographic latitude, followed by a further adjustment based on significant associated factors (rural setting and drinking). Finally, regional estimates were generated by aggregating these national-level data (**Figure 1**; Appendix S2 in the **Online Supplementary Document**). 

#### Epidemiological modelling of global prevalence for pterygium

We developed a multilevel multivariable mixed-effects meta-regression model to estimate the global age- and sex-specific prevalence of pterygium. Average age, female proportion, along with their interaction term were then incorporated as fixed effects. Since the study year was not statistically significant after adjusting for average age and the proportion of females, we did not include it in the global prevalence model (Appendix S3 and Table S4 in the **Online Supplementary Document**). We controlled for clustering of multiple datapoints from the same country by adding country identification as a random effect. We performed restricted cubic regression splines to model the functional forms of the nonlinear association of age and pterygium prevalence, with knots being selected by visual inspection at the inflection points of the curve. For studies reporting zero cases, we substituted a small nonzero value (0.0005) to allow for logit transformation. Given that most data points were concentrated within this range, we restricted estimates to 10–89 years. We specified the model as follows:



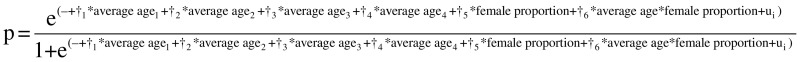



Here, *p* denotes prevalence, *α* is the intercept term, *average age*_1_ − *average age*_4_  are variables generated in the process of fitting cubic splines, *β* is the coefficient, and *u_i_* represents the random effects. The knots of restricted cubic spline were 24.5, 45.5, 57.7, 69.5, and 84.5.

We then generated the global age- and sex-specific prevalence of pterygium based on the above models (Appendix S3, Table S5, and Figure S1 in the **Online Supplementary Document**). Sensitivity analyses using alternative substitution values (0.0001 and 0.001) confirmed that the model coefficients were robust (Appendix S3 and Table S6 in the **Online Supplementary Document**). We then generated the global case number of pterygium aged 10–89 years in 2023 (global envelope) by multiplying the estimated age- and sex-specific prevalence of pterygium with the corresponding population data, obtained from the most recent data of United Nations Population Division (UNPD)[20].

We employed an age-sex-splitting procedure to improve the availability of data for further prevalence modelling. The global age- and sex-specific pterygium prevalence was further used as an age and sex pattern to disaggregate combined-sex data into sex-specific estimates. Likewise, broad age categories were split into one-year age intervals using the fitted age-specific pattern (Appendix S2 in the **Online Supplementary Document**).

#### Epidemiological modelling of regional and national prevalence for pterygium

First, based on age-sex-split pterygium prevalence data, we established four multilevel mixed-effects meta-regression models for males and females in HICs and LMICs, respectively. Considering the potential influence of geographical factors on pterygium prevalence and the correlations among different geographical factors, we categorised the absolute value of latitude into four latitude categories (0–19.9, 20–29.9, 30–34.9, and ≥35) and included it in the national prevalence estimation model. We incorporated the age, sex, and the latitude category of the study location for each individual data point as a fixed-effect variable, with country identification as the random-effect, respectively. We calculated the mean centre point of the country or territory and used the corresponding latitude of the centre point to predict the prevalence of pterygium of each country or territory.



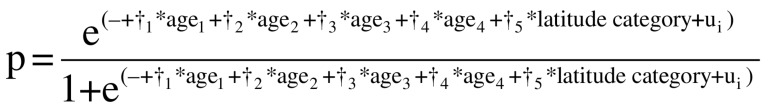



We finally generated the crude national age- and sex- specific prevalence of pterygium based on the above models (Appendix S3 and Table S7 in the **Online Supplementary Document**). We then generated crude national case numbers of pterygium among individuals aged 10–89 years in 2023 by multiplying the estimated age- and sex-specific prevalence of pterygium in 2023 with the corresponding UNPD population data [20].

Second, we used a random-effects (restricted maximum likelihood) meta-analysis to synthesise the effects of associated factors with at least three informative datapoints, which allowed us to explore the effects of 16 associated factors with shared similar definitions (Appendix 3 and Table S8 in the **Online Supplementary Document**). We also evaluated the credibility of each result as convincing (class I), highly suggestive (class II), suggestive (class III), weak (class IV), or no evidence (class NS) and the quality of evidence for each result as ‘high’, ‘moderate’, ‘low’, or ‘very low’ according to the GRADE framework (Appendix 3 and Tables S9 and S10 in the **Online Supplementary Document**) [21,22].

Third, we incorporated two associated factors (rural setting and drinking) into the following associated factor-based model, because of their statistically significant effects on pterygium prevalence and available national prevalence data obtained from UNPD [23] and WHO [24], respectively. The crude national case number of pterygium aged 10–89 years in 2023 was then adjusted through this associated factor-based model.



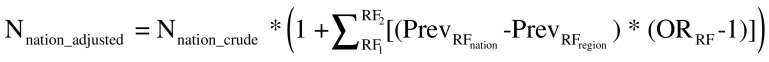



Here, *N_nation_adjusted_* and *N_nation_crude_* are the adjusted and crude numbers of pterygium cases among people aged 10–89 years in each country and territory. *RF*_1_ − *RF*_2_ are the two selected associated factors, *i.e.* rural setting and drinking. *Prev_RFnation_* and *Prev_RFregion_* are the prevalence of the two associated factors in each country and territory and the ten WB-WHO regions. *OR_RF_* is the synthesised OR of rural setting and drinking from the previous meta-analysis.

For each country and territory, we additionally used an adjustment index used to ensure that the sum of national cases fitted within the global envelope. Then, we calculated the adjusted national prevalences of pterygium by the number of pterygium cases in each country and territory divided by its corresponding population.

Finally, we calculated the regional cases for pterygium by aggregating the national cases within each WB-WHO region, and the regional prevalence by dividing the number of cases in each WB-WHO region by its corresponding population estimates from UNPD.

We conducted all statistical analyses in *R*, version 4.4.2 (R Core Team, Vienna, Austria), and generated maps in ArcMap, version 10.8 (Environmental Systems Research Institute, Redlands, California, USA). All tests were two-sided, and *P*-values <0.05 or 95% confidence interval (CIs) excluding the null value were considered statistically significant.

## RESULTS

### Systematic review and study characteristics

**Figure 2:**
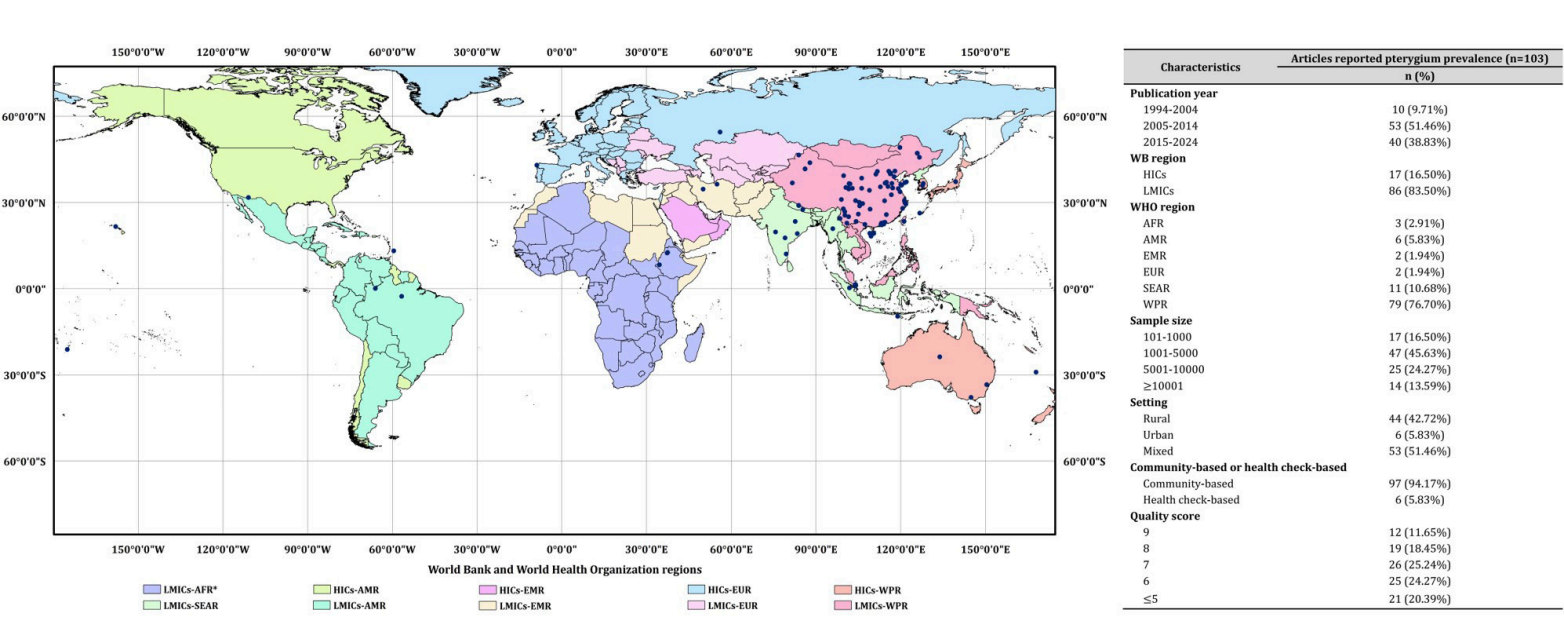
Location and main characteristics of included studies. AFR – African Region, AMR – Region of the Americas, EMR – Eastern Mediterranean Region, EUR – European Region, HIC – high-income country, LMIC – low- and middle-income country, SEAR – South-East Asia Region, WB – World Bank, WHO – World Health Organization, WPR – Western Pacific Region.

The initial search retrieved 2,728 records (**Figure 1**); after deduplication, 1,906 records underwent title and abstract screening, with 1,630 being excluded and the remaining 276 retained for full-text review. Finally, 73 articles met the inclusion criteria, which were supplemented by an additional 30 from the previous review [10] after deduplication, leaving 103 studies comprising 11,720,289 participants for analysis (Appendix S4 in the **Online Supplementary Document**). Regarding study characteristics (**Figure 2**; Appendix 3 and Tables S11 and S12 in the **Online Supplementary Document**), about two-fifths (n = 40, 38.83%) were published after 2015 and onward; most (n = 86, 83.50%) were conducted in LMICs and originated from WPR (n = 79, 76.70%); and most (n = 97, 94.17%) were based on community-dwelling populations and achieved quality scores of six or higher (n = 82, 79.61%).

### Global prevalence and case number of pterygium in 2023

**Figure 3:**
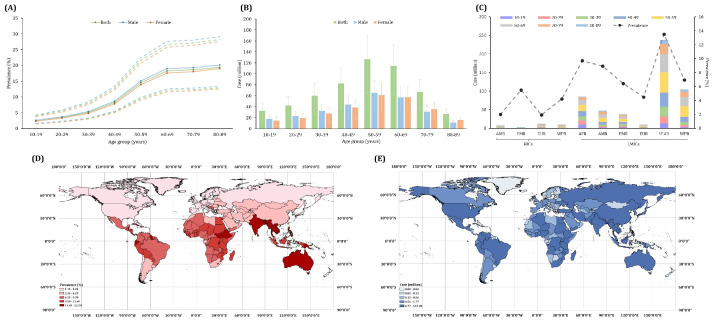
Global, regional, and national prevalence and case number of pterygium among individuals aged 10–89 years. **Panel A.** Global sex-specific prevalence of pterygium by age group. **Panel B.** Global sex-specific case number of pterygium by age group. **Panel C.** Regional prevalence and case number. **Panel D.** National prevalence. **Panel E.** National case number. AFR – African Region, AMR – Region of the Americas, EMR – Eastern Mediterranean Region, EUR – European Region, HICs – high-income countries, LMICs – low- and middle-income countries, SEAR – South-East Asia Region, WPR – Western Pacific Region.

We estimated the global prevalence of pterygium in 2023 to be 8.22% (95% CI = 5.31–12.43), amounting to 552.85 million (95% CI = 356.93–835.74) cases worldwide (**Figure 3**, **Table 1**). We observed a progressive increase in prevalence with advancing age, rising from 2.46% (95% CI = 1.50–3.99) among 10–19-year-olds to 19.43% (95% CI = 12.88–28.21) among those aged 80–89 years, with a slowing in this trend after the age of 60 years. The global prevalence among men was 8.36% (95% CI = 5.40–12.63), compared to 8.08% (95% CI = 5.21–12.22) among women. Regarding case numbers, individuals age 50–59 years accounted for the largest estimated case count at 126.69 million (95% CI = 82.60–188.63). Men had a slightly higher total case number (282.07 million; 95% CI = 182.23–426.03) than women (270.79 million; 95% CI = 174.70–409.72). This pattern reversed in older age groups; for instance, we found higher case numbers (15.69 million; 95% CI = 10.39–22.85) among women aged 80–89 years compared to men (10.88 million; 95% CI = 7.24–15.75).

**Table 1:** Global prevalence and case number of pterygium among individuals aged 10–89 years

	Prevalence, % (95% CI)			Case number in millions (95% CI)		
**Age in years**	**Both**	**Male**	**Female**	**Both**	**Male**	**Female**
10–19	2.46 (1.50–3.99)	2.61 (1.60–4.25)	2.29 (1.40–3.73)	32.54 (19.88–52.92)	17.86 (10.92–29.02)	14.68 (8.96–23.90)
20–29	3.50 (2.17–5.60)	3.71 (2.30–5.92)	3.28 (2.03–5.26)	42.53 (26.36–68.05)	23.19 (14.38–37.05)	19.35 (11.98–31.00)
30–39	5.08 (3.19–8.02)	5.36 (3.36–8.44)	4.80 (3.00–7.58)	60.50 (37.91–95.43)	32.63 (20.47–51.38)	27.88 (17.45–44.05)
40–49	8.27 (5.26–12.78)	8.67 (5.52–13.37)	7.86 (4.99–12.17)	82.56 (52.48–127.51)	43.75 (27.85–67.41)	38.81 (24.63–60.09)
50–59	14.47 (9.44–21.55)	15.09 (9.86–22.39)	13.87 (9.02–20.73)	126.69 (82.60–188.63)	65.53 (42.82–97.23)	61.16 (39.78–91.40)
60–69	18.29 (12.1–26.68)	19.00 (12.60–27.62)	17.63 (11.63–25.82)	114.39 (75.66–166.90)	56.90 (37.75–82.71)	57.49 (37.92–84.20)
70–79	18.62 (12.33–27.13)	19.31 (12.82–28.03)	18.05 (11.92–26.39)	67.06 (44.40–97.70)	31.33 (20.80–45.48)	35.73 (23.60–52.22)
80–89	19.43 (12.88–28.21)	20.12 (13.38–29.12)	18.97 (12.56–27.63)	26.58 (17.63–38.61)	10.88 (7.24–15.75)	15.69 (10.39–22.85)
10–89	8.22 (5.31–12.43)	8.36 (5.40–12.63)	8.08 (5.21–12.22)	552.85 (356.93–835.74)	282.07 (182.23–426.03)	270.79 (174.70–409.72)

CI – confidence interval

### Factors associated with pterygium

Twelve of 16 examined factors showed significant associations with pterygium (Appendix 3, Figure S2, and Table S8 in the **Online Supplementary Document**). Each additional year of age was associated with higher odds of pterygium, with a meta-OR of 1.03 (95% CI = 1.02–1.03) per year increase. Men were at 26% higher risk (meta-OR = 1.26; 95% CI = 1.04–1.51) compared to women. Individuals residing in rural settings had a higher risk of having pterygium than those living in urban settings, with a meta-OR of 2.20 (95% CI = 1.52–3.18). Drinking was also associated with higher odds of having pterygium (meta-OR = 1.31; 95% CI = 1.07–1.59), as were dry eye symptoms (meta-OR = 1.37; 95% CI = 1.10–1.71), high-density lipoprotein cholesterol (meta-OR = 1.23 per 1 mmol/L; 95% CI = 1.08–1.40), and weight (meta-OR = 1.01 per 1 kg; 95% CI = 1.00–1.01). While having an outdoor job was positively associated with a higher prevalence of pterygium (meta-OR = 1.66; 95% CI = 1.38–1.99), using spectacles (meta-OR = 0.73; 95% CI = 0.62–0.87) and sunshade products (meta-OR = 0.47; 95% CI = 0.29–0.74) showed converse associations.

### Regional and national prevalence and case number of pterygium in 2023

We observed marked geographical and socioeconomic variations in pterygium prevalence across the ten WB-WHO regions and 237 countries or territories (**Figure 3**; Appendix 3, Figure S3, and Tables S13 and S14 in the **Online Supplementary Document**). Specifically, we found the highest regional prevalence in LMICs-SEAR (13.52%; 95% CI 8.98–19.65) and the lowest in HICs-EUR (1.92%; 95% CI 0.91–4.00). Similarly, LMICs-SEAR accounted for the largest share of global cases in 2023 (237.34 million; 95% CI = 157.64–344.88). At the national level, Saint Helena (British overseas territory) had the highest prevalence of pterygium (22.57%; 95% CI = 11.79–39.47), while India had the largest number of cases (167.00 million; 95% CI = 110.78–243.08); Gibraltar (British overseas territory) showed the lowest prevalence (1.15%; 95% CI = 0.55–2.41), while the Holy See had the fewest cases (0.01 thousand; 95% CI = 0.00–0.02).

## DISCUSSION

To our knowledge, this study provides the most comprehensive and up-to-date estimates of pterygium prevalence at the global, regional, and national levels. Based on data from 103 population-based studies involving over 11.7 million individuals, we estimated that 8.22% of people aged 10–89 years were affected by pterygium worldwide in 2023, corresponding to 552.85 million individuals. This prevalence increased progressively with advancing age, from 2.46% among those aged 10–19 years to 19.43% in those aged 80–89 years, although this trend slowed beyond the age of 60 years. We otherwise found no major differences in the overall prevalence or case numbers between sexes, but did identify several factors significantly associated with pterygium, including advanced age, male sex, rural residence, education, alcohol consumption, dry eye symptoms, elevated high-density lipoprotein cholesterol, weight, outdoor occupation, spectacle use, and sunshade product use. Regionally, the estimated prevalence ranged from 1.92% in HICs-EUR to 13.52% in LMICs-SEAR, with the latter also accounting for the highest number of cases globally. At the national level, India had the largest number of cases (167.00 million), while Saint Helena had the highest prevalence (22.57%).

Our global prevalence estimate is somewhat lower than that of previous meta-analyses, which reported rates of 10–12% [9,25]. This is likely due to the broader age range included in our model, which incorporated younger individuals typically at lower risk of pterygium. Previous analyses have further focused on adults or elderly cohorts and thus may have overestimated the population-level prevalence. Methodological differences may also contribute to the discrepancy; our use of multilevel meta-regression allowed partial adjustment for demographic structure and study-level heterogeneity, improving comparability across settings.

We observed a clear age-related increase in the prevalence of pterygium, consistent with the cumulative effect of UV exposure, a well-established risk factor for the condition [26–29]. However, the slowing in the rate after the age of 60 years may reflect changes in occupational exposure, retirement-related behavioural shifts, or survivor bias. Sex differences in overall prevalence were minimal (8.36% among men *vs*. 8.08% among women), as were absolute case numbers (282.07 million *vs*. 270.79 million, respectively) in 2023, suggesting broadly similar lifetime risk. Notably, we identified a reversal in case number in the oldest age groups, with more cases among women aged 80–89 than among men (15.69 million *vs*. 10.88 million, respectively). This finding likely reflects demographic patterns, particularly a longer life expectancy for women in many settings, resulting in a larger population of at-risk older women [30,31]. This underscores the importance of adopting a life-course perspective in pterygium research and prevention, taking into account not only biological factors but also differences in exposure, behaviour, and survival.

We observed a clear inverse association between latitude and the prevalence of pterygium, with significantly higher rates below 20° latitude than above 35°. This latitudinal gradient is consistent with ecological studies linking UV exposure to pterygium pathogenesis [6,32,33]. Research has shown that UV radiation induces oxidative stress and chronic inflammation in the bulbar conjunctiva, leading to fibroblast proliferation and angiogenesis characteristic of the disease [5,34,35]. Notably, we found the highest prevalence in the 20–29.9° latitude band, exceeding that of lower latitudes. Similar findings have been reported elsewhere and may reflect the influence of altitude, cloud cover, and behavioural adaptation [25,36,37]. For example, tropical coastal zones may have high humidity and cloud cover that attenuate UV exposure [38], while high-altitude subtropical regions may receive intense solar radiation [39]. Moreover, protective behaviours, such as wearing hats or limiting outdoor activity, may be more common in low-latitude populations [40–42], while those in subtropical regions may spend more unprotected time outdoors in temperate conditions [43]. These explanations are supported by our meta-analysis of associated factors that rural residence and outdoor occupation were strongly associated with pterygium, while spectacle use and sunshade products were protective factors. These findings reinforce the link between prolonged UV exposure and pterygium development, while supporting the protective role of ocular sun protection measures. Although direct measures of sun exposure did not show significant associations, likely due to limited and heterogeneous data, our findings reinforce the importance of behavioural and occupational context in mediating UV-related risk.

Besides environmental and occupational determinants, several individual-level factors were significantly associated with pterygium. Dry eye symptoms showed a strong positive association, supporting the role of chronic ocular surface inflammation in disease development [44–46]. Alcohol consumption was also associated with increased odds of pterygium, potentially reflecting systemic oxidative stress or impaired ocular surface homeostasis [47]. Metabolic factors, including higher high-density lipoprotein cholesterol and greater body weight, were modestly associated with increased risk [48–50]. Although the biological pathways remain uncertain, these findings may point to shared inflammatory or hormonal mechanisms. These associations suggest that pterygium may reflect not only cumulative UV exposure but also broader systemic and ocular surface conditions. However, as these findings are derived from observational studies, causal relationships cannot be established and residual confounding cannot be excluded.

Our regional analysis indicated geographic and socioeconomic variations in the prevalence of pterygium, with LMICs-SEAR showing the highest prevalence (13.52%) and case number (237.34 million), while HICs-EUR demonstrated the lowest prevalence (1.92%). Low-middle latitude regions like SEAR experience more intense and consistent UV radiation year-round, which is consistent with our finding of an inverse latitude-prevalence relationship [51]. The disproportionate burden in LMICs-SEAR can also be explained by the socioeconomic determinants, where a higher proportion of the population might engage in outdoor jobs with limited access to UV protection measures [52]. Conversely, in HICs-EUR, widespread occupational shifts to indoor work, coupled with greater awareness and availability of UV protection behaviours, may suppress the prevalence of pterygium [53]. Healthcare inequality could also contribute to this disparity, since LMICs often lack structured eye health programmes [14], while HICs integrate eye health or UV protection into public health campaigns, such as the SunSmart programme in Australia [54] and the Healthy Vision Month in the USA [55].

The rigorous eligibility criteria and robust epidemiological model we used to calculate the prevalence and case numbers of pterygium at the global, regional, and national levels enhanced the accuracy of our estimates, while also allowing for the simultaneous adjustment of geographic, demographic, and other variables, providing a more nuanced understanding of disease distribution. Furthermore, due to heterogeneity across different studies, we used meta-regression to account for the discrepancies such as latitude to improve the comparability of different regions worldwide.

However, several limitations must be noted. First, our meta-regression models incorporated aggregated variables at the study level, with country identification as random effects. Individual-level variables such as personal sun-protection behaviours, cumulative occupational UV exposure, genetic susceptibility, and socioeconomic status were not consistently reported across included studies and could not be directly modelled. Although the national prevalence model incorporated latitude category and was stratified by income region to partially capture geographic and socioeconomic variation, and the associated factor-based model further adjusted for rural setting and drinking, these adjustments capture only a limited dimension of individual-level heterogeneity. Residual confounding from unmeasured factors may therefore influence our estimates, and the adjusted national estimates should be interpreted with caution. Second, while we exclude the study year from the global prevalence model for not being a statistically significant factor, we cannot rule out subtle temporal trends that may have been obscured by study heterogeneity. Future longitudinal studies are needed to clarify trends in pterygium prevalence. Third, we employed several imputation steps to maximise data inclusion, including assumptions about study years, age-band widths, and sex distributions. These assumptions were primarily informed by data from well-represented regions and may be less applicable in underrepresented settings. Fourth, the included studies were all observational, limiting causal inference. Fifth, we assigned latitude based on geographic centroids of countries or territories, implicitly assuming homogeneous pterygium prevalence within each area. However, subnational variations may still exist due to differences in altitude, urbanisation, and local climate, potentially biasing our estimates in geographically diverse nations. Sixth, the evidence base included in our model was geographically concentrated, with 76.70% of studies coming from WPR. Although random effects accounted for clustering, estimates for underrepresented regions such as EMR, EUR, and AFR should be interpreted with greater uncertainty. Additionally, variation in assessment methods across settings may have introduced measurement heterogeneity despite a consistent clinical definition.

Given the observed age-dependent increase in the prevalence of pterygium, targeted public health strategies are essential to mitigate disease burden and prevent vision-related complications. Greater disease awareness is critical, particularly among high-risk populations (*e.g.* outdoor workers, rural residents, and older adults), to promote early detection and treatment. Public health interventions such as promoting the use of protective eyewear, shade structures in occupational settings, and community education could meaningfully reduce pterygium incidence and progression, particularly in high-risk populations. Given the simplicity and affordability of surgical treatment, investment in primary-level surgical capacity may also yield high returns in terms of visual function and quality of life. As global UV exposure rises in tandem with climate change and outdoor labour persists in many LMICs, the burden of pterygium is likely to increase further. Demographic ageing may increase the absolute number of cases even if age-specific prevalence remains stable. These dynamics emphasise the need for proactive surveillance and scenario-based modelling to project future burden. Equity-focused strategies will be critical to ensure that effective prevention, early detection, and treatment are accessible to those most at risk.

## CONCLUSIONS

We estimated the global prevalence of pterygium to be 8.22%, corresponding to 552.85 million cases aged 10–89 years worldwide in 2023. While the condition is often perceived as a benign or cosmetic concern, untreated cases can progress to severe visual complications. Our findings also support the link of latitude, age, being male, having an outdoor job, and adhering to UV protection behaviours with pterygium prevalence, highlighting a need for targeted public health strategies to mitigate preventable vision loss globally.

## Additional material


Online Supplementary Document
