# Supplementary material for: Global, regional, and national prevalence of pterygium in 2023: a systematic review and modelling analysis
Source: J Glob Health. 2026 Jul 17;16:04271. doi: 10.7189/jogh.16.04271 (PMC13377759; doi:10.7189/jogh.16.04271)
Supplement: Online Supplementary Document [file jogh-16-04271-s001.pdf]

Supplement to: Shan S, Wu J, Liu X, Zhou J, Zheng Y, Rudan I, Song P; Global Health Epidemiology Research Group (GHERG). Global, regional, and national prevalence of pterygium in 2023: a systematic review and modelling analysis. J Glob Health. 2026;16:04271.

Appendix 1. Search strategy to identify studies reporting the prevalence of pterygium......... 2
Appendix 2. eMethods: The detailed description of stages used to derive global, regional, and national estimations of the prevalence of pterygium. 3
Appendix 3. Supplementary tables and figures.. 7
Table S1. The time-lag between investigation and publication in the included articles. 7
Table S2. Country or territory list in World Bank and World Health Organization regions... 10
Table S3. Quality assessment scale. 11
Table S4. Age- and sex- adjusted meta-regression models of study year category for prevalence of pterygium... 12
Table S5. Multilevel mixed-effects meta-regression models for global prevalence of pterygium. 13
Table S6. Sensitivity analyses for zero-event substitution values in the global prevalence model. 14
Table S7. Multilevel mixed-effects meta-regression models of the national prevalence of pterygium. 15
Table S8. Associated factors of pterygium. 17
Table S9. Evidence credibility grading criteria. 20
Table S10. Evidence quality grading criteria by GRADE.. 21
Table S11. Detailed characteristics of the included articles (n=103). 22
Table S12. Quality scores for the included articles (n=103).. 28
Table S13. Age- and sex-specific prevalence and case number of pterygium by World Bank and World Health Organization region. 34
Table S14. National prevalence and case number of pterygium. 38
Figure S1. Multilevel mixed-effects meta-regression models for prevalence of pterygium. . 48
Figure S2. Summary of associated factors of pterygium. 49
Figure S3. Regional sex-specific prevalence and case number of pterygium...................... 50
Appendix 4. Full list of the included articles (n=103). 51

Appendix 1. Search strategy to identify studies reporting the prevalence of pterygium.

Database	Subject category	Sub-database	Search terms	Publication date	Search method
**CNKI**	Medicine & Public Health	Journal, Featured journal, Doctoral dissertation, Master dissertation	(SU % '翼状胬肉') AND (SU % '发病率' + '发生率' + '患病率' + '罹患率' + '现患'+ '流行' + '现况' + '调查' + '监测' + '新发')	1/1/2016-25/9/2024	Comprehensive search: subject, title, keywords and abstract
**Wanfang**	Not applicable	Journal article, Dissertations	((主题:" 翼状胬肉") AND (主题:"发病率" or 主题: "发生率" or 主题: "患病率" or 主题: "罹患率" or 主题: "现患" or 主题: "流行" or 主题: "现况" or 主题: "调查" or 主题: "监测"or 主题: "新发"))	2016-Now	Comprehensive search: subject (including title, keywords and abstract)
**CBM**	Not applicable	All journals	(("发病率"[不加权:扩展] OR "患病率"[不加权:扩展] OR "发病率"[常用字段:智能] OR "发生率"[常用字段:智能] OR "患病率"[常用字段:智能] OR "罹患率"[常用字段:智能] OR "现患"[常用字段:智能] OR "流行"[常用字段:智能] OR "现况"[常用字段:智能]OR "调查"[常用字段:智能] OR "监测"[常用字段:智能] OR "新发"[常用字段:智能]) AND ("翼状胬肉"[不加权:扩展] OR "翼状胬肉"[常用字段:智能])) AND 2016-2022[日期]	2016-2024	Comprehensive search: all fields
**VIP**	Medicine & Public Health	All journals	(M=(翼状胬肉) OR R=(翼状胬肉)) AND (M=(发病率 OR 发生率 OR 患病率 OR 罹患率 OR 现患 OR 流行 OR 现况 OR 调查 OR 监测 OR 新发) OR R=(发病率 OR 发生率 OR 患病率 OR 罹患率 OR 现患 OR 流行 OR 现况 OR 调查 OR 监测 OR 新发))	2016-2024	Comprehensive search: subject, title, keywords and abstract
**PubMed**	Not applicable	Not applicable	(((((((prevalen*[Title/Abstract]) OR (inciden*[Title/Abstract])) OR (epidemiolog*[Title/Abstract])) OR (morbidit*[Title/Abstract]))) OR (attack rate[Title/Abstract]))) AND ((pterygia[Title/Abstract]) OR (pterygium[Title/Abstract])) AND (("1990/01/01"[Date - Publication] : "3000"[Date - Publication]))	1/1/1990-25/9/2024	Comprehensive search: all fields
**Embase**	Not applicable	Not applicable	#1 'prevalence'/exp OR 'incidence'/exp OR 'epidemiology'/exp OR 'morbidity'/exp OR 'attack rate'/exp OR 'mortality'/exp OR 'prevalen*':ab,ti OR 'inciden*':ab,ti OR 'epidemiolog*':ab,ti OR 'morbidit*':ab,ti OR 'attack rate':ab,ti<br>#2 'pterygium'/exp OR 'pterygium':ab,ti OR 'pterygial*':ab,ti<br>#3 #1 AND #2<br>#4 #3 AND [embase]/lim NOT ([embase]/lim AND [medline]/lim)	01/01/1990-25/9/2024	Comprehensive search: all fields
**MEDLINE**	Not applicable	Not applicable	#1 exp Pterygium / or (pterygium or pterygial*).ab,ti.<br>#2 exp Prevalence/ or exp Incidence/ or exp Epidemiology/ or exp Morbidity/ or (prevalen* or inciden* or epidemiolog* or morbidit* or attack rate).ab,ti.<br>#3 1 and 2<br>#4 limit 3 to yr="1990 -Current"	01/01/1990-25/9/2024	Comprehensive search: all fields

Notes: CNKI, China National Knowledge Infrastructure; CBM, Chinese Biomedicine Literature Database, VIP, Chinese Science and Techn ology Journal Database. Number of records returned was 2,728. The access date was 25th September, 2024.

Appendix 2. eMethods: The detailed description of stages used to derive global, regional, and national estimations of the prevalence of pterygium.

This section is a supplement to the Methods part in the main text.

Stage 1 Epidemiological modelling of global prevalence and case number for pterygium in 2023

A total of 590 data points providing age-specific or sex-specific pterygium prevalence estimates were extracted from 103 included articles. To enable the inclusion of zero cases as reported, zero cells were replaced with a value of 0.0005. To accommodate the hierarchy of multiple articles originating from the same country, we adopted a multilevel mixed-effects meta-regression approach with country identification as the random-effects. Given that,

$$prevalence=p=\frac{Pterygium\ cases}{Number\ of\ participants}$$

Then, to stabilise variance for proportions approaching 0 or 1, the prevalence was stabilized by the logit link,

$$logit(p)=ln\left(\frac{p}{1-p}\right)=ln(odds)=\alpha+\beta_1*x_1+\beta_2*x_2+...+\beta_n*x_n+u_i$$

Therefore,

$$odds=\frac{p}{1-p}=e^{(\alpha+\beta_1*x_1+\beta_2*x_2+...+\beta_n*x_n+u_i)}$$

And,

$$prevalence=p=\frac{e^{(\alpha+\beta_1*x_1+\beta_2*x_2+...+\beta_n*x_n+u_i)}}{1+e^{(\alpha+\beta_1*x_1+\beta_2*x_2+...+\beta_n*x_n+u_i)}}$$

where α is the intercept term, β is the coefficient, x is the variable and u_i is the random-effect.

Age, sex, and study year were our main variables of interest. However, when we performed meta-regression analysis adjusted for age and sex, the study year category did not show statistical significance (**Appendix 3, Table S4**). Thus, study year was not incorporated in the following models and the prevalence of pterygium was regarded as constant throughout the investigation period. Average age, female proportion, and an interaction term of average age and female proportion were included in our global prevalence model to capture the age- and sex-pattern of pterygium prevalence. Restricted cubic regression splines were performed to model the functional forms of the non-linear association of average age and pterygium prevalence, with knots being selected by visual inspection at the inflection points of the curve. The following analysis was restricted to the 10-89-year age range, where sufficient data were available for model development. Therefore, for global model:

$$logit(p)=ln\left(\frac{p}{1-p}\right)=ln(odds)=\alpha+\beta_1*Average\ age_1+\beta_2*Average\ age_2+\beta_3*Average\ age_3+\beta_4*Average\ age_4+$$
$$\beta_5*Female\ proportion+\beta_6*Average\ age*Female\ proportion+u_i$$

Then,

$$prevalence=p=\frac{e^{(\alpha+\beta_1*Average\ age_1+\beta_2*Average\ age_2+\beta_3*Average\ age_3+\beta_4*Average\ age_4+\beta_5*Female\ proportion+\beta_6*Average\ age*Female\ proportion+u_i)}}{1+e^{(\alpha+\beta_1*Average\ age_1+\beta_2*Average\ age_2+\beta_3*Average\ age_3+\beta_4*Average\ age_4+\beta_5*Female\ proportion+\beta_6*Average\ age*Female\ proportion+u_i)}}$$

Where $Average\ age_1$-$Average\ age_4$ are variables generated in the process of fitting cubic splines, with knots of 24.5, 45.5, 57.7, 69.5, 84.5. Based on the above models (**Appendix 3, Table S5** and **Figure S1**), the global age- and sex-specific prevalence of pterygium was generated.

The global case number of pterygium aged 10-89 years in 2023 were then generated by multiplying the estimated age- and sex-specific prevalence of pterygium with the corresponding population data, obtained from the United Nations Population Division (UNPD). This total, termed the "global envelope", representing the total number of pterygium cases aged 10-89 years in 2023 and serves as a ceiling to ensure consistency across national estimates.

Stage 2 Age-sex-splitting for pterygium

Stage 2.1 Age- and sex-specific prevalence patterns for pterygium

To enhance data availability for further regional and national prevalence modelling, we employed an age-sex-splitting method on extracted data points. The estimated global age- and sex-specific prevalence of pterygium was taken as the "prevalence patterns" to provide a basis for further age-sex-splitting process.

Stage 2.2 Sex-splitting for pterygium

Based on the "prevalence patterns" for pterygium, we used an age-sex-splitting approach to split data with standard age and sex groups. First, a sex-splitting was performed on data points specified as "both" sex into male- and female-specific data points. The following equation was employed:

$$C_{A,s}=(\sum_{a\in A} R_{a,s}N_{a,s})\cdot P_{A,s}/N_{A,s}\cdot\frac{C_{A,S}}{\sum_{s\in S}((\sum_{a\in A}R_{a,s}N_{a,s})\cdot P_{A,s}/N_{A,s})}$$

In this equation, s is the specific sex (male or female), S is the set of sexes data is aggregated across, a is a one-year age group, A is the set of ages the data is aggregated across, $C_{A,S}$ is the reported total case number to be split, $R_{a,s}$ is the prevalence in age group a and sex s from "prevalence patterns", $N_{a,s}$ is the population in age group a and sex s based on the 2023 population data from UNPD, $P_{A,s}$ is the proportion of sex s in study sample, $N_{A,s}$ is the population in age group A and sex s based on the 2023 population data from UNPD, and $C_{A,s}$ is the split case number of pterygium in sex s.

Stage 2.3 Age-splitting for pterygium

Subsequently, data points with inconsistent age groups were split into uniform one-year age groups. Given the lack of precise age-specific distribution data in the included studies, we employed an exponential adjustment method with numerical optimisation to simulate the age distributions that align with both the national age distribution and the sample's average age.

We introduced an adjustment parameter adj to modify the national age distribution using an exponential function:

$$\text{Adjusted } P_a=\frac{P_a\cdot e^{adj(a-\mu)}}{\sum_{a\in A}P_a\cdot e^{adj(a-\mu)}}$$

where a is a one-year age group, A is the set of ages the data is aggregated across, adj is the adjustment parameter, μ is the average age of reported sample, P_a is the proportion of age a in the 2023 population data from UNPD, and $\text{Adjusted } P_a$ is the proportion of age a after adjustment. This adjustment increases the proportion of ages above μ if $adj>0$ and increases the proportion of ages below μ if $adj<0$.

The optimal value of adj was estimated using the Brent optimisation method to minimize the squared difference between the average age of the adjusted distribution and the reported average age:

$$\min_{adj}\left(\sum_{a\in A}\text{Adjusted } P_a\cdot a-\mu\right)^2$$

The optimisation was constrained to $adj\in[-10, 10]$ to ensure numerical stability.

After obtaining the optimal adj, we generated the adjusted age distribution $\text{Adjusted } P_a$. We then sampled ages from this distribution to create a simulated population that aligns with both the national age distribution and the target mean age.

An age split was then performed based on the adjusted age distribution:

$$C_a=R_aN_a\frac{C_A}{\sum_{a\in A}R_aN_a}$$

In this equation, a is a one-year age group, A is the set of ages the data is aggregated across, C_A is the reported total case number in ages A to be split, R_a is the prevalence in age group a from "prevalence patterns", N_a is the population in age group a based on the simulated population, and C_a is the split case number of pterygium in age group a.

After performing age-sex-splitting, the case numbers for pterygium from various articles were divided into single-year age groups and separated by sex (male and female).

Stage 3 Epidemiological modelling of national prevalence and case number for pterygium

Stage 3.1 Crude age- and sex-specific prevalence of pterygium at national level

Based on age-sex-split pterygium prevalence data, four multilevel mixed-effects meta-regression models were adopted for males and females in high-income countries (HICs) and low- and middle-income countries (LMICs), respectively. Considering the potential influence of geographical factors on pterygium prevalence and the correlations among different geographical factors, we categorized the absolute value of latitude into four latitude categories (0-19.9, 20-29.9, 30-34.9, ≥35) based on quartiles of the data points' latitude distribution, and incorporated it into the national prevalence estimation model. Thus, to estimate national prevalence of pterygium among people aged 10-89 years, we fitted multilevel multivariable mixed-effects meta-regression models with age, sex, and latitude category as fixed-effect variables, and country identification as the random-effect, respectively.

Therefore,

$$prevalence = p = \frac{e^{(\alpha+\beta_1 * Age_1 + \beta_2 * Age_2 + \beta_3 * Age_3 + \beta_4 * Age_4 + \beta_5 * Latitude\ category + u_i)}}{1+e^{(\alpha+\beta_1 * Age_1 + \beta_2 * Age_2 + \beta_3 * Age_3 + \beta_4 * Age_4 + \beta_5 * Latitude\ category + u_i)}}$$

The mean centre point of the country or territory was calculated and the corresponding latitude of the centre point was used to predict the prevalence of pterygium of each country or territory.

Based on the above models (**Appendix 3, Table S7**), the crude national age- and sex-specific prevalence of pterygium was generated. The crude national case numbers of pterygium aged 10-89 years in 2023 were then generated by multiplying the estimated age- and sex-specific prevalence of pterygium in 2023 with the corresponding population data, obtained from UNPD.

Stage 3.2 Meta-analysis of factors associated with pterygium

A subset of included articles (52 out of 103) additionally investigated associated factors of pterygium using multivariable analysis, and only factors that shared similar definitions were included. Random-effects (Restricted Maximum Likelihood) meta-analyses were conducted to synthesise the effects of associated factors with at least three informative data points, including sociodemographic factors, lifestyle factors, health-related factors, biological factors, anthropometric factors, sun exposure factors, and sun-protective behavior factors. Finally, 16 associated factors were investigated (**Appendix 3, Table S8**):

(1) Age (including 12 sub-factors: per 1 year; 60-69 vs 50-59; 70-79 vs 50-59; ≥80 vs 50-59; ≥80 vs 40-49; 50-59 vs 40-49; 60-69 vs 40-49; 70-79 vs 40-49; 40-49 vs 30-39; 50-59 vs 30-39; 60-69 vs 30-39; ≥70 vs 30-39);

(2) Sex (male vs female);

(3) Setting (rural vs urban);

(4) Education (including four sub-factors: no experience vs any experience; elementary school vs no experience; middle vs no experience; high vs no experience);

(5) Drinking;

(6) Smoking (including four sub-factors: past smoker vs never smoking; current smoker vs never smoking; current smoker vs past smoker and never smoking; current and past smoker vs never smoking);

(7) Hypertension;

(8) Diabetes;

(9) Dry eye symptoms;

(10) High-density lipoprotein cholesterol (HDL-C, per 1 mmol/L);

(11) Weight (per 1 kg);

(12) Height (per 1 cm);

(13) Sun exposure (≥5 hours/day vs <5 hours/day);

(14) Outdoor job;

(15) Spectacles use;

(16) Use of sunshade products.

We also calculated 95% prediction intervals (PIs), extracted the largest study significance, and assessed small study effects and excess significance bias for each associated factor. Based on the above indicators, we evaluated the credibility of each result as convincing ("class I"), highly suggestive ("class II"), suggestive ("class III"), weak ("class IV"), or no evidence ("class NS"). The criteria is shown in **Appendix 3, Table S9**. Besides, the quality of evidence for each result was assessed using the Grading of Recommendations, Assessment, Development, and Evaluation (GRADE) framework, which classifies evidence as "high", "moderate", "low", or "very low" (**Appendix 3, Table S10**). Funnel plots were visually inspected to evaluate publication bias for each factor. Sensitivity analyses were performed using the leave-one-out method.

Stage 3.3 Adjusted age- and sex- specific prevalence of pterygium at national level

The crude national case number of pterygium aged 10-89 years in 2023 was then adjusted through an "associated factor-based model", which was initially proposed by Global Health Epidemiology Reference Group and has been widely used in estimating burden of disease. Two associated factors in ***Stage 3.2***, rural setting and drinking, were selected for the "associated factor-based model", because they were the only factors that simultaneously demonstrated statistically significant associations with pterygium in our meta-analysis and had standardised, nationally comparable prevalence data available from UNPD and World Health Organization (WHO). Then, the national numbers of pterygium cases ("national envelopes") were adjusted by the following formula:

$$N_{nation\_adjusted} = N_{nation\_crude} * \left(1 + \sum_{RF_1}^{RF_2} [(Prev_{RF_{nation}} - Prev_{RF_{region}}) * (OR_{RF} - 1)]\right)$$

Where $N_{nation\_adjusted}$ and N_{nation_crude} are the adjusted and crude numbers of pterygium cases among people aged 10-89 years in each country and territory. RF_1-RF_2 are the two selected associated factors, namely rural setting and drinking. $Prev_{RF_{nation}}$ and $Prev_{RF_{region}}$ are the prevalence of the two associated factors in each country and territory and the ten World Bank (WB)-WHO regions (high income countries [HICs]-Region of the Americas [AMR], HICs-Eastern Mediterranean Region [EMR], HICs-European Region [EUR], HICs-Western Pacific Region [WPR], low- and middle-income countries [LMICs]-African Region [AFR], LMICs-AMR, LMICs-EMR, LMICs-EUR, LMICs-South-East Asia Region [SEAR], and LMICs-WPR). OR_{RF} is the synthesised OR of rural setting and drinking from ***Stage 3.2***.

After adjusting national case numbers using the "associated factor-based model", the sum of national estimates may not precisely equal the "global envelope". To ensure internal consistency, each national estimate was multiplied by an "adjustment index", ensuring that all national cases sum exactly to the global total while preserving the relative distribution across countries. Then, the adjusted national prevalence estimates of pterygium were calculated by the number of pterygium cases in each country and territory divided by its corresponding population. This process established "national envelopes", representing the number of pterygium cases aged 10-89 years in each country and territory in 2023.

Stage 4 Age- and sex-specific prevalence of pterygium at regional level

Finally, we developed "regional envelopes" for pterygium respectively, by summing the cases within each WB-WHO region, and calculated regional prevalence of pterygium through the number of pterygium cases in each WB-WHO region divided by its corresponding population from UNPD.

All analyses were conducted in R version 4.4.2 (https://www.r-project.org).

Appendix 3. Supplementary tables and figures.

Table S1. The time-lag between investigation and publication in the included articles.

ID	Author	Publication year	Study year	Time lag
S01	Dong YP, et al.	2024	2021	3
S02	Adriano L, et al.	2022	2017	5
S03	Zhang XY, et al.	2022	2020	2
S04	Zang S, et al.	2022	2011	11
S05	Tandon R, et al.	2022	2013	9
S06	Padhy D, et al.	2022	NA	NA
S07	Ke HQ, et al.	2022	2019	3
S08	Xiao Li, et al.	2021	2017	4
S09	Hatsusaka N, et al.	2021	2008	13
S10	Zhang Yanling, et al.	2020	2019	1
S11	Liu Yanjun.	2020	2019	1
S12	Wang Y, et al.	2020	2014	6
S13	Pan ZX, et al.	2020	2016	4
S14	Fekadu SA, et al.	2020	2019	1
S15	Alemayehu TK, et al.	2020	2019	1
S16	Fernandes AG, et al.	2019	NA	NA
S17	Wu Xiaolan, et al.	2019	2015	4
S18	Zhang J, et al.	2019	NA	NA
S19	Pan Z, et al.	2019	2017	2
S20	Lin YH, et al.	2019	2006	13
S21	Bikbov MM, et al.	2019	NA	NA
S22	Zhao Lizhen, et al.	2017	2016	1
S23	Yang Chengyi.	2017	2015	2
S24	Su Xiaolong, et al.	2017	NA	NA
S25	Sitompul R, et al.	2017	2016	1
S26	Rim TH, et al.	2017	2009	8
S27	Hashemi H, et al.	2017	2015	2
S28	Cao XG, et al.	2017	2004	13
S29	Anbesse DH, et al.	2017	2016	1
S30	Li Ming, et al.	2016	2009	7
S31	Li Mengqi, et al.	2016	2015	1
S32	Gan Linyang.	2016	2015	1
S33	Bi Yun, et al.	2016	2015	1
S34	Zhong H, et al.	2016	NA	NA
S35	Wang JW, et al.	2016	NA	NA
S36	Lin AD, et al.	2016	2012	4
S37	Chen T,et al.	2015	NA	NA
S38	Ma WZ.	2015	2008	7
S39	Ma F.	2015	2013	2
S40	Cui XH et al.	2015	2010	5
S41	Shrestha S,et al.	2014	2011	3

ID	Author	Publication year	Study year	Time lag
S42	Maharjan IM,et al.	2014	NA	NA
S43	Li Z,et al.	2014	2009	5
S44	Jiao W,et al.	2014	2008	6
S45	Jiang ZY et al.	2014	NA	NA
S46	Li CJ et al.	2014	2013	1
S47	Luo ZL et al.	2014	2013	1
S48	Shen FR.	2014	NA	NA
S49	Tano T,et al.	2013	2009	4
S50	Sun LP,et al.	2013	2007	6
S51	Rim THT,et al.	2013	2009	4
S52	Nangia V,et al.	2013	NA	NA
S53	Marmamula S,et al.	2013	1998	15
S54	Li Z,et al.	2013	2006	7
S55	Jiang Y et al.	2013	NA	NA
S56	Lu HY et al.	2013	2012	1
S57	Xie MJ et al.	2013	2011	2
S58	Zhong H,et al.	2012	2010	2
S59	Rezvan F,et al.	2012	2009	3
S60	Ang M,et al.	2012	2008	4
S61	Liu CS.	2012	NA	NA
S62	Liu LL et al.	2012	2011	1
S63	Yue JJ et al.	2012	2008	4
S64	Sherwin JC,et al.	2011	NA	NA
S65	Asokan R,et al.	2011	2003	8
S66	Yoon KC,et al.	2011	2009	2
S67	Landers J,et al.	2011	2007	4
S68	Tian BY et al.	2011	2003	8
S69	Chen YQ et al.	2011	2008	3
S70	Chen YQ et al.	2011	2000	11
S71	Liu QX et al.	2011	NA	NA
S72	Lei CT et al.	2011	2008	3
S73	Viso E,et al.	2010	2005	5
S74	Liang QF,et al.	2010	2009	1
S75	Feng WQ et al.	2010	2009	1
S76	Cajucom-Uy H,et al.	2009	NA	NA
S77	West S,et al.	2009	NA	NA
S78	Shiroma H,et al.	2009	2006	3
S79	Lu J,et al.	2009	2006	3
S80	Yu S et al.	2009	2007	2
S81	Wu XY et al.	2009	2007	2

ID	Author	Publication year	Study year	Time lag
S82	Gao XN et al.	2009	2007	2
S83	He Q et al.	2008	2007	1
S84	Qi QG et al.	2008	NA	NA
S85	Wu H.	2008	NA	NA
S86	Durkin SR,et al.	2007	2005	2
S87	Ma K,et al.	2007	2001	6
S88	Lu P,et al.	2007	2006	1
S89	Li J.	2007	2006	1
S90	Tan CS,et al.	2006	NA	NA
S91	Paula JS,et al.	2006	1998	8
S92	Cao XC.	2006	2005	1
S93	Han SX et al.	2005	2004	1
S94	Wu K,et al.	2002	1997	5
S95	Gazzard G,et al.	2002	2001	1
S96	Chen YQ et al.	2002	2001	1
S97	Wong TY,et al.	2001	1998	3
S98	Luthra R,et al.	2001	1990	11
S99	Liu H,et al.	2001	1999	2
S100	McCarty CA,et al.	2000	NA	NA
S101	Fang Y.	1999	1999	0
S102	Panchapakesan J,et al.	1998	1993	5
S103	Newland HS,et al.	1994	1991	3

Note: The average time-lag between investigation and publication was 3.90 based on 81 articles with available data.

Table S2. Country or territory list in World Bank and World Health Organization regions.

Region	Country or territories
**HICs-AMR**	Anguilla, Antigua and Barbuda, Aruba, Bahamas, Barbados, Bermuda, Bonaire, Sint Eustatius and Saba, British Virgin Islands, Canada, Cayman Islands, Chile, Curaçao, Falkland Islands (Malvinas), French Guiana, Guadeloupe, Guyana, Martinique, Montserrat, Panama, Puerto Rico, Saint Barthélemy, Saint Kitts and Nevis, Saint Martin (French part), Saint Pierre and Miquelon, Sint Maarten (Dutch part), Trinidad and Tobago, Turks and Caicos Islands, United States of America, United States Virgin Islands, Uruguay
**HICs-EMR**	Bahrain, Kuwait, Oman, Qatar, Saudi Arabia, United Arab Emirates
**HICs-EUR**	Andorra, Austria, Belgium, Bulgaria, Croatia, Cyprus, Czechia, Denmark, Estonia, Faroe Islands, Finland, France, Germany, Gibraltar, Greece, Greenland, Guernsey, Holy See, Hungary, Iceland, Ireland, Isle of Man, Israel, Italy, Jersey, Latvia, Liechtenstein, Lithuania, Luxembourg, Malta, Monaco, Netherlands, Norway, Poland, Portugal, Romania, Russian Federation, San Marino, Slovakia, Slovenia, Spain, Sweden, Switzerland, United Kingdom
**HICs-WPR**	American Samoa, Australia, Brunei Darussalam, China, Hong Kong SAR, China, Macao SAR, China, Taiwan Province of China, Cook Islands, French Polynesia, Guam, Japan, Nauru, New Caledonia, New Zealand, Niue, Northern Mariana Islands, Palau, Republic of Korea, Singapore, Tokelau, Wallis and Futuna Islands
**LMICs-AFR**	Algeria, Angola, Benin, Botswana, Burkina Faso, Burundi, Cabo Verde, Cameroon, Central African Republic, Chad, Comoros, Congo, Côte d'Ivoire, Democratic Republic of the Congo, Equatorial Guinea, Eritrea, Eswatini, Ethiopia, Gabon, Gambia, Ghana, Guinea, Guinea-Bissau, Kenya, Lesotho, Liberia, Madagascar, Malawi, Mali, Mauritania, Mauritius, Mayotte*, Mozambique, Namibia, Niger, Nigeria, Réunion*, Rwanda, Saint Helena*, Sao Tome and Principe, Senegal, Seychelles*, Sierra Leone, South Africa, South Sudan, Togo, Uganda, United Republic of Tanzania, Zambia, Zimbabwe
**LMICs-AMR**	Argentina, Belize, Bolivia (Plurinational State of), Brazil, Colombia, Costa Rica, Cuba, Dominica, Dominican Republic, Ecuador, El Salvador, Grenada, Guatemala, Haiti, Honduras, Jamaica, Mexico, Nicaragua, Paraguay, Peru, Saint Lucia, Saint Vincent and the Grenadines, Suriname, Venezuela (Bolivarian Republic of)
**LMICs-EMR**	Afghanistan, Djibouti, Egypt, Iran (Islamic Republic of), Iraq, Jordan, Lebanon, Libya, Morocco, Pakistan, Somalia, State of Palestine, Sudan, Syrian Arab Republic, Tunisia, Western Sahara, Yemen
**LMICs-EUR**	Albania, Armenia, Azerbaijan, Belarus, Bosnia and Herzegovina, Georgia, Kazakhstan, Kosovo (under UNSC res. 1244), Kyrgyzstan, Montenegro, North Macedonia, Republic of Moldova, Serbia, Tajikistan, Türkiye, Turkmenistan, Ukraine, Uzbekistan
**LMICs-SEAR**	Bangladesh, Bhutan, Dem. People's Republic of Korea, India, Indonesia, Maldives, Myanmar, Nepal, Sri Lanka, Thailand, Timor-Leste
**LMICs-WPR**	Cambodia, China, Fiji, Kiribati, Lao People's Democratic Republic, Malaysia, Marshall Islands, Micronesia (Fed. States of), Mongolia, Papua New Guinea, Philippines, Samoa, Solomon Islands, Tonga, Tuvalu, Vanuatu, Viet Nam

Note: HICs, high-income countries; LMICs, low- and middle-income countries; AFR, African Region; AMR, Region of the Americas; EMR, Eastern Mediterranean Region; EUR, European Region; SEAR, South-East Asia Region; WPR, Western Pacific Region. * Mayotte, Réunion, Saint Helena, and Seychelles are high-income country or territory according to the latest World Bank income classification, but classified into the low- and middle-income African Region due to its relatively small population size.

Table S3. Quality assessment scale.

	Criteria
1.	Was the sample representative of the target population?
2.	Were study participants recruited in an appropriate way?
3.	Was the sample size adequate?
4.	Were the study subjects and the setting described in detail?
5.	Was the data analysis conducted with sufficient coverage of the identified sample?
6.	Were objective, standard criteria used for the measurement of the condition?
7.	Was the condition measured reliably?
8.	Was there appropriate statistical analysis?
9.	Are all important confounding factors/subgroups/differences identified and accounted for?

Notes: Each questions was scored from zero (no) to one point (yes), with a total score ranging from zero to nine.

Table S4. Age- and sex- adjusted meta-regression models of study year category for prevalence of pterygium.

Variable	Number of data points	*β* (95% CI)	*P*-value
Intercept	590	**-3.7532 (-4.2247, -3.2817)**	**<0.0001**
Average age	590	**0.0338 (0.0325, 0.0351)**	**<0.0001**
Female proportion	590	-0.0525 (-0.1700, 0.0650)	0.3811
Study year			
<2008	289	**Reference**	
≥2008	301	-0.0144 (-0.0356, 0.0067)	0.1816
*Average age * Female proportion*	590	-0.0006 (-0.0026, 0.0015)	0.5811

Notes: CI, confidence interval.

Table S5. Multilevel mixed-effects meta-regression models for global prevalence of pterygium.

Variable	Number of data points	β (95% CI)	*P*-value
Intercept	590	**-4.1408 (-4.6679, -3.6137)**	**<0.0001**
Average age			
Average age₁	590	**0.0359 (0.0306, 0.0413)**	**<0.0001**
Average age₂	590	**0.0790 (0.0637, 0.0943)**	**<0.0001**
Average age₃	590	**-0.5768 (-0.6499, -0.5036)**	**<0.0001**
Average age₄	590	**0.9226 (0.7982, 1.0470)**	**<0.0001**
Female proportion	590	**-0.1486 (-0.2663, -0.0310)**	**<0.0001**
Average age * Female proportion	590	0.0009 (-0.0012, 0.0029)	0.3972

Notes: CI, confidence interval. Average age_1-Average age_4 were variables generated in the process of restricted cubic spline, and the knots were 24.5, 45.5, 57.7, 69.5 and 84.5.

Table S6. Sensitivity analyses for zero-event substitution values in the global prevalence model.

Substitution value	Number of data points	β (95% CI)	*P*-value
**0.0001**			
Intercept		**-4.1406 (-4.6676, -3.6135)**	**<0.0001**
Average age			
Average age₁		**0.0359 (0.0305, 0.0413)**	**<0.0001**
Average age₂	**590**	**0.0790 (0.0637, 0.0943)**	**<0.0001**
Average age₃		**-0.5768 (-0.6500, -0.5036)**	**<0.0001**
Average age₄		**0.9227 (0.7983, 1.0471)**	**<0.0001**
Female proportion		**-0.1486 (-0.2663, -0.0310)**	**0.0133**
Average age * Female proportion		0.0009 (-0.0012, 0.0029)	0.3972
**0.001**			
Intercept		**-4.1410 (-4.6681, -3.6140)**	**<0.0001**
Average age			
Average age₁		**0.0359 (0.0306, 0.0413)**	**<0.0001**
Average age₂	**590**	**0.0790 (0.0637, 0.0943)**	**<0.0001**
Average age₃		**-0.5767 (-0.6499, -0.5035)**	**<0.0001**
Average age₄		**0.9225 (0.7983, 1.0449)**	**<0.0001**
Female proportion		**-0.1486 (-0.2663, -0.0310)**	**0.0133**
Average age * Female proportion		0.0009 (-0.0012, 0.0029)	0.3971

Notes: CI, confidence interval. Average age$_1$-Average age$_4$ were variables generated in the process of restricted cubic spline, and the knots were 24.5, 45.5, 57.7, 69.5 and 84.5.

Table S7. Multilevel mixed-effects meta-regression models of the national prevalence of pterygium.

Variable	Number of data points	*β* (95% CI)	*P*-value
HICs male			
Intercept	940	**-3.9955 (-5.4774, -2.5135)**	**<0.0001**
Age			
Age_1	940	**0.0367 (0.0363, 0.0371)**	**<0.0001**
Age_2	940	**0.0574 (0.0561, 0.0588)**	**<0.0001**
Age_3	940	**0.0574 (0.0561, 0.0588)**	**<0.0001**
Age_4	940	**0.8170 (0.8026, 0.8314)**	**<0.0001**
Absolute value of latitude			
0-19.9	168	**Reference**	
20-29.9	365	0.2587 (-1.4217, 1.9391)	0.7628
30-34.9	91	-0.6591 (-2.3395, 1.0214)	0.4421
≥35	316	**-2.0248 (-3.7052, -0.3444)**	**0.0182**
HICs female			
Intercept	940	**-4.1330 (-5.5925, -2.6735)**	**<0.0001**
Age			
Age_1	940	**0.0360 (0.0356, 0.0365)**	**<0.0001**
Age_2	940	**0.0636 (0.0622, 0.0650)**	**<0.0001**
Age_3	940	**-0.5320 (-0.5404, -0.5236)**	**<0.0001**
Age_4	940	**0.8305 (0.8156, 0.8454)**	**<0.0001**
Absolute value of latitude			
0-19.9	168	**Reference**	
20-29.9	365	0.0234 (-1.6316, 1.6783)	0.9779
30-34.9	91	-0.7997 (-2.4548, 0.5853)	0.3436
≥35	316	**-2.0615 (-3.7165, -0.4065)**	**0.0146**
LMICs male			
Intercept	4951	**-4.4205 (-4.9234, -3.9176)**	**<0.0001**
Age			
Age_1	4951	**0.0633 (0.0626, 0.0640)**	**<0.0001**
Age_2	4951	**-0.0110 (-0.0129, -0.0092)**	**<0.0001**
Age_3	4951	**-0.1981 (-0.2088, -0.1874)**	**<0.0001**
Age_4	4951	**0.4008 (0.3816, 0.4200)**	**<0.0001**
Absolute value of latitude			
0-19.9	814	**Reference**	
20-29.9	1624	**0.0105 (0.0027, 0.0184)**	**0.0082**
30-34.9	748	**-0.7216 (-0.7301, -0.7132)**	**<0.0001**
≥35	1765	**-1.1229 (-1.1312, -1.1147)**	**<0.0001**
LMICs female			
Intercept	4935	**-4.1310 (-4.6802, -3.5818)**	**<0.0001**
Age			
Age_1	4935	**0.0564 (0.0558, 0.0571)**	**<0.0001**
Age_2	4935	**0.0128 (0.0111, 0.0146)**	**<0.0001**
Age_3	4935	**-0.3751 (-0.3851, -0.3650)**	**<0.0001**
Age_4	4935	**0.7348 (0.7169, 0.7527)**	**<0.0001**
Absolute value of latitude			

Variable	Number of data points	*β* (95% CI)	*P*-value
0-19.9	814	**Reference**	
20-29.9	1624	**-0.1100 (-0.1172, -0.1028)**	**<0.0001**
30-34.9	748	**-0.8626 (-0.8705, -0.8548)**	**<0.0001**
≥35	1749	**-1.3771 (-1.3847, -1.3695)**	**<0.0001**

Notes: HICs, high-income countries; LMICs, low- and middle-income countries; CI, confidence interval. Age_1-Age_4 were variables generated in the process of restricted cubic spline, and the knots were 23,46,58,70, and 84.

Table S8. Associated factors of pterygium.

Associated factor	Number of studies	Total sample	Pooled OR (95% CI)	*P* value	Significant in the largest study	95% PI	I^2	Small-study effects (*P*)	Excess significance bias (*P*)	Credibility	GRADE	Forest plot	Funnel plot	Sensitivity analysis
Age (per 1 year)	14	56348	1.03 (1.02, 1.03)	<0.001	Yes	(0.99, 1.06)	0.92	0.34	0.17	II	Low	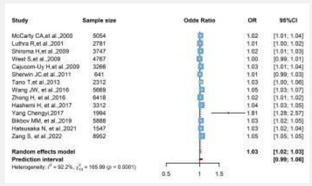	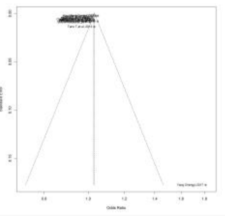	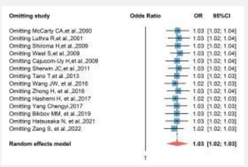
Age (60-69 vs 50-59)	5	52143	1.41 (1.23, 1.61)	<0.001	No	(1.00, 1.98)	0.47	0.4	0.63	III	Low	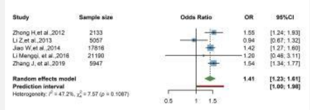	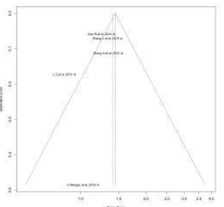	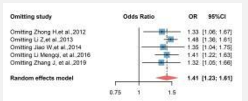
Age (70-79 vs 50-59)	4	47086	1.67 (1.50, 1.85)	<0.001	No	(1.34, 2.07)	0	0.77	0.54	III	Low	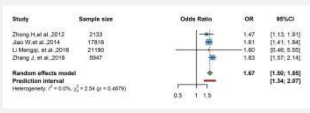	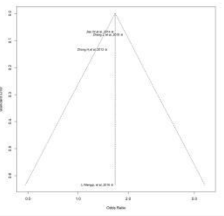	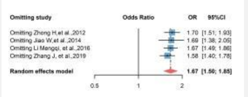
Age (>=80 vs 50-59)	4	47086	1.69 (1.37, 2.07)	<0.001	No	(1.02, 2.79)	0.12	0.88	0.38	III	Low	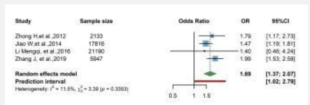	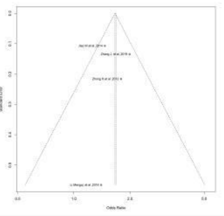	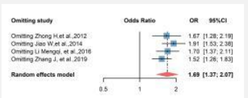
Age (50-59 vs 40-49)	8	38207	1.44 (1.01, 2.05)	0.042	No	(0.46, 4.48)	0.84	0.53	0.11	IV	Very low	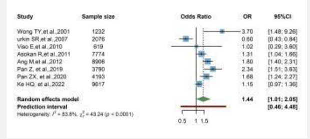	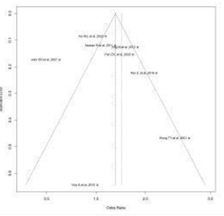	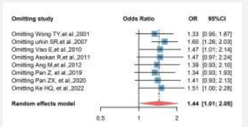
Age (60-69 vs 40-49)	8	38207	2.21 (1.47, 3.32)	<0.001	Yes	(0.57, 8.52)	0.88	0.28	0.45	II	Low	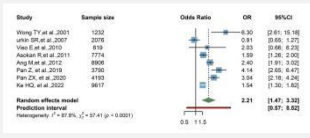	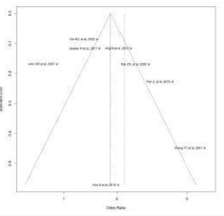	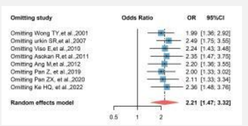
Age (70-79 vs 40-49)	8	31566	2.24 (1.62, 3.10)	<0.001	Yes	(0.83, 6.08)	0.79	0.04	0.17	II	Very low	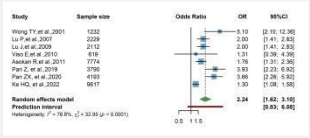	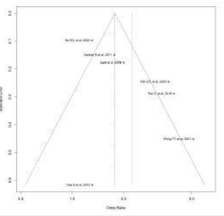	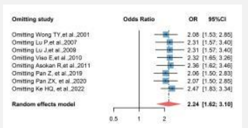
Age (>=80 vs 40-49)	3	18010	2.24 (0.98, 5.08)	0.055	No	(0.08, 61.47)	0.84	0.14	0.45	NS	Low	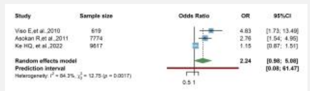	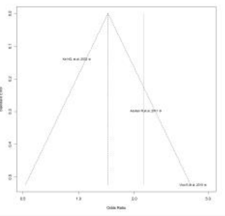	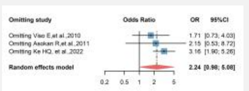
Age (40-49 vs 30-39)	5	34459	2.17 (1.61, 2.93)	<0.001	Yes	(0.96, 4.90)	0.58	0.59	0.43	II	Low	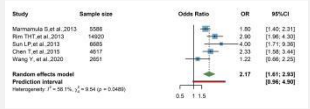	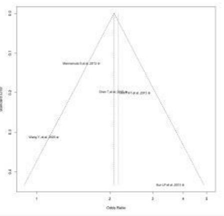	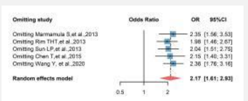
Age (50-59 vs 30-39)	5	34459	3.78 (2.68, 5.31)	<0.001	Yes	(1.41, 10.12)	0.68	0.13	0.33	II	Low	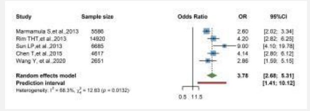	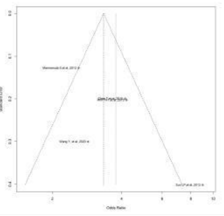	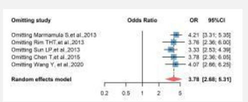
Age (60-69 vs 30-39)	4	31808	5.66 (3.12, 10.24)	<0.001	Yes	(0.76, 42.03)	0.87	0.09	0.36	II	Low	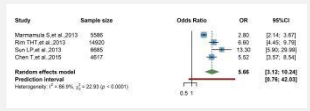	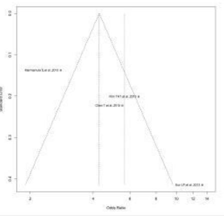	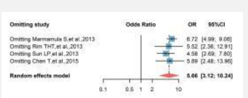

Associated factor	Number of studies	Total sample	Pooled OR (95% CI)	*P* value	Significant in the largest study	95% PI	I^2	Small-study effects (*P*)	Excess significance bias (*P*)	Credibility	GRADE	Forest plot	Funnel plot	Sensitivity analysis
Age (>=70 vs 30-39)	3	27191	8.15 (3.39, 19.57)	<0.001	Yes	(0.21, 312.05)	0.87	0.42	0.38	II	Moderate	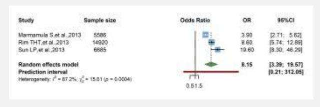	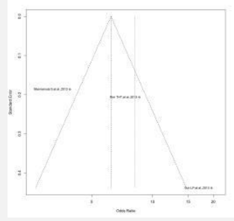	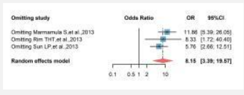
Sex (male)	37	168491	1.26 (1.04, 1.51)	0.017	No	(0.44, 3.86)	0.92	0.97	0.12	IV	Very low	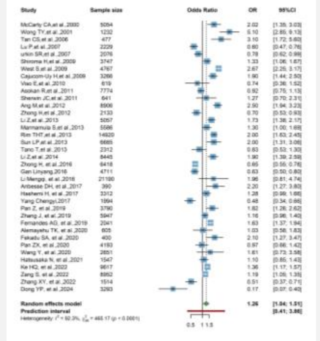	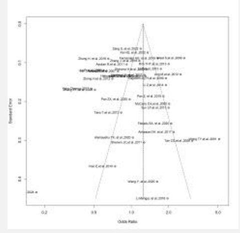	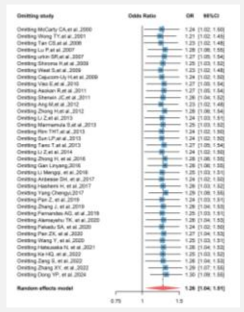
Setting (rural)	8	49670	2.20 (1.52, 3.18)	<0.001	Yes	(0.61, 7.90)	0.94	0.13	0.71	II	Low	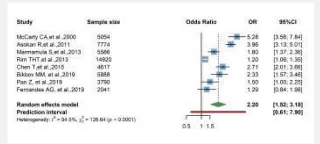	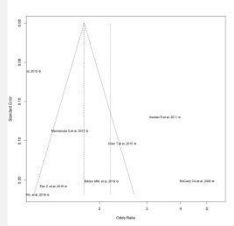	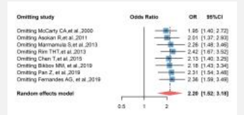
Education experience (any vs none)	3	14137	0.71 (0.59, 0.87)	0.001	Yes	(0.34, 1.49)	0.65	0.34	0.65	III	Very low	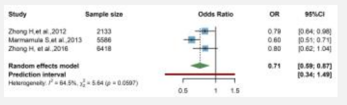	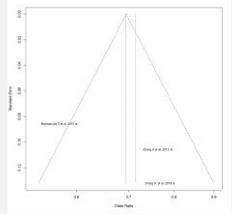	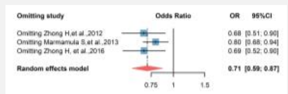
Education level (elementary vs none)	7	30153	0.81 (0.70, 0.94)	0.006	Yes	(0.56, 1.17)	0.41	0.74	0.62	IV	Low	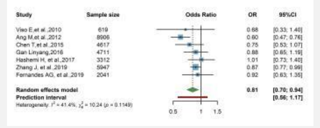	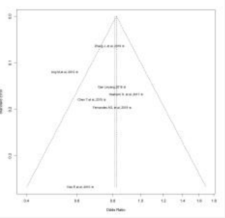	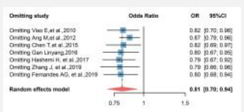
Education level (middle vs none)	3	15275	0.70 (0.60, 0.81)	<0.001	Yes	(0.49, 0.98)	0	0.68	0.48	I	Low	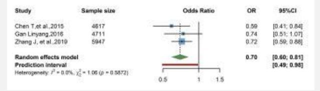	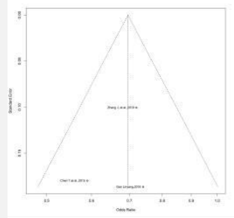	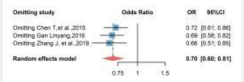
Education level (high vs none)	4	21546	0.51 (0.42, 0.62)	<0.001	Yes	(0.38, 0.70)	0	0.86	0.22	I	Low	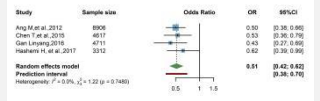	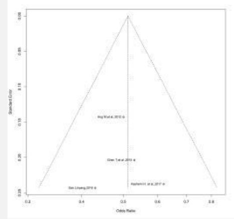	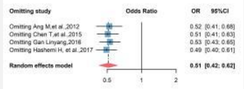
Drinking	8	33223	1.31 (1.07, 1.59)	0.007	No	(0.74, 2.31)	0.73	0.48	0.35	IV	Very low	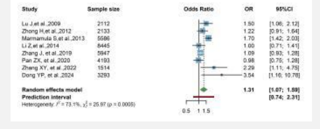	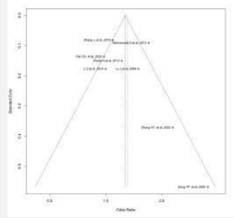	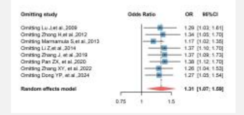
Smoking (past vs never)	4	10608	1.15 (0.95, 1.40)	0.16	No	(0.76, 1.74)	0	0.3	0.81	NS	Low	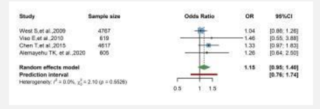	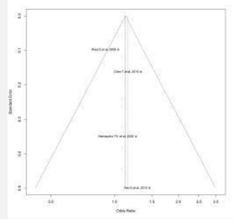	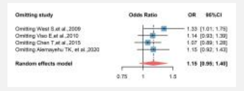
Smoking (current vs never)	4	10608	0.82 (0.68, 0.99)	0.039	Yes	(0.60, 1.14)	0	0.48	0.38	IV	Low	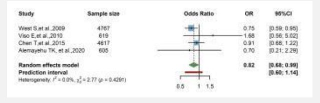	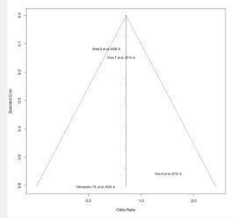	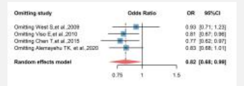
Smoking (current vs past+never)	4	18176	0.84 (0.44, 1.58)	0.58	Yes	(0.09, 7.92)	0.95	0.34	0.31	NS	Very low	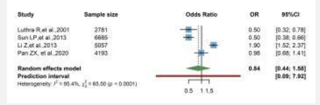	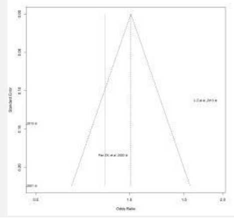	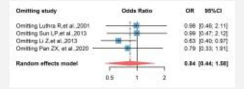

Associated factor	Number of studies	Total sample	Pooled OR (95% CI)	*P* value	Significant in the largest study	95% PI	I^2	Small-study effects (*P*)	Excess significance bias (*P*)	Credibility	GRADE	Forest plot	Funnel plot	Sensitivity analysis
Smoking (current+past vs never)	15	17288	0.91 (0.80, 1.04)	0.183	Yes	(0.57, 1.46)	0.66	0.82	0.36	NS	Very low	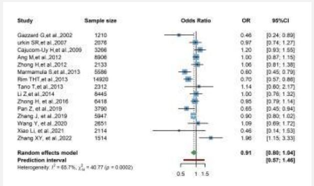	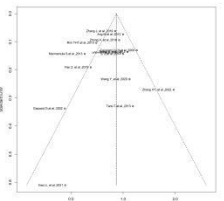	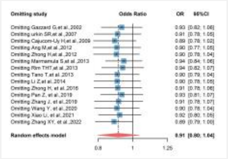
Hypertension	10	38173	1.09 (0.99, 1.20)	0.067	No	(0.91, 1.31)	0.39	0.15	0.06	NS	Very low	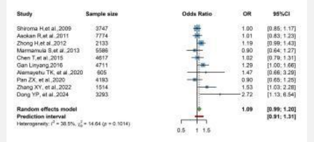	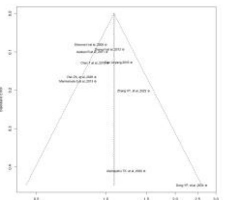	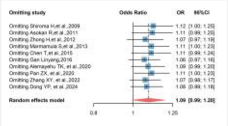
Diabetes	9	31426	1.00 (0.67, 1.49)	0.996	No	(0.27, 3.74)	0.76	0.7	0.4	NS	Very low	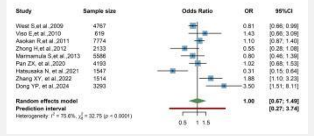	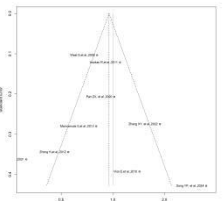	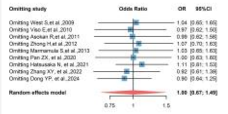
Dry eye symptoms	5	14471	1.37 (1.10, 1.71)	0.006	Yes	(0.76, 2.47)	0.56	0.7	0.62	IV	Very low	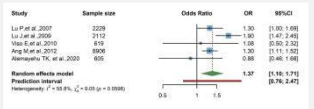	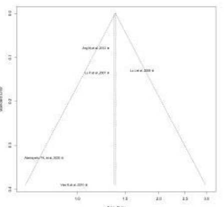	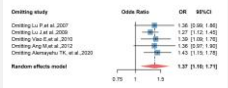
HDL-C (per 1 mmol/L)	3	16935	1.23 (1.08, 1.40)	0.002	Yes	(0.92, 1.63)	0	0.67	0.55	IV	Low	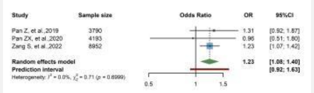	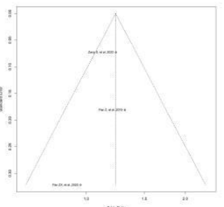	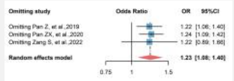
Weight (per 1 kg)	3	10497	1.01 (1.00, 1.01)	0.043	No	(0.99, 1.03)	0.22	0	0.07	IV	Very low	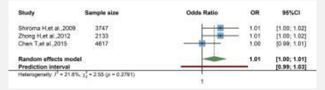	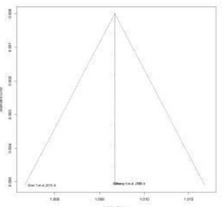	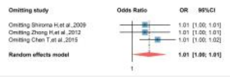
Height (per 1 cm)	3	10497	0.99 (0.98, 1.00)	0.166	No	(0.97, 1.02)	0	0.64	0.74	NS	Low	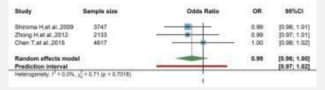	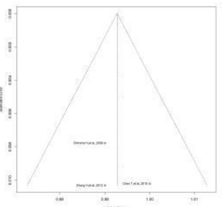	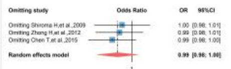
Sun exposure (>=5h/day)	3	15925	2.63 (0.96, 7.24)	0.06	Yes	(0.04, 196.99)	0.95	0.33	0.18	NS	Very low	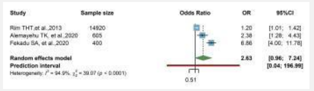	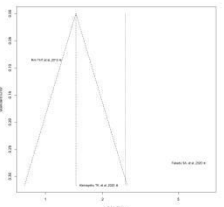	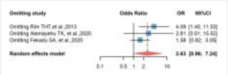
Outdoor job	20	84004	1.66 (1.38, 1.99)	<0.001	No	(0.73, 3.73)	0.82	0.37	0.49	III	Very low	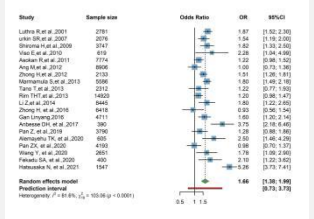	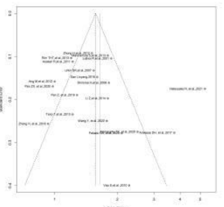	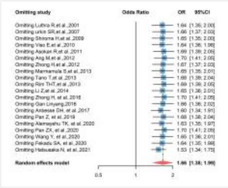
Spectacles use	3	16653	0.73 (0.62, 0.87)	<0.001	Yes	(0.51, 1.06)	0.07	0.36	0.29	II	Low	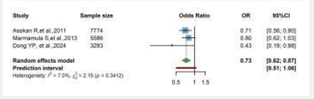	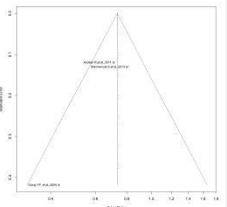	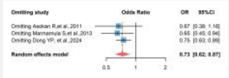
Use of sunshade products	8	36342	0.47 (0.29, 0.74)	<0.001	Yes	(0.10, 2.22)	0.87	0.03	0.37	II	Very low	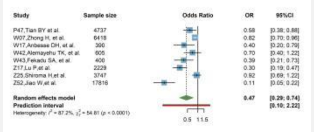	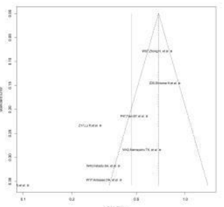	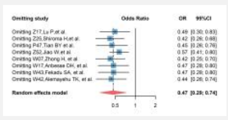

Notes: HDL-C, high-density lipoprotein cholesterol; OR, odds ratio; CI, confidence interval; PI, prediction interval.

Table S9. Evidence credibility grading criteria.

Category	Criteria
Convincing evidence (class I)	● *P*-value < 1×10^{-6}<br>● More than 1000 cases<br>● 95% prediction interval excluding the null value<br>● I^2 < 50%<br>● No small-study effects (*P*-value > 0.1) and excess significance bias (*P*-value > 0.1)
Highly suggestive evidence (class II)	● *P*-value < 1×10^{-6}<br>● More than 1000 cases<br>●A statistically significant result reported in the largest individual study
Suggestive evidence (class III)	● *P*-value < 1×10^{-3}<br>● More than 1000 cases
Weak evidence (class IV)	● *P*-value < 0.05
Non-significant (NS)	● *P*-value > 0.05

Table S10. Evidence quality grading criteria by GRADE.

Study Design	Quality of Evidence	Lower if	Higher if
**Randomized trial →**	**High**	**Risk of bias**<br>-1 Serious<br>-2 Very serious	**Large effect**<br>+1 Large<br>+2 Very large
		**Inconsistency**<br>-1 Serious	**Dose response**<br>+1 Evidence of a gradient
	**Moderate**	-2 Very serious	
		**Indirectness**<br>-1 Serious<br>-2 Very serious	**All plausible confounding**<br>+1 Would reduce demonstrated effect<br>or<br>+1 Would suggest a spurious effect when results show no effect
**Observational study →**	**Low**	**Imprecision**<br>-1 Serious<br>-2 Very serious	
		**Publication bias**	
	**Very low**	-1 Likely<br>-2 Very likely	

Table S11. Detailed characteristics of the included articles (n=103).

ID	Author	Publication year	Country	WHO Region	WB region	Setting	Study year	Community-based or health check-based	Female proportion	Age range	Sample size	Case
S01	Dong YP, et al.	2024	China	WPR	LMICs	Mixed	November 2021	Community-based	0.5008	0+	3293	405
S02	Adriano L, et al.	2022	Brazil	AMR	LMICs	Mixed	July 2016 and June to August 2017	Community-based	0.6983	40+	600	123
S03	Zhang XY, et al.	2022	China	WPR	LMICs	Mixed	June 2020 to June 2021	Community-based	0.4974	0+	1514	210
S04	Zang S, et al.	2022	China	WPR	LMICs	Rural	September 2011 to February 2012	Community-based	0.5985	40+	8952	1619
S05	Tandon R, et al.	2022	India	SEAR	LMICs	Rural	2010 to 2016	Community-based	0.5454	40+	9735	1287
S06	Padhy D, et al.	2022	India	SEAR	LMICs	Rural	NA	Community-based	0.6429	5+	126	15
S07	Ke HQ, et al.	2022	China	WPR	LMICs	Rural	March to November 2019	Community-based	0.6830	40-97	9617	2113
S08	Xiao Li, et al.	2021	China	WPR	LMICs	Mixed	October 2015 to June 2018	Community-based	0.7025	50+	2114	489
S09	Hatsusaka N, et al.	2021	China	WPR	LMICs	Mixed	March 5–12, 2006, July 14-21, 2006, and July 2-12, 2009	Community-based	0.6141	50-92	1547	361
S10	Zhang Yanling, et al.	2020	China	WPR	LMICs	Mixed	April to August 2019	Community-based	0.7866	50-89	942	87
S11	Liu Yanjun.	2020	China	WPR	LMICs	Mixed	January to October 2019	Health check	0.5439	50+	4190	142
S12	Wang Y, et al.	2020	China	WPR	LMICs	Mixed	July to August 2014	Community-based	0.6066	30-79	2651	169
S13	Pan ZX, et al.	2020	China	WPR	LMICs	Mixed	July 5 to August 14, 2016	Community-based	0.5612	40-79	4193	391
S14	Fekadu SA, et al.	2020	Ethiopia	AFR	LMICs	Mixed	April 15 to May 3, 2019	Community-based	0.4525	20+	400	127
S15	Alemayehu TK, et al.	2020	Ethiopia	AFR	LMICs	Mixed	May 30 to June 16, 2019	Community-based	0.4760	18-95	605	112
S16	Fernandes AG, et al.	2019	Brazil	AMR	LMICs	Mixed	NA	Community-based	0.5100	45+	2041	1199
S17	Wu Xiaolan, et al.	2019	China	WPR	LMICs	Mixed	March 2015 to July 2016	Community-based	NA	50-106	5448	781
S18	Zhang J, et al.	2019	China	WPR	LMICs	Rural	NA	Community-based	0.5852	50+	5947	1950
S19	Pan Z, et al.	2019	China	WPR	LMICs	Mixed	July 19 to September 12, 2017	Community-based	0.5995	40-79	3790	248
S20	Lin YH, et al.	2019	Taiwan, China	WPR	HICs	Mixed	2000 to 2011	Community-based	0.5042	0+	999975	22063

ID	Author	Publication year	Country	WHO Region	WB region	Setting	Study year	Community-based or health check-based	Female proportion	Age range	Sample size	Case
S21	Bikbov MM, et al.	2019	Russia	EUR	LMICs	Mixed	NA	Community-based	NA	40+	5888	138
S22	Zhao Lizhen, et al.	2017	China	WPR	LMICs	Mixed	January 2016 to December 2016	Health check	0.5000	20-50	22124	295
S23	Yang Chengyi.	2017	China	WPR	LMICs	Rural	January 2015 to March 2016	Community-based	0.6394	0+	1994	777
S24	Su Xiaolong, et al.	2017	China	WPR	LMICs	Mixed	NA	Community-based	0.4972	20-80	12746	629
S25	Sitompul R, et al.	2017	Indonesia	SEAR	LMICs	Rural	July 2016	Community-based	0.5877	0-80	667	72
S26	Rim TH, et al.	2017	Republic of Korea	WPR	HICs	Mixed	2004 to 2013	Community-based	NA	0+	10089825	187638
S27	Hashemi H, et al.	2017	Iran	EMR	LMICs	Rural	2015	Community-based	0.5631	2-93	3312	442
S28	Cao XG, et al.	2017	China	WPR	LMICs	Rural	August 2003 and May 2004	Community-based	0.6308; 0.6123	50-89	2496	600
S29	Anbesse DH, et al.	2017	Ethiopia	AFR	LMICs	Mixed	April 15 to May 7, 2016	Community-based	0.5692	20-88	390	151
S30	Li Ming, et al.	2016	China	WPR	LMICs	Rural	2009	Community-based	0.4989	30+	21478	295
S31	Li Mengqi, et al.	2016	China	WPR	LMICs	Mixed	April 2015 to January 2016	Community-based	0.5971	50+	21190	1115
S32	Gan Linyang.	2016	China	WPR	LMICs	Mixed	July to August 2015	Community-based	0.5336	8-83	4711	391
S33	Bi Yun, et al.	2016	China	WPR	LMICs	Mixed	June to August 2015	Health check	0.6389	45+	468	98
S34	Zhong H, et al.	2016	China	WPR	LMICs	Rural	NA	Community-based	0.6016	50+	6418	2312
S35	Wang JW, et al.	2016	China	WPR	LMICs	Rural	NA	Community-based	0.4357	50-91	5669	246
S36	Lin AD, et al.	2016	United States of America	AMR	HICs	Mixed	January, February, June and July 2012	Community-based	0.4260	18-80	169	19
S37	Chen T,et al.	2015	China	WPR	LMICs	Mixed	NA	Community-based	0.6125	30+	4617	546
S38	Ma WZ.	2015	China	WPR	LMICs	Mixed	April to July 2008	Community-based	0.5732	50+	4866	455
S39	Ma F.	2015	China	WPR	LMICs	Rural	January to April 2013	Community-based	0.4844	35+	4379	404
S40	Cui XH et al.	2015	China	WPR	LMICs	Rural	June to August 2010	Community-based	0.6620	50+	1506	341
S41	Shrestha S,et al.	2014	Nepal	SEAR	LMICs	Mixed	2010 to 2011	Community-based	0.5784	20+	408	111
S42	Maharjan IM,et al.	2014	Nepal	SEAR	LMICs	Rural	NA	Community-based	0.5171	16+	1319	133
S43	Li Z,et al.	2014	China	WPR	LMICs	Rural	November 2008 to July 2009	Community-based	0.4913	18-94	8445	208

ID	Author	Publication year	Country	WHO Region	WB region	Setting	Study year	Community-based or health check-based	Female proportion	Age range	Sample size	Case
S44	Jiao W,et al.	2014	China	WPR	LMICs	Rural	April to July 2008	Community-based	0.5620	50-101	17816	1876
S45	Jiang ZY et al.	2014	China	WPR	LMICs	Rural	NA	Community-based	0.5027	50-95	3300	650
S46	Li CJ et al.	2014	China	WPR	LMICs	Mixed	July 2012 to July 2013	Community-based	0.2860	60-86	584	57
S47	Luo ZL et al.	2014	China	WPR	LMICs	Mixed	March to December 2013	Community-based	0.4385	40+	3393	843
S48	Shen FR.	2014	China	WPR	LMICs	Rural	NA	Community-based	0.5017	50+	6769	596
S49	Tano T,et al.	2013	Japan	WPR	HICs	Urban	April 13 and June 17, 2009	Community-based	0.5921	40-74	2312	101
S50	Sun LP,et al.	2013	China	WPR	LMICs	Rural	October 2006 to October 2007	Community-based	0.4631	30+	6685	401
S51	Rim THT,et al.	2013	Republic of Korea	WPR	HICs	Mixed	July 2008 to 2010	Health check	0.5711	30+	14920	1219
S52	Nangia V,et al.	2013	India	SEAR	LMICs	Rural	NA	Community-based	0.5349	30+	4711	608
S53	Marmamula S,et al.	2013	India	SEAR	LMICs	Mixed	1996 to 2000	Community-based	0.5356	30-102	5586	655
S54	Li Z,et al.	2013	China	WPR	LMICs	Rural	2006	Community-based	0.5288	50-96	5057	323
S55	Jiang Y et al.	2013	China	WPR	LMICs	Mixed	NA	Health check	0.4635	40+	5707	547
S56	Lu HY et al.	2013	China	WPR	LMICs	Rural	April to May 2012	Community-based	0.5081	40-92	7268	2170
S57	Xie MJ et al.	2013	China	WPR	LMICs	Urban	May 2011 to December 2011	Community-based	0.5824	40+	7478	401
S58	Zhong H,et al.	2012	China	WPR	LMICs	Rural	January to April 2010	Community-based	0.6395	50-92	2133	832
S59	Rezvan F,et al.	2012	Iran	EMR	LMICs	Urban	2009	Community-based	0.5736	40-64	5190	489
S60	Ang M,et al.	2012	Singapore	WPR	HICs	Mixed	2004 to 2011	Community-based	0.5057	40-80	8906	900
S61	Liu CS.	2012	China	WPR	LMICs	Mixed	NA	Community-based	0.5192	16-80	16357	974
S62	Liu LL et al.	2012	China	WPR	LMICs	Rural	May to July 2011	Community-based	0.6689	50-93	3494	280
S63	Yue JJ et al.	2012	China	WPR	LMICs	Mixed	March to November 2008	Community-based	NA	35+	2987	494

ID	Author	Publication year	Country	WHO Region	WB region	Setting	Study year	Community-based or health check-based	Female proportion	Age range	Sample size	Case
S64	Sherwin JC,et al.	2011	Australia	WPR	HICs	Mixed	2007	Community-based	0.5647	15-89	641	70
S65	Asokan R,et al.	2011	India	SEAR	LMICs	Mixed	2001 to 2004	Community-based	0.5534	40+	7774	740
S66	Yoon KC,et al.	2011	Republic of Korea	WPR	HICs	Mixed	July 2008 to December 2009	Health check	0.5718	19+	11014	594
S67	Landers J,et al.	2011	Australia	WPR	HICs	Rural	July 2005 and June 2008	Community-based	0.6343	20+	1884	147
S68	Tian BY et al.	2011	China	WPR	LMICs	Rural	July to December 2003	Community-based	0.5698	20+	4737	416
S69	Chen YQ et al.	2011	China	WPR	LMICs	Urban	October 2007 to September 2010	Community-based	0.5587	16-83	16818	628
S70	Chen YQ et al.	2011	China	WPR	LMICs	Rural	April to May 2000	Community-based	0.4455	11-94	10890	968
S71	Liu QX et al.	2011	China	WPR	LMICs	Mixed	NA	Community-based	0.5275	0+	3001	185
S72	Lei CT et al.	2011	China	WPR	LMICs	Rural	April to June 2008	Community-based	0.6134	50+	3288	435
S73	Viso E,et al.	2010	Spain	EUR	HICs	Mixed	May 2005 to March 2006	Community-based	0.6300	40-96	619	42
S74	Liang QF,et al.	2010	China	WPR	LMICs	Rural	June 2008 to August 2009	Community-based	0.6953	55-85	37067	1395
S75	Feng WQ et al.	2010	China	WPR	LMICs	Rural	July to October 2009	Community-based	0.5160	55-87	1750	585
S76	Cajucom-Uy H,et al.	2009	Singapore	WPR	HICs	Urban	August 2004 to June 2006	Community-based	0.5187	40-79	3266	508
S77	West S,et al.	2009	United States	AMR	HICs	Mixed	NA	Community-based	0.6119	40+	4767	774
S78	Shiroma H,et al.	2009	Japan	WPR	HICs	Mixed	May 1, 2005 to August 31, 2006	Community-based	0.5129	40+	3747	1154
S79	Lu J,et al.	2009	China	WPR	LMICs	Rural	June to September 2006	Community-based	0.4673	40+	2112	378
S80	Yu S et al.	2009	China	WPR	LMICs	Urban	2007	Community-based	0.4286	0+	1740	404
S81	Wu XY et al.	2009	China	WPR	LMICs	Rural	March 2007 to April 2008	Community-based	0.4639	40+	6245	560
S82	Gao XN et al.	2009	China	WPR	LMICs	Rural	August to October 2007	Community-based	0.4838	40+	4568	478

ID	Author	Publication year	Country	WHO Region	WB region	Setting	Study year	Community-based or health check-based	Female proportion	Age range	Sample size	Case
S83	He Q et al.	2008	China	WPR	LMICs	Rural	July to September 2007	Community-based	0.4978	18-80	8054	1745
S84	Qi QG et al.	2008	China	WPR	LMICs	Mixed	NA	Community-based	0.6102	2-88	2558	134
S85	Wu H.	2008	China	WPR	LMICs	Rural	NA	Community-based	0.4221	6-99	353	49
S86	Durkin SR,et al.	2007	Myanmar	SEAR	LMICs	Rural	November 2005	Community-based	0.5973	40+	2076	407
S87	Ma K,et al.	2007	China	WPR	LMICs	Mixed	2001	Community-based	0.5643	40-101	4439	128
S88	Lu P,et al.	2007	China	WPR	LMICs	Rural	October to December 2006	Community-based	0.4401	40+	2229	323
S89	Li J.	2007	China	WPR	LMICs	Rural	February to August 2006	Community-based	0.5376	40+	6455	410
S90	Tan CS,et al.	2006	Indonesia	SEAR	LMICs	Mixed	NA	Community-based	0.5577	0-90	477	81
S91	Paula JS,et al.	2006	Brazil	AMR	LMICs	Mixed	1997 to 1999	Community-based	0.5721	18+	624	115
S92	Cao XC.	2006	China	WPR	LMICs	Rural	March to June 2005	Community-based	0.4620	1-58	619	214
S93	Han SX et al.	2005	China	WPR	LMICs	Rural	March to May 2004	Community-based	0.4888	40+	7527	679
S94	Wu K,et al.	2002	China	WPR	LMICs	Rural	1997	Community-based	0.5510	50-97	4214	1391
S95	Gazzard G,et al.	2002	Indonesia	SEAR	LMICs	Mixed	April to June 2001	Community-based	0.4942	21+	1210	112
S96	Chen YQ et al.	2002	China	WPR	LMICs	Rural	September to October 2001	Community-based	0.5074	50-93	3368	671
S97	Wong TY,et al.	2001	Singapore	WPR	HICs	Mixed	October 10, 1997 to August 14, 1998	Community-based	0.5487	40-81	1232	120
S98	Luthra R,et al.	2001	Barbados	AMR	HICs	Mixed	1988 to 1992	Community-based	0.5500	40-84	2781	642
S99	Liu H,et al.	2001	China	WPR	LMICs	Rural	July to August 1999	Community-based	0.4819	11-88	7990	628
S100	McCarty CA,et al.	2000	Australia	WPR	HICs	Mixed	NA	Community-based	0.5518	40-101	5054	141
S101	Fang Y.	1999	China	WPR	LMICs	Mixed	October 1998 to April 1999	Community-based	0.4891	0+	101973	2787
S102	Panchapakesan J,et al.	1998	Australia	WPR	HICs	Mixed	January 1992 to January 1994	Community-based	NA	49+	3654	266

ID	Author	Publication year	Country	WHO Region	WB region	Setting	Study year	Community-based or health check-based	Female proportion	Age range	Sample size	Case
S103	Newland HS,et al.	1994	Tonga	WPR	LMICs	Mixed	July 1991	Community-based	0.5515	20+	4056	1050

Notes: NA, not available; WHO, World Health Organization; WB, World Bank; HICs, high-income countries; LMICs, low- and middle-income countries; AFR, African Region; AMR, Region of the Americas; EMR, Eastern Mediterranean Region; EUR, European Region; SEAR, South-East Asia Region; WPR, Western Pacific Region.

Table S12. Quality scores for the included articles (n=103).

ID	Author	Year Published	Was the sample representative of the target population?	Were study participants recruited in an appropriate way?	Was the sample size adequate?	Were the study subjects and setting described in detail?	Was the data analysis conducted with sufficient coverage of the identified sample?	Were objective, standard criteria used for measurement of the condition?	Was the condition measured reliably?	Was there appropriate statistical analysis?	Are all important confounding factors/ subgroups/differences identified and accounted for?	Total scores
S01	Dong YP, et al.	2024	1	1	0	1	0	1	1	1	1	7
S02	Adriano L, et al.	2022	1	1	1	1	0	1	1	1	1	8
S03	Zhang XY, et al.	2022	1	1	1	1	0	1	1	1	1	8
S04	Zang S, et al.	2022	1	0	0	1	1	1	1	1	1	7
S05	Tandon R, et al.	2022	1	1	0	1	1	1	1	1	1	8
S06	Padhy D, et al.	2022	1	0	0	1	0	1	1	0	1	5
S07	Ke HQ, et al.	2022	1	1	1	1	1	1	1	1	1	9
S08	Xiao Li, et al.	2021	1	1	1	1	1	1	1	1	1	9
S09	Hatsusaka N, et al.	2021	1	0	0	1	0	1	1	1	1	6
S10	Zhang Yanling, et al.	2020	1	1	0	1	1	1	1	1	1	8
S11	Liu Yanjun.	2020	1	0	0	1	0	1	1	0	0	4
S12	Wang Y, et al.	2020	1	1	0	1	1	1	1	1	1	8
S13	Pan ZX, et al.	2020	1	1	0	1	1	1	1	1	1	8
S14	Fekadu SA, et al.	2020	1	1	1	1	1	1	1	1	1	9
S15	Alemayehu TK, et al.	2020	1	1	1	1	1	1	1	1	1	9
S16	Fernandes AG, et al.	2019	1	1	0	1	1	1	1	1	1	8
S17	Wu Xiaolan, et al.	2019	1	1	1	0	1	1	1	1	0	7
S18	Zhang J, et al.	2019	1	1	1	1	1	1	1	1	1	9

ID	Author	Year Published	Was the sample representative of the target population?	Were study participants recruited in an appropriate way?	Was the sample size adequate?	Were the study subjects and setting described in detail?	Was the data analysis conducted with sufficient coverage of the identified sample?	Were objective, standard criteria used for measurement of the condition?	Was the condition measured reliably?	Was there appropriate statistical analysis?	Are all important confounding factors/ subgroups/differences identified and accounted for?	Total scores
S19	Pan Z, et al.	2019	1	1	0	1	1	1	0	1	1	7
S20	Lin YH, et al.	2019	1	1	0	0	1	1	0	1	1	6
S21	Bikbov MM, et al.	2019	1	0	0	0	1	1	1	1	1	6
S22	Zhao Lizhen, et al.	2017	0	0	0	1	0	1	0	1	1	4
S23	Yang Chengyi.	2017	1	1	0	0	1	1	1	1	1	7
S24	Su Xiaolong, et al.	2017	1	1	1	1	0	1	1	1	1	8
S25	Sitompul R, et al.	2017	1	1	1	1	0	1	1	0	0	6
S26	Rim TH, et al.	2017	1	1	1	1	0	1	1	1	1	8
S27	Hashemi H, et al.	2017	1	1	0	1	1	1	1	1	1	8
S28	Cao XG, et al.	2017	1	1	1	1	1	1	1	1	1	9
S29	Anbesse DH, et al.	2017	1	1	1	1	1	1	1	1	1	9
S30	Li Ming, et al.	2016	1	1	0	1	1	1	1	1	0	7
S31	Li Mengqi, et al.	2016	1	1	0	1	1	1	1	1	1	8
S32	Gan Linyang.	2016	1	1	0	1	1	1	0	1	1	7
S33	Bi Yun, et al.	2016	0	0	0	1	0	1	1	1	1	5
S34	Zhong H, et al.	2016	1	1	0	1	1	1	1	1	1	8
S35	Wang JW, et al.	2016	1	1	0	1	1	1	1	1	1	8
S36	Lin AD, et al.	2016	1	0	0	1	0	1	0	1	1	5
S37	Chen T,et al.	2015	1	1	0	1	1	1	0	1	1	7
S38	Ma WZ.	2015	1	1	1	1	1	1	1	1	1	9

ID	Author	Year Published	Was the sample representative of the target population?	Were study participants recruited in an appropriate way?	Was the sample size adequate?	Were the study subjects and setting described in detail?	Was the data analysis conducted with sufficient coverage of the identified sample?	Were objective, standard criteria used for measurement of the condition?	Was the condition measured reliably?	Was there appropriate statistical analysis?	Are all important confounding factors/ subgroups/differences identified and accounted for?	Total scores
S39	Ma F.	2015	1	1	0	1	1	1	0	0	1	6
S40	Cui XH et al.	2015	1	1	1	1	1	1	1	1	1	9
S41	Shrestha S,et al.	2014	1	0	0	0	1	1	0	0	1	4
S42	Maharjan IM,et al.	2014	1	1	1	0	0	1	0	0	1	5
S43	Li Z,et al.	2014	1	1	1	0	1	1	1	1	1	8
S44	Jiao W,et al.	2014	1	1	1	1	1	1	1	1	1	9
S45	Jiang ZY et al.	2014	1	1	0	1	1	1	0	0	1	6
S46	Li CJ et al.	2014	0	0	0	1	1	1	0	1	1	5
S47	Luo ZL et al.	2014	1	1	0	1	1	1	1	0	1	7
S48	Shen FR.	2014	1	1	1	1	1	1	0	0	1	7
S49	Tano T,et al.	2013	1	0	0	1	1	1	0	1	1	6
S50	Sun LP,et al.	2013	1	1	0	0	1	1	0	1	1	6
S51	Rim THT,et al.	2013	1	1	0	0	1	1	0	1	1	6
S52	Nangia V,et al.	2013	1	0	0	0	1	1	1	1	1	6
S53	Marmamula S,et al.	2013	1	1	0	1	1	1	1	1	1	8
S54	Li Z,et al.	2013	1	1	1	0	1	1	1	1	1	8
S55	Jiang Y et al.	2013	0	1	0	1	1	1	1	0	1	6
S56	Lu HY et al.	2013	1	0	0	1	1	1	0	0	1	5
S57	Xie MJ et al.	2013	1	1	0	1	1	1	1	1	1	8
S58	Zhong H,et al.	2012	1	1	0	0	1	1	0	1	1	6
S59	Rezvan F,et al.	2012	1	1	0	0	1	1	0	1	1	6
S60	Ang M,et al.	2012	0	1	0	1	0	1	1	1	1	6

ID	Author	Year Published	Was the sample representative of the target population?	Were study participants recruited in an appropriate way?	Was the sample size adequate?	Were the study subjects and setting described in detail?	Was the data analysis conducted with sufficient coverage of the identified sample?	Were objective, standard criteria used for measurement of the condition?	Was the condition measured reliably?	Was there appropriate statistical analysis?	Are all important confounding factors/ subgroups/differences identified and accounted for?	Total scores
S61	Liu CS.	2012	1	0	0	0	1	1	1	1	1	6
S62	Liu LL et al.	2012	1	1	1	1	1	1	1	1	1	9
S63	Yue JJ et al.	2012	1	0	0	0	1	1	0	0	1	4
S64	Sherwin JC,et al.	2011	0	0	0	1	0	1	0	1	1	4
S65	Asokan R,et al.	2011	1	1	0	1	1	1	0	1	1	7
S66	Yoon KC,et al.	2011	1	0	0	0	0	1	1	1	1	5
S67	Landers J,et al.	2011	0	0	0	1	0	1	0	1	1	4
S68	Tian BY et al.	2011	1	1	0	1	1	1	1	1	1	8
S69	Chen YQ et al.	2011	1	1	0	0	1	1	1	0	1	6
S70	Chen YQ et al.	2011	1	1	0	1	1	1	0	0	1	6
S71	Liu QX et al.	2011	1	1	0	1	1	1	0	0	1	6
S72	Lei CT et al.	2011	1	1	0	1	1	1	1	0	1	7
S73	Viso E,et al.	2010	0	1	0	1	1	1	1	1	1	7
S74	Liang QF,et al.	2010	1	0	0	1	0	1	1	1	1	6
S75	Feng WQ et al.	2010	1	1	0	1	1	1	0	1	1	7
S76	Cajucom-Uy H,et al.	2009	1	1	0	0	1	1	1	1	1	7
S77	West S,et al.	2009	1	1	0	0	0	1	0	1	1	5
S78	Shiroma H,et al.	2009	0	1	1	1	1	1	0	1	1	7
S79	Lu J,et al.	2009	1	1	1	0	1	1	0	1	1	7
S80	Yu S et al.	2009	1	1	0	1	1	1	0	1	1	7
S81	Wu XY et al.	2009	1	1	0	1	1	1	1	0	1	7
S82	Gao XN et al.	2009	1	1	0	1	1	1	0	0	1	6

ID	Author	Year Published	Was the sample representative of the target population?	Were study participants recruited in an appropriate way?	Was the sample size adequate?	Were the study subjects and setting described in detail?	Was the data analysis conducted with sufficient coverage of the identified sample?	Were objective, standard criteria used for measurement of the condition?	Was the condition measured reliably?	Was there appropriate statistical analysis?	Are all important confounding factors/ subgroups/differences identified and accounted for?	Total scores
S83	He Q et al.	2008	1	1	0	1	1	1	1	0	1	7
S84	Qi QG et al.	2008	1	1	0	1	1	1	0	0	1	6
S85	Wu H.	2008	1	1	1	1	1	1	1	1	1	9
S86	Durkin SR,et al.	2007	1	1	0	0	1	1	0	1	1	6
S87	Ma K,et al.	2007	1	1	0	0	1	1	0	0	1	5
S88	Lu P,et al.	2007	1	1	0	0	1	1	1	1	1	7
S89	Li J.	2007	1	1	1	1	1	1	0	0	1	7
S90	Tan CS,et al.	2006	0	0	0	1	1	1	0	1	1	5
S91	Paula JS,et al.	2006	1	1	0	0	0	1	0	1	1	5
S92	Cao XC.	2006	1	1	0	1	1	1	1	0	1	7
S93	Han SX et al.	2005	1	1	0	1	1	1	0	0	1	6
S94	Wu K,et al.	2002	0	1	0	1	1	1	0	0	1	5
S95	Gazzard G,et al.	2002	1	1	1	0	1	1	1	1	1	8
S96	Chen YQ et al.	2002	1	1	0	1	1	1	0	0	1	6
S97	Wong TY,et al.	2001	0	1	0	1	0	1	0	1	1	5
S98	Luthra R,et al.	2001	0	1	0	0	1	1	0	1	1	5
S99	Liu H,et al.	2001	1	1	0	0	1	1	0	1	1	6
S100	McCarty CA,et al.	2000	1	1	0	1	1	1	0	1	1	7
S101	Fang Y.	1999	1	1	1	0	1	1	1	0	1	7
S102	Panchapakesan J,et al.	1998	0	1	0	0	1	1	0	0	1	4
S103	Newland HS,et al.	1994	1	1	1	1	1	1	0	1	0	7

Notes: Each questions was scored from zero (no) to one point (yes), with a total score ranging from zero to nine.

Table S13. Age- and sex-specific prevalence and case number of pterygium by World Bank and World Health Organization region.

Age (years)	Prevalence (%, 95% CI)			Case number (million, 95% CI)		
	Both	Male	Female	Both	Male	Female
**HICs-AMR**						
10-19	0.50 (0.20, 1.29)	0.62 (0.24, 1.65)	0.37 (0.16, 0.90)	0.27 (0.11, 0.69)	0.17 (0.07, 0.46)	0.10 (0.04, 0.23)
20-29	0.59 (0.24, 1.48)	0.71 (0.28, 1.84)	0.46 (0.20, 1.09)	0.33 (0.14, 0.83)	0.21 (0.08, 0.54)	0.12 (0.05, 0.29)
30-39	0.78 (0.32, 1.92)	0.91 (0.37, 2.31)	0.63 (0.28, 1.49)	0.47 (0.19, 1.15)	0.28 (0.11, 0.72)	0.18 (0.08, 0.43)
40-49	1.30 (0.54, 3.12)	1.49 (0.60, 3.67)	1.09 (0.48, 2.53)	0.70 (0.29, 1.68)	0.42 (0.17, 1.02)	0.28 (0.12, 0.66)
50-59	2.87 (1.21, 6.53)	3.31 (1.34, 7.66)	2.41 (1.07, 5.34)	1.51 (0.64, 3.43)	0.89 (0.36, 2.06)	0.62 (0.27, 1.37)
60-69	3.83 (1.66, 8.35)	4.25 (1.79, 9.42)	3.41 (1.54, 7.31)	1.92 (0.83, 4.20)	1.06 (0.44, 2.34)	0.87 (0.39, 1.85)
70-79	4.37 (1.93, 9.34)	4.71 (2.01, 10.3)	4.05 (1.86, 8.49)	1.44 (0.64, 3.09)	0.73 (0.31, 1.61)	0.71 (0.32, 1.48)
80-89	5.45 (2.47, 11.31)	5.86 (2.54, 12.47)	5.14 (2.41, 10.45)	0.75 (0.34, 1.55)	0.34 (0.15, 0.72)	0.40 (0.19, 0.82)
**10-89**	**1.98 (0.85, 4.45)**	**2.17 (0.90, 5.01)**	**1.78 (0.80, 3.89)**	**7.38 (3.18, 16.62)**	**4.10 (1.70, 9.48)**	**3.28 (1.48, 7.14)**
**HICs-EMR**						
10-19	2.44 (1.16, 5.02)	3.10 (1.45, 6.45)	1.75 (0.85, 3.52)	0.19 (0.09, 0.39)	0.12 (0.06, 0.26)	0.07 (0.03, 0.13)
20-29	3.07 (1.47, 6.19)	3.58 (1.70, 7.27)	2.17 (1.07, 4.32)	0.33 (0.16, 0.67)	0.25 (0.12, 0.50)	0.09 (0.04, 0.17)
30-39	4.01 (1.94, 7.96)	4.49 (2.15, 8.94)	2.96 (1.47, 5.83)	0.58 (0.28, 1.15)	0.44 (0.21, 0.89)	0.14 (0.07, 0.27)
40-49	6.15 (3.02, 11.83)	6.79 (3.31, 13.06)	4.75 (2.38, 9.16)	0.57 (0.28, 1.11)	0.43 (0.21, 0.84)	0.14 (0.07, 0.27)
50-59	12.44 (6.38, 22.19)	13.62 (6.97, 24.13)	10.08 (5.20, 18.31)	0.59 (0.30, 1.04)	0.43 (0.22, 0.76)	0.16 (0.08, 0.29)
60-69	16.87 (8.89, 28.81)	18.41 (9.70, 31.12)	14.52 (7.67, 25.29)	0.31 (0.17, 0.54)	0.21 (0.11, 0.35)	0.11 (0.06, 0.19)
70-79	19.35 (10.33, 32.57)	20.88 (11.10, 34.81)	17.60 (9.44, 29.98)	0.12 (0.07, 0.21)	0.07 (0.04, 0.12)	0.05 (0.03, 0.09)
80-89	23.89 (13.01, 39.17)	25.63 (13.90, 41.57)	22.25 (12.18, 36.9)	0.05 (0.02, 0.07)	0.02 (0.01, 0.04)	0.02 (0.01, 0.04)
**10-89**	**5.51 (2.75, 10.40)**	**6.21 (3.07, 11.75)**	**4.27 (2.17, 8.01)**	**2.74 (1.37, 5.18)**	**1.98 (0.98, 3.74)**	**0.77 (0.39, 1.44)**
**HICs-EUR**						
10-19	0.42 (0.20, 0.90)	0.51 (0.24, 1.11)	0.33 (0.16, 0.68)	0.32 (0.15, 0.68)	0.20 (0.09, 0.43)	0.12 (0.06, 0.25)
20-29	0.50 (0.24, 1.06)	0.59 (0.27, 1.27)	0.41 (0.20, 0.84)	0.38 (0.18, 0.80)	0.23 (0.11, 0.49)	0.15 (0.07, 0.31)
30-39	0.67 (0.32, 1.43)	0.77 (0.36, 1.67)	0.57 (0.28, 1.19)	0.63 (0.30, 1.34)	0.36 (0.17, 0.79)	0.27 (0.13, 0.55)

Age (years)	Prevalence (%, 95% CI)			Case number (million, 95% CI)		
	Both	Male	Female	Both	Male	Female
40-49	1.13 (0.53, 2.43)	1.27 (0.58, 2.77)	0.99 (0.48, 2.08)	1.08 (0.51, 2.32)	0.61 (0.28, 1.32)	0.48 (0.23, 1.00)
50-59	2.47 (1.16, 5.22)	2.75 (1.27, 5.87)	2.20 (1.06, 4.60)	2.34 (1.10, 4.95)	1.27 (0.59, 2.72)	1.07 (0.51, 2.23)
60-69	3.48 (1.64, 7.28)	3.78 (1.75, 7.99)	3.23 (1.55, 6.66)	3.01 (1.42, 6.28)	1.51 (0.70, 3.19)	1.50 (0.72, 3.09)
70-79	4.03 (1.93, 8.27)	4.23 (1.98, 8.84)	3.87 (1.89, 7.84)	2.43 (1.16, 5.00)	1.11 (0.52, 2.31)	1.33 (0.65, 2.69)
80-89	5.04 (2.47, 10.05)	5.22 (2.49, 10.61)	4.93 (2.45, 9.71)	1.57 (0.77, 3.12)	0.61 (0.29, 1.24)	0.96 (0.48, 1.88)
**10-89**	**1.92 (0.91, 4.00)**	**1.99 (0.92, 4.21)**	**1.85 (0.90, 3.80)**	**11.76 (5.58, 24.50)**	**5.90 (2.74, 12.48)**	**5.86 (2.84, 12.01)**
**HICs-WPR**						
10-19	1.13 (0.52, 2.48)	1.43 (0.65, 3.17)	0.81 (0.38, 1.75)	0.26 (0.12, 0.57)	0.17 (0.08, 0.38)	0.09 (0.04, 0.20)
20-29	1.35 (0.62, 2.97)	1.65 (0.74, 3.66)	1.03 (0.48, 2.22)	0.38 (0.17, 0.82)	0.24 (0.11, 0.53)	0.14 (0.06, 0.30)
30-39	1.84 (0.86, 3.92)	2.17 (1.00, 4.66)	1.50 (0.71, 3.16)	0.56 (0.26, 1.19)	0.33 (0.15, 0.72)	0.22 (0.11, 0.47)
40-49	2.69 (1.27, 5.53)	3.11 (1.45, 6.40)	2.280 (1.10, 4.67)	0.94 (0.45, 1.94)	0.55 (0.25, 1.12)	0.40 (0.19, 0.82)
50-59	5.30 (2.59, 10.23)	6.02 (2.93, 11.56)	4.58 (2.26, 8.91)	1.91 (0.93, 3.68)	1.08 (0.53, 2.08)	0.83 (0.41, 1.61)
60-69	7.51 (3.75, 13.98)	8.31 (4.14, 15.38)	6.72 (3.38, 12.62)	2.34 (1.17, 4.35)	1.27 (0.63, 2.35)	1.06 (0.53, 2.00)
70-79	7.28 (3.67, 13.50)	7.96 (3.98, 14.69)	6.69 (3.40, 12.45)	1.88 (0.95, 3.48)	0.96 (0.48, 1.77)	0.92 (0.47, 1.71)
80-89	7.93 (4.07, 14.43)	8.81 (4.49, 15.96)	7.33 (3.79, 13.41)	1.14 (0.59, 2.08)	0.51 (0.26, 0.92)	0.63 (0.33, 1.16)
**10-89**	**4.21 (2.07, 8.11)**	**4.63 (2.26, 8.94)**	**3.79 (1.89, 7.29)**	**9.4 (4.63, 18.12)**	**5.11 (2.49, 9.87)**	**4.29 (2.14, 8.25)**
**LMICs-AFR**						
10-19	3.45 (2.13, 5.53)	3.57 (2.21, 5.71)	3.32 (2.05, 5.35)	9.75 (6.01, 15.63)	5.06 (3.13, 8.09)	4.69 (2.88, 7.54)
20-29	5.03 (3.15, 7.92)	5.18 (3.26, 8.13)	4.88 (3.05, 7.70)	10.45 (6.55, 16.46)	5.37 (3.37, 8.42)	5.09 (3.18, 8.04)
30-39	7.83 (4.97, 12.09)	8.00 (5.09, 12.32)	7.66 (4.85, 11.87)	12.01 (7.63, 18.55)	6.06 (3.86, 9.34)	5.95 (3.77, 9.21)
40-49	13.18 (8.53, 19.81)	13.34 (8.65, 20.00)	13.03 (8.42, 19.63)	14.01 (9.07, 21.06)	6.96 (4.51, 10.44)	7.05 (4.55, 10.62)
50-59	25.02 (16.8, 35.69)	25.06 (16.86, 35.68)	24.98 (16.75, 35.7)	16.89 (11.34, 24.1)	8.11 (5.46, 11.56)	8.78 (5.89, 12.55)
60-69	33.01 (22.67, 45.64)	32.79 (22.56, 45.26)	33.19 (22.77, 45.96)	13.23 (9.09, 18.3)	6.04 (4.15, 8.33)	7.20 (4.94, 9.97)
70-79	37.99 (26.37, 51.75)	37.45 (26.05, 50.89)	38.41 (26.62, 52.41)	6.91 (4.79, 9.41)	2.98 (2.07, 4.05)	3.93 (2.72, 5.36)
80-89	44.91 (31.61, 59.99)	43.74 (30.88, 58.26)	45.67 (32.09, 61.11)	2.19 (1.54, 2.93)	0.84 (0.59, 1.12)	1.35 (0.95, 1.81)
**10-89**	**9.70 (6.36, 14.35)**	**9.54 (6.26, 14.14)**	**9.85 (6.46, 14.56)**	**85.44 (56.03, 126.43)**	**41.42 (27.15, 61.34)**	**44.02 (28.88, 65.09)**
**LMICs-AMR**						

Age (years)	Prevalence (%, 95% CI)			Case number (million, 95% CI)		
	Both	Male	Female	Both	Male	Female
10-19	2.17 (1.34, 3.48)	2.28 (1.41, 3.64)	2.06 (1.27, 3.32)	2.18 (1.34, 3.49)	1.16 (0.72, 1.86)	1.01 (0.62, 1.63)
20-29	3.20 (2.01, 5.04)	3.34 (2.10, 5.23)	3.06 (1.91, 4.84)	3.23 (2.03, 5.09)	1.70 (1.07, 2.66)	1.53 (0.96, 2.42)
30-39	5.07 (3.22, 7.83)	5.24 (3.34, 8.06)	4.91 (3.11, 7.61)	4.82 (3.06, 7.44)	2.47 (1.57, 3.8)	2.35 (1.49, 3.64)
40-49	8.55 (5.54, 12.85)	8.75 (5.68, 13.1)	8.36 (5.4, 12.61)	7.03 (4.55, 10.57)	3.5 (2.27, 5.24)	3.53 (2.28, 5.33)
50-59	16.25 (10.92, 23.16)	16.44 (11.08, 23.36)	16.07 (10.77, 22.97)	10.74 (7.22, 15.31)	5.21 (3.51, 7.4)	5.53 (3.71, 7.91)
60-69	21.28 (14.62, 29.42)	21.34 (14.7, 29.41)	21.23 (14.56, 29.42)	10.16 (6.98, 14.05)	4.76 (3.28, 6.56)	5.40 (3.70, 7.49)
70-79	24.17 (16.78, 32.93)	24.12 (16.79, 32.75)	24.21 (16.76, 33.08)	6.29 (4.37, 8.58)	2.78 (1.93, 3.77)	3.52 (2.44, 4.81)
80-89	28.92 (20.39, 38.58)	28.51 (20.17, 37.88)	29.2 (20.54, 39.04)	2.72 (1.92, 3.63)	1.07 (0.76, 1.43)	1.65 (1.16, 2.20)
**10-89**	**8.94 (5.96, 12.91)**	**8.77 (5.85, 12.66)**	**9.11 (6.07, 13.15)**	**47.17 (31.47, 68.14)**	**22.64 (15.11, 32.71)**	**24.53 (16.36, 35.43)**
**LMICs-EMR**						
10-19	1.95 (1.20, 3.15)	2.06 (1.27, 3.31)	1.84 (1.13, 2.98)	3.05 (1.87, 4.91)	1.64 (1.01, 2.64)	1.40 (0.86, 2.27)
20-29	2.87 (1.79, 4.56)	3.02 (1.88, 4.77)	2.72 (1.69, 4.35)	3.61 (2.25, 5.73)	1.93 (1.21, 3.05)	1.68 (1.04, 2.68)
30-39	4.48 (2.82, 7.03)	4.65 (2.94, 7.27)	4.30 (2.69, 6.79)	4.74 (2.98, 7.45)	2.49 (1.58, 3.89)	2.25 (1.41, 3.55)
40-49	7.62 (4.85, 11.75)	7.84 (5.01, 12.02)	7.39 (4.68, 11.49)	5.96 (3.79, 9.20)	3.08 (1.97, 4.72)	2.88 (1.82, 4.48)
50-59	14.89 (9.73, 22.05)	15.11 (9.94, 22.19)	14.66 (9.53, 21.91)	8.11 (5.31, 12.02)	4.08 (2.69, 6.00)	4.03 (2.62, 6.02)
60-69	19.97 (13.28, 28.85)	20.07 (13.43, 28.74)	19.87 (13.13, 28.96)	6.98 (4.64, 10.08)	3.38 (2.26, 4.84)	3.60 (2.38, 5.25)
70-79	22.21 (14.9, 31.69)	22.07 (14.9, 31.22)	22.33 (14.89, 32.09)	3.61 (2.42, 5.15)	1.65 (1.11, 2.34)	1.96 (1.31, 2.82)
80-89	25.41 (17.25, 35.64)	24.89 (17.02, 34.62)	25.77 (17.42, 36.36)	1.07 (0.72, 1.50)	0.43 (0.30, 0.60)	0.63 (0.43, 0.89)
**10-89**	**6.45 (4.17, 9.74)**	**6.45 (4.18, 9.69)**	**6.45 (4.15, 9.78)**	**37.13 (23.99, 56.04)**	**18.70 (12.12, 28.08)**	**18.43 (11.86, 27.96)**
**LMICs-EUR**						
10-19	0.85 (0.52, 1.38)	0.97 (0.60, 1.57)	0.72 (0.44, 1.19)	0.31 (0.19, 0.51)	0.18 (0.11, 0.29)	0.13 (0.08, 0.21)
20-29	1.29 (0.79, 2.08)	1.46 (0.91, 2.35)	1.10 (0.68, 1.80)	0.45 (0.28, 0.72)	0.26 (0.16, 0.42)	0.19 (0.12, 0.31)
30-39	2.11 (1.31, 3.41)	2.39 (1.48, 3.82)	1.84 (1.13, 3.00)	0.82 (0.51, 1.33)	0.47 (0.29, 0.75)	0.35 (0.22, 0.58)
40-49	3.74 (2.31, 6.02)	4.17 (2.60, 6.65)	3.31 (2.03, 5.40)	1.28 (0.79, 2.06)	0.71 (0.44, 1.12)	0.57 (0.35, 0.93)
50-59	7.73 (4.84, 12.17)	8.51 (5.38, 13.19)	7.02 (4.34, 11.23)	2.17 (1.36, 3.42)	1.15 (0.73, 1.78)	1.03 (0.63, 1.64)
60-69	10.57 (6.67, 16.39)	11.60 (7.41, 17.69)	9.72 (6.07, 15.32)	2.44 (1.54, 3.78)	1.21 (0.77, 1.84)	1.23 (0.77, 1.94)
70-79	11.90 (7.58, 18.20)	13.15 (8.47, 19.78)	11.07 (6.99, 17.16)	1.40 (0.89, 2.15)	0.61 (0.40, 0.92)	0.79 (0.50, 1.22)

Age (years)	Prevalence (%, 95% CI)			Case number (million, 95% CI)		
	Both	Male	Female	Both	Male	Female
80-89	14.30 (9.27, 21.39)	15.65 (10.27, 22.95)	13.70 (8.82, 20.68)	0.68 (0.44, 1.02)	0.23 (0.15, 0.34)	0.45 (0.29, 0.68)
**10-89**	**4.50 (2.83, 7.06)**	**4.68 (2.96, 7.25)**	**4.34 (2.70, 6.88)**	**9.56 (6.00, 14.99)**	**4.82 (3.05, 7.48)**	**4.74 (2.95, 7.51)**
**LMICs-SEAR**						
10-19	3.50 (2.16, 5.61)	3.78 (2.34, 6.03)	3.21 (1.97, 5.16)	12.81 (7.91, 20.53)	7.19 (4.45, 11.47)	5.63 (3.46, 9.06)
20-29	5.16 (3.23, 8.12)	5.54 (3.48, 8.68)	4.75 (2.97, 7.53)	18.58 (11.64, 29.25)	10.28 (6.46, 16.10)	8.30 (5.18, 13.15)
30-39	8.23 (5.23, 12.72)	8.75 (5.58, 13.44)	7.70 (4.87, 11.97)	26.79 (17.02, 41.40)	14.58 (9.30, 22.40)	12.21 (7.72, 18.99)
40-49	13.98 (9.05, 21.03)	14.66 (9.53, 21.92)	13.28 (8.55, 20.10)	37.57 (24.32, 56.50)	20.11 (13.07, 30.05)	17.47 (11.25, 26.44)
50-59	26.46 (17.76, 37.76)	27.37 (18.47, 38.80)	25.54 (17.05, 36.72)	54.63 (36.68, 77.98)	28.34 (19.13, 40.18)	26.30 (17.55, 37.80)
60-69	35.27 (24.22, 48.78)	36.16 (24.98, 49.67)	34.40 (23.48, 47.92)	49.40 (33.92, 68.33)	24.88 (17.19, 34.18)	24.52 (16.73, 34.15)
70-79	40.45 (28.06, 55.14)	41.25 (28.81, 55.82)	39.76 (27.42, 54.56)	27.29 (18.93, 37.21)	12.87 (8.98, 17.41)	14.43 (9.95, 19.80)
80-89	48.65 (34.28, 64.89)	48.93 (34.72, 64.75)	48.46 (33.98, 64.99)	10.26 (7.23, 13.69)	4.28 (3.04, 5.66)	5.99 (4.20, 8.03)
**10-89**	**13.52 (8.98, 19.65)**	**13.74 (9.15, 19.90)**	**13.30 (8.81, 19.39)**	**237.34 (157.64, 344.88)**	**122.51 (81.61, 177.46)**	**114.83 (76.03, 167.42)**
**LMICs-WPR**						
10-19	1.54 (0.95, 2.49)	1.65 (1.02, 2.66)	1.42 (0.87, 2.29)	3.44 (2.11, 5.54)	1.96 (1.21, 3.15)	1.48 (0.90, 2.39)
20-29	2.25 (1.40, 3.59)	2.41 (1.50, 3.82)	2.08 (1.29, 3.32)	4.82 (3.00, 7.68)	2.73 (1.71, 4.34)	2.09 (1.30, 3.35)
30-39	3.35 (2.10, 5.30)	3.62 (2.27, 5.70)	3.06 (1.91, 4.87)	9.14 (5.72, 14.45)	5.13 (3.22, 8.08)	4.01 (2.50, 6.37)
40-49	5.75 (3.62, 9.01)	6.21 (3.93, 9.68)	5.26 (3.30, 8.30)	13.44 (8.47, 21.07)	7.40 (4.68, 11.54)	6.04 (3.79, 9.53)
50-59	10.48 (6.70, 16.04)	11.31 (7.27, 17.16)	9.65 (6.13, 14.92)	27.76 (17.75, 42.49)	14.97 (9.62, 22.71)	12.80 (8.13, 19.78)
60-69	14.41 (9.34, 21.60)	15.40 (10.03, 22.88)	13.50 (8.70, 20.41)	24.54 (15.91, 36.79)	12.59 (8.21, 18.72)	11.95 (7.71, 18.07)
70-79	15.50 (10.09, 23.08)	16.63 (10.88, 24.56)	14.56 (9.44, 21.85)	15.61 (10.16, 23.24)	7.57 (4.95, 11.18)	8.04 (5.21, 12.06)
80-89	18.39 (12.16, 26.80)	19.25 (12.79, 27.83)	17.83 (11.75, 26.12)	6.12 (4.04, 8.91)	2.55 (1.69, 3.68)	3.57 (2.35, 5.23)
**10-89**	**6.93 (4.44, 10.59)**	**7.17 (4.61, 10.89)**	**6.69 (4.27, 10.28)**	**104.87 (67.18, 160.18)**	**54.90 (35.28, 83.39)**	**49.97 (31.89, 76.78)**

Notes: CI, confidence interval; WHO, World Health Organization; WB, World Bank; HICs, high-income countries; LMICs, low-income and middle-income countries; AFR, African Region; AMR, Region of the Americas; EMR, Eastern Mediterranean Region; EUR, European Region; SEAR, South-East Asia Region; WPR, Western Pacific Region. .

Table S14. National prevalence and case number of pterygium.

Country or territory	Prevalence (%, 95% CI)			Case number (thousand, 95% CI)		
	Both	Male	Female	Both	Male	Female
Afghanistan	5.09 (3.21, 7.94)	5.14 (3.26, 7.96)	5.04 (3.17, 7.92)	1467.75 (925.91, 2287.84)	743.77 (471.42, 1151.72)	723.98 (454.48, 1136.11)
Albania	5.45 (3.44, 8.49)	5.95 (3.79, 9.16)	4.97 (3.10, 7.85)	135.69 (85.55, 211.36)	72.83 (46.31, 112.02)	62.86 (39.24, 99.34)
Algeria	7.90 (5.24, 11.50)	8.01 (5.34, 11.60)	7.78 (5.14, 11.39)	2871.88 (1904.86, 4180.57)	1486.07 (990.03, 2152.16)	1385.82 (914.82, 2028.40)
American Samoa	7.09 (1.87, 21.09)	7.58 (1.96, 22.88)	6.58 (1.78, 19.27)	2.78 (0.73, 8.26)	1.50 (0.39, 4.53)	1.27 (0.35, 3.73)
Andorra	1.58 (0.74, 3.30)	1.68 (0.78, 3.58)	1.46 (0.71, 3.02)	1.17 (0.55, 2.46)	0.64 (0.30, 1.36)	0.53 (0.26, 1.09)
Angola	7.91 (5.20, 11.68)	7.76 (5.09, 11.47)	8.06 (5.30, 11.88)	1982.21 (1302.14, 2926.46)	953.98 (626.18, 1410.47)	1028.22 (675.96, 1515.99)
Anguilla	7.03 (1.88, 20.37)	7.40 (1.93, 21.74)	6.69 (1.83, 19.09)	0.91 (0.24, 2.64)	0.46 (0.12, 1.36)	0.45 (0.12, 1.28)
Antigua and Barbuda	15.43 (4.11, 44.91)	15.94 (4.14, 47.29)	14.97 (4.09, 42.76)	12.70 (3.39, 36.95)	6.20 (1.61, 18.40)	6.49 (1.77, 18.55)
Argentina	3.19 (2.00, 4.98)	3.35 (2.12, 5.18)	3.03 (1.89, 4.79)	1239.24 (779.23, 1938.93)	644.22 (408.07, 997.04)	595.02 (371.15, 941.9)
Armenia	5.01 (3.15, 7.85)	5.19 (3.29, 8.02)	4.87 (3.04, 7.71)	128.43 (80.69, 201.11)	60.68 (38.44, 93.87)	67.75 (42.25, 107.24)
Aruba	15.45 (4.16, 43.69)	15.89 (4.17, 45.61)	15.07 (4.15, 42.00)	14.86 (4.00, 42.01)	7.15 (1.88, 20.53)	7.71 (2.12, 21.48)
Australia	11.75 (6.08, 20.93)	13.33 (6.84, 23.69)	10.21 (5.34, 18.22)	2715.83 (1405.95, 4836.00)	1524.72 (782.64, 2710.24)	1191.11 (623.31, 2125.76)
Austria	2.34 (1.11, 4.89)	2.45 (1.14, 5.19)	2.24 (1.09, 4.60)	191.39 (90.70, 399.28)	98.35 (45.61, 208.49)	93.03 (45.08, 190.79)
Azerbaijan	4.21 (2.64, 6.62)	4.42 (2.79, 6.87)	4.01 (2.49, 6.38)	372.26 (233.23, 585.59)	189.32 (119.56, 294.33)	182.94 (113.66, 291.26)
Bahamas	9.63 (4.95, 17.39)	11.12 (5.66, 20.09)	8.28 (4.31, 14.95)	33.90 (17.41, 61.21)	18.60 (9.46, 33.60)	15.29 (7.95, 27.60)
Bahrain	6.14 (3.07, 11.49)	6.85 (3.40, 12.87)	4.88 (2.50, 9.06)	83.96 (42.06, 157.21)	59.73 (29.66, 112.22)	24.22 (12.39, 44.99)
Bangladesh	11.78 (7.80, 17.19)	12.3 (8.19, 17.83)	11.29 (7.43, 16.59)	16385.76(10849.23, 23919.08)	8332.45 (5547.60, 12081.28)	8053.31 (5301.62, 11837.8)
Barbados	16.62 (4.47, 47.21)	17.12 (4.49, 49.45)	16.17 (4.45, 45.16)	41.45 (11.15, 117.74)	20.35 (5.33, 58.79)	21.1 (5.82, 58.96)
Belarus	4.84 (3.04, 7.56)	4.85 (3.07, 7.49)	4.83 (3.02, 7.61)	392.52 (246.98, 613.2)	181.55 (115.11, 280.51)	210.97 (131.88, 332.69)
Belgium	1.44 (0.68, 3.00)	1.51 (0.70, 3.20)	1.36 (0.66, 2.79)	148.68 (70.44, 310.22)	77.21 (35.80, 163.67)	71.47 (34.64, 146.54)
Belize	12.12 (8.02, 17.70)	12.03 (7.96, 17.58)	12.20 (8.07, 17.83)	40.76 (26.97, 59.55)	20.36 (13.48, 29.75)	20.40 (13.49, 29.8)
Benin	9.94 (6.53, 14.65)	9.78 (6.43, 14.44)	10.09 (6.64, 14.87)	987.13 (648.99, 1455.76)	484.94 (318.63, 716.02)	502.19 (330.36, 739.74)
Bermuda	4.62 (2.26, 9.00)	5.06 (2.44, 9.95)	4.19 (2.09, 8.08)	2.70 (1.32, 5.26)	1.45 (0.70, 2.85)	1.25 (0.62, 2.41)
Bhutan	12.21 (8.08, 17.86)	12.68 (8.42, 18.47)	11.67 (7.68, 17.14)	83.22 (55.04, 121.66)	46.48 (30.85, 67.70)	36.73 (24.19, 53.97)

Country or territory	Prevalence (%, 95% CI)			Case number (thousand, 95% CI)		
	Both	Male	Female	Both	Male	Female
Bolivia (Plurinational State of)	9.66 (6.41, 14.08)	9.4 (6.23, 13.72)	9.92 (6.59, 14.43)	942.07 (624.83, 1372.36)	457.85 (303.43, 668.00)	484.21 (321.39, 704.37)
Bonaire, Sint Eustatius and Saba	11.43 (3.06, 32.66)	12.17 (3.19, 35.07)	10.64 (2.92, 30.09)	3.06 (0.82, 8.74)	1.68 (0.44, 4.85)	1.38 (0.38, 3.89)
Bosnia and Herzegovina	7.26 (4.58, 11.30)	7.53 (4.79, 11.59)	7.02 (4.39, 11.04)	209.74 (132.31, 326.37)	103.34 (65.69, 158.99)	106.40 (66.62, 167.38)
Botswana	7.21 (4.74, 10.65)	7.45 (4.91, 10.97)	6.97 (4.57, 10.33)	138.74 (91.12, 204.84)	71.34 (46.99, 104.97)	67.40 (44.12, 99.87)
Brazil	9.50 (6.37, 13.61)	9.16 (6.14, 13.15)	9.82 (6.59, 14.05)	17378.99 (11659.84, 24904.74)	8208.23 (5501.92, 11785.25)	9170.76 (6157.92, 13119.48)
British Virgin Islands	11.98 (3.17, 35.39)	12.95 (3.35, 38.65)	11.12 (3.01, 32.51)	4.23 (1.12, 12.49)	2.14 (0.55, 6.40)	2.08 (0.56, 6.09)
Brunei Darussalam	7.38 (1.93, 22.36)	7.80 (2.00, 24.03)	6.90 (1.86, 20.45)	29.09 (7.62, 88.14)	16.40 (4.20, 50.53)	12.69 (3.43, 37.61)
Bulgaria	1.93 (0.91, 4.02)	1.97 (0.91, 4.18)	1.89 (0.91, 3.87)	117.81 (55.86, 245.77)	58.00 (26.87, 123.14)	59.82 (28.99, 122.63)
Burkina Faso	10.83 (7.10, 16.05)	10.47 (6.85, 15.55)	11.19 (7.35, 16.54)	1768.23 (1158.57, 2619.89)	844.03 (552.02, 1254.06)	924.20 (606.56, 1365.83)
Burundi	12.27 (8.03, 18.23)	12.09 (7.90, 17.99)	12.45 (8.14, 18.47)	1156.30 (756.20, 1717.8)	562.67 (367.73, 836.86)	593.64 (388.47, 880.94)
Cabo Verde	10.62 (7.07, 15.41)	10.10 (6.70, 14.73)	11.16 (7.45, 16.11)	46.05 (30.64, 66.79)	22.27 (14.77, 32.45)	23.78 (15.87, 34.33)
Cambodia	16.07 (10.68, 23.34)	15.32 (10.16, 22.34)	16.77 (11.17, 24.29)	2223.67 (1477.72, 3230.21)	1026.94 (680.87, 1497.30)	1196.74 (796.85, 1732.90)
Cameroon	8.78 (5.76, 12.98)	8.70 (5.71, 12.89)	8.85 (5.81, 13.08)	1756.33 (1152.01, 2598.46)	863.82 (566.41, 1278.96)	892.51 (585.59, 1319.50)
Canada	1.69 (0.80, 3.54)	1.80 (0.83, 3.81)	1.59 (0.77, 3.27)	593.13 (280.72, 1239.18)	312.70 (144.96, 663.20)	280.44 (135.76, 575.98)
Cayman Islands	6.61 (1.75, 19.50)	7.07 (1.83, 21.09)	6.14 (1.67, 17.89)	4.29 (1.13, 12.65)	2.31 (0.60, 6.88)	1.98 (0.54, 5.77)
Central African Republic	8.78 (5.73, 13.05)	8.33 (5.44, 12.43)	9.16 (5.99, 13.60)	297.55 (194.42, 442.62)	131.59 (85.83, 196.27)	165.97 (108.59, 246.35)
Chad	10.83 (7.08, 16.10)	10.66 (6.97, 15.85)	11.00 (7.20, 16.34)	1386.60 (906.52, 2060.67)	680.85 (445.00, 1012.48)	705.76 (461.52, 1048.19)
Chile	1.35 (0.64, 2.84)	1.44 (0.67, 3.07)	1.27 (0.61, 2.61)	235.01 (111.05, 492.53)	124.18 (57.48, 264.27)	110.82 (53.57, 228.25)
China	5.67 (3.57, 8.86)	6.09 (3.86, 9.40)	5.24 (3.27, 8.30)	72113.94 (45371.01, 112699.36)	39323.64 (24934.62, 60749.48)	32790.30 (20436.39, 51949.88)
China, Hong Kong SAR	9.34 (4.86, 16.49)	11.12 (5.75, 19.49)	7.88 (4.13, 14.05)	638.26 (331.94, 1127.5)	340.98 (176.37, 597.55)	297.28 (155.57, 529.95)
China, Macao SAR	7.73 (3.98, 13.90)	9.15 (4.67, 16.37)	6.55 (3.40, 11.83)	49.78 (25.63, 89.48)	26.87 (13.73, 48.08)	22.91 (11.90, 41.39)
China, Taiwan Province of China	12.14 (6.29, 21.59)	13.74 (7.06, 24.37)	10.58 (5.54, 18.87)	2592.49 (1342.52, 4608.83)	1450.5 (745.02, 2572.36)	1141.99 (597.50, 2036.47)
Colombia	9.19 (6.15, 13.21)	8.93 (5.97, 12.85)	9.44 (6.33, 13.55)	4150.07 (2778.42, 5965.44)	1980.64 (1324.93, 2851.95)	2169.43 (1453.49, 3113.49)
Comoros	11.98 (7.92, 17.53)	11.62 (7.68, 17.01)	12.34 (8.15, 18.05)	75.39 (49.84, 110.34)	36.70 (24.26, 53.74)	38.69 (25.58, 56.60)
Congo	8.27 (5.44, 12.19)	8.21 (5.40, 12.10)	8.33 (5.48, 12.27)	368.30 (242.21, 542.83)	182.10 (119.75, 268.53)	186.20 (122.46, 274.30)
Cook Islands	11.31 (5.83, 20.25)	13.27 (6.79, 23.69)	9.46 (4.93, 17.01)	1.36 (0.70, 2.43)	0.77 (0.40, 1.38)	0.58 (0.30, 1.05)
Costa Rica	9.97 (6.70, 14.25)	9.67 (6.49, 13.84)	10.26 (6.90, 14.64)	444.18 (298.55, 634.96)	212.32 (142.61, 303.99)	231.86 (155.94, 330.97)

Country or territory	Prevalence (%, 95% CI)			Case number (thousand, 95% CI)		
	Both	Male	Female	Both	Male	Female
Côte d'Ivoire	6.47 (4.24, 9.59)	6.56 (4.31, 9.70)	6.38 (4.18, 9.47)	1432.91 (939.22, 2121.86)	742.95 (487.52, 1098.75)	689.96 (451.70, 1023.11)
Croatia	2.38 (1.13, 4.96)	2.44 (1.13, 5.18)	2.32 (1.13, 4.76)	83.72 (39.71, 174.52)	41.36 (19.17, 87.74)	42.36 (20.54, 86.79)
Cuba	12.89 (8.69, 18.33)	13.07 (8.85, 18.49)	12.72 (8.54, 18.17)	1271.01 (856.92, 1807.04)	633.65 (429.12, 896.64)	637.36 (427.81, 910.4)
Curaçao	9.00 (2.42, 25.60)	8.83 (2.31, 25.79)	9.15 (2.53, 25.43)	15.07 (4.06, 42.88)	7.00 (1.83, 20.44)	8.07 (2.23, 22.44)
Cyprus	1.75 (0.83, 3.66)	1.86 (0.86, 3.95)	1.64 (0.79, 3.37)	20.82 (9.84, 43.64)	11.13 (5.15, 23.68)	9.69 (4.69, 19.95)
Czechia	1.92 (0.91, 4.01)	1.98 (0.92, 4.21)	1.86 (0.90, 3.82)	185.05 (87.66, 386.4)	93.98 (43.54, 199.60)	91.06 (44.12, 186.79)
Dem. People's Republic of Korea	5.30 (3.33, 8.29)	5.69 (3.60, 8.80)	4.92 (3.07, 7.80)	1217.94 (765.71, 1905.62)	643.25 (407.48, 995.25)	574.69 (358.23, 910.37)
Democratic Republic of the Congo	10.00 (6.58, 14.74)	9.89 (6.50, 14.59)	10.10 (6.65, 14.88)	7039.51 (4630.11, 10376.34)	3436.10 (2259.30, 5068.85)	3603.41 (2370.81, 5307.48)
Denmark	1.66 (0.79, 3.47)	1.75 (0.81, 3.71)	1.57 (0.76, 3.23)	87.86 (41.63, 183.29)	46.02 (21.35, 97.5)	41.84 (20.28, 85.79)
Djibouti	6.52 (4.31, 9.53)	6.18 (4.09, 9.04)	6.85 (4.54, 10.01)	60.31 (39.91, 88.14)	28.18 (18.64, 41.23)	32.13 (21.27, 46.91)
Dominica	12.86 (8.66, 18.31)	12.79 (8.62, 18.24)	12.92 (8.70, 18.39)	7.53 (5.07, 10.72)	3.75 (2.53, 5.35)	3.77 (2.54, 5.37)
Dominican Republic	8.74 (5.83, 12.62)	8.46 (5.64, 12.25)	9.01 (6.02, 12.98)	810.91 (541.06, 1171.29)	389.03 (259.26, 563.16)	421.87 (281.79, 608.14)
Ecuador	11.73 (7.83, 16.94)	11.36 (7.57, 16.43)	12.10 (8.08, 17.45)	1766.68 (1178.95, 2551.53)	849.81 (566.64, 1229.38)	916.86 (612.31, 1322.15)
Egypt	11.01 (7.27, 16.13)	11.07 (7.33, 16.16)	10.96 (7.21, 16.10)	9851.78 (6505.65, 14430.38)	4981.52 (3299.93, 7271.79)	4870.26 (3205.72, 7158.59)
El Salvador	9.26 (6.18, 13.37)	8.48 (5.64, 12.32)	9.93 (6.64, 14.30)	487.84 (325.49, 704.74)	209.31 (139.26, 303.81)	278.52 (186.23, 400.93)
Equatorial Guinea	8.36 (5.51, 12.26)	8.43 (5.56, 12.39)	8.27 (5.46, 12.12)	113.79 (75.06, 167.02)	61.54 (40.58, 90.42)	52.25 (34.49, 76.60)
Eritrea	10.32 (6.82, 15.13)	9.80 (6.46, 14.40)	10.83 (7.16, 15.83)	265.24 (175.17, 388.73)	123.23 (81.24, 181.11)	142.01 (93.93, 207.62)
Estonia	2.05 (0.97, 4.26)	2.01 (0.93, 4.27)	2.08 (1.01, 4.25)	24.79 (11.78, 51.64)	11.53 (5.34, 24.50)	13.26 (6.44, 27.14)
Eswatini	7.34 (4.82, 10.83)	7.33 (4.82, 10.81)	7.34 (4.82, 10.85)	69.70 (45.78, 102.87)	33.94 (22.33, 50.05)	35.76 (23.45, 52.83)
Ethiopia	12.02 (7.90, 17.73)	11.69 (7.68, 17.27)	12.35 (8.12, 18.18)	11191.24 (7357.11, 16506.95)	5416.81 (3558.04, 8001.76)	5774.42 (3799.07, 8505.19)
Falkland Islands (Malvinas)	1.55 (0.73, 3.25)	1.68 (0.78, 3.59)	1.42 (0.68, 2.92)	0.05 (0.02, 0.10)	0.03 (0.01, 0.05)	0.02 (0.01, 0.05)
Faroe Islands	1.59 (0.75, 3.33)	1.69 (0.79, 3.59)	1.48 (0.72, 3.05)	0.75 (0.35, 1.56)	0.41 (0.19, 0.88)	0.33 (0.16, 0.68)
Fiji	11.33 (7.54, 16.42)	11.07 (7.36, 16.07)	11.58 (7.71, 16.75)	85.49 (56.91, 123.89)	41.19 (27.39, 59.79)	44.3 (29.52, 64.09)
Finland	1.79 (0.85, 3.73)	1.85 (0.86, 3.93)	1.73 (0.84, 3.53)	89.88 (42.63, 187.24)	46.12 (21.40, 97.65)	43.76 (21.23, 89.59)
France	1.82 (0.86, 3.80)	1.90 (0.88, 4.03)	1.75 (0.85, 3.58)	1063.83 (504.56, 2216.94)	538.33 (249.75, 1140.35)	525.5 (254.82, 1076.59)
French Guiana	6.96 (1.83, 20.93)	7.58 (1.95, 23.11)	6.38 (1.72, 18.95)	16.64 (4.38, 50.09)	8.64 (2.23, 26.34)	8.00 (2.16, 23.75)
French Polynesia	10.58 (2.81, 30.95)	11.33 (2.95, 33.49)	9.82 (2.67, 28.35)	26.07 (6.93, 76.26)	14.10 (3.67, 41.69)	11.97 (3.26, 34.57)

Country or territory	Prevalence (%, 95% CI)			Case number (thousand, 95% CI)		
	Both	Male	Female	Both	Male	Female
Gabon	6.22 (4.10, 9.12)	6.23 (4.11, 9.15)	6.2 (4.09, 9.09)	114.69 (75.07, 168.20)	58.61 (38.68, 86.02)	56.07 (37.02, 82.18)
Gambia	7.59 (4.99, 11.20)	7.39 (4.86, 10.92)	7.79 (5.13, 11.48)	147.64 (97.06, 217.75)	71.17 (46.75, 105.10)	76.48 (50.31, 112.65)
Georgia	5.71 (3.59, 8.92)	5.80 (3.68, 8.96)	5.62 (3.51, 8.87)	185.93 (116.98, 290.50)	86.89 (55.09, 134.22)	99.04 (61.89, 156.28)
Germany	2.06 (0.98, 4.29)	2.14 (0.99, 4.53)	1.98 (0.96, 4.05)	1558.08 (739.20, 3244.36)	799.75 (371.28, 1692.19)	758.33 (367.92, 1552.16)
Ghana	9.6 (6.33, 14.10)	9.48 (6.25, 13.94)	9.72 (6.41, 14.25)	2438.69 (1608.06, 3581.32)	1197.44 (789.19, 1760.4)	1241.24 (818.87, 1820.92)
Gibraltar	1.15 (0.55, 2.41)	1.23 (0.57, 2.61)	1.08 (0.52, 2.21)	0.39 (0.18, 0.81)	0.21 (0.10, 0.44)	0.18 (0.09, 0.37)
Greece	1.92 (0.91, 4.00)	2.00 (0.93, 4.23)	1.85 (0.9, 3.78)	177.15 (84.07, 368.92)	89.29 (41.45, 189.01)	87.86 (42.62, 179.91)
Greenland	1.25 (0.59, 2.64)	1.39 (0.64, 2.97)	1.10 (0.53, 2.27)	0.60 (0.28, 1.26)	0.35 (0.16, 0.75)	0.25 (0.12, 0.51)
Grenada	18.13 (12.19, 25.91)	17.7 (11.89, 25.34)	18.56 (12.49, 26.48)	18.47 (12.42, 26.39)	9.06 (6.08, 12.97)	9.41 (6.33, 13.43)
Guadeloupe	9.25 (2.52, 25.55)	9.69 (2.57, 27.02)	8.90 (2.47, 24.35)	30.76 (8.36, 84.92)	14.48 (3.84, 40.38)	16.28 (4.52, 44.54)
Guam	7.24 (1.93, 20.92)	7.57 (1.97, 22.20)	6.90 (1.89, 19.61)	9.86 (2.63, 28.50)	5.21 (1.36, 15.29)	4.65 (1.27, 13.21)
Guatemala	10.10 (6.67, 14.8)	9.79 (6.46, 14.37)	10.41 (6.88, 15.23)	1437.08 (949.16, 2106.09)	686.17 (452.74, 1007.45)	750.92 (496.41, 1098.64)
Guernsey	2.35 (1.11, 4.89)	2.47 (1.15, 5.24)	2.22 (1.08, 4.55)	1.35 (0.64, 2.82)	0.71 (0.33, 1.49)	0.65 (0.31, 1.32)
Guinea	10.16 (6.68, 14.97)	9.61 (6.31, 14.19)	10.69 (7.04, 15.71)	1039.56 (684.04, 1531.48)	480.94 (315.91, 710.52)	558.62 (368.13, 820.96)
Guinea-Bissau	9.75 (6.40, 14.4)	9.28 (6.08, 13.76)	10.2 (6.71, 15.01)	153.29 (100.65, 226.46)	71.41 (46.77, 105.91)	81.87 (53.88, 120.54)
Guyana	12.13 (3.19, 36.69)	12.84 (3.29, 39.6)	11.47 (3.10, 33.98)	80.18 (21.10, 242.47)	40.93 (10.49, 126.22)	39.25 (10.61, 116.26)
Haiti	10.20 (6.75, 14.93)	9.97 (6.59, 14.62)	10.43 (6.90, 15.24)	935.81 (618.65, 1369.43)	450.5 (297.51, 660.47)	485.31 (321.14, 708.97)
Holy See	1.81 (0.87, 3.73)	1.83 (0.85, 3.83)	1.80 (0.88, 3.63)	0.01 (0.00, 0.02)	0.00 (0.00, 0.01)	0.00 (0.00, 0.01)
Honduras	9.55 (6.30, 14.01)	9.32 (6.14, 13.70)	9.78 (6.46, 14.33)	803.22 (529.98, 1178.55)	393.05 (259.1, 577.68)	410.17 (270.88, 600.87)
Hungary	1.96 (0.93, 4.08)	1.97 (0.91, 4.20)	1.94 (0.94, 3.98)	169.93 (80.58, 354.62)	81.96 (37.95, 174.19)	87.97 (42.63, 180.42)
Iceland	1.33 (0.63, 2.79)	1.41 (0.66, 3.00)	1.24 (0.60, 2.56)	4.52 (2.14, 9.47)	2.46 (1.14, 5.23)	2.06 (1.00, 4.24)
India	13.89 (9.22, 20.22)	14.27 (9.50, 20.68)	13.49 (8.91, 19.73)	166998.54 (110783.44, 243076.61)	88357.81 (58834.31, 128074.28)	78640.73 (51949.13, 115002.33)
Indonesia	11.23 (7.49, 16.23)	10.68 (7.11, 15.46)	11.78 (7.87, 17.00)	26411.03 (17612.53, 38169.28)	12563.77 (8367.94, 18198.02)	13847.26 (9244.59, 19971.26)
Iran (Islamic Republic of)	4.19 (2.67, 6.43)	4.21 (2.70, 6.42)	4.16 (2.63, 6.45)	3213.40 (2046.97, 4935.61)	1641.46 (1052.26, 2499.08)	1571.94 (994.71, 2436.53)
Iraq	3.37 (2.13, 5.23)	3.34 (2.12, 5.15)	3.39 (2.14, 5.30)	1140.09 (721.21, 1769.64)	563.47 (358.20, 868.73)	576.62 (363.01, 900.91)
Ireland	1.95 (0.92, 4.09)	2.09 (0.97, 4.43)	1.83 (0.88, 3.76)	88.76 (41.97, 185.77)	46.79 (21.67, 99.41)	41.97 (20.30, 86.36)

Country or territory	Prevalence (%, 95% CI)			Case number (thousand, 95% CI)		
	Both	Male	Female	Both	Male	Female
Isle of Man	2.40 (1.14, 5.00)	2.56 (1.19, 5.41)	2.24 (1.09, 4.60)	1.82 (0.86, 3.79)	0.96 (0.45, 2.03)	0.86 (0.41, 1.76)
Israel	4.00 (1.95, 7.87)	4.46 (2.14, 8.86)	3.56 (1.77, 6.91)	298.48 (145.63, 587.14)	164.59 (78.94, 327.01)	133.89 (66.69, 260.13)
Italy	2.20 (1.05, 4.59)	2.30 (1.07, 4.87)	2.11 (1.02, 4.32)	1193.84 (566.41, 2486.01)	610.22 (283.27, 1291.29)	583.62 (283.14, 1194.72)
Jamaica	12.92 (8.62, 18.65)	12.46 (8.31, 18.04)	13.36 (8.93, 19.24)	322.17 (215.02, 465.13)	153.23 (102.13, 221.75)	168.94 (112.88, 243.37)
Japan	1.79 (0.85, 3.71)	1.86 (0.87, 3.93)	1.72 (0.83, 3.50)	2012.77 (956.79, 4178.8)	1030.08 (478.88, 2174.52)	982.69 (477.91, 2004.29)
Jersey	1.41 (0.67, 2.94)	1.5 (0.70, 3.18)	1.32 (0.64, 2.71)	1.31 (0.62, 2.74)	0.69 (0.32, 1.46)	0.62 (0.30, 1.28)
Jordan	2.44 (1.55, 3.77)	2.5 (1.60, 3.84)	2.37 (1.50, 3.70)	221.66 (140.61, 342.63)	117.56 (75.01, 180.24)	104.10 (65.60, 162.39)
Kazakhstan	4.21 (2.64, 6.62)	4.35 (2.74, 6.76)	4.08 (2.54, 6.49)	680.65 (426.56, 1070.26)	338.13 (213.44, 526.08)	342.52 (213.11, 544.18)
Kenya	11.5 (7.55, 17.00)	11.39 (7.47, 16.85)	11.61 (7.62, 17.14)	4765.21 (3127.29, 7044.66)	2337.56 (1533.26, 3459.23)	2427.65 (1594.04, 3585.42)
Kiribati	9.86 (6.52, 14.42)	9.39 (6.20, 13.77)	10.29 (6.82, 15.01)	9.95 (6.58, 14.54)	4.51 (2.98, 6.62)	5.43 (3.60, 7.92)
Kosovo (under UNSC res. 1244)	4.70 (2.95, 7.38)	5.00 (3.16, 7.76)	4.42 (2.75, 7.02)	69.54 (43.63, 109.15)	36.05 (22.80, 55.93)	33.49 (20.83, 53.23)
Kuwait	4.67 (2.34, 8.74)	5.23 (2.60, 9.78)	3.75 (1.91, 7.00)	198.89 (99.67, 372.11)	139.15 (69.21, 260.51)	59.74 (30.46, 111.6)
Kyrgyzstan	4.52 (2.83, 7.14)	4.8 (3.03, 7.49)	4.26 (2.64, 6.79)	248.14 (155.19, 391.46)	128.86 (81.20, 201.05)	119.27 (73.98, 190.40)
Lao People's Democratic Republic	13.39 (8.85, 19.58)	13.17 (8.71, 19.29)	13.60 (8.99, 19.87)	813.01 (537.56, 1189.4)	400.53 (264.72, 586.54)	412.48 (272.85, 602.86)
Latvia	2.13 (1.01, 4.43)	2.07 (0.96, 4.40)	2.18 (1.06, 4.46)	35.59 (16.92, 74.08)	15.9 (7.37, 33.78)	19.69 (9.55, 40.30)
Lebanon	3.54 (2.26, 5.41)	3.53 (2.27, 5.34)	3.55 (2.26, 5.47)	169.29 (108.17, 258.7)	81.17 (52.20, 122.94)	88.12 (55.97, 135.76)
Lesotho	11.16 (7.31, 16.52)	11.05 (7.24, 16.36)	11.27 (7.38, 16.68)	197.07 (129.16, 291.81)	94.07 (61.68, 139.31)	103.01 (67.48, 152.50)
Liberia	9.50 (6.25, 13.99)	9.37 (6.16, 13.81)	9.63 (6.33, 14.17)	379.04 (249.37, 558.44)	185.54 (122.03, 273.58)	193.5 (127.34, 284.86)
Libya	6.23 (4.12, 9.12)	6.14 (4.07, 8.95)	6.32 (4.16, 9.30)	371.38 (245.33, 543.64)	185.81 (123.23, 270.75)	185.57 (122.10, 272.88)
Liechtenstein	3.16 (1.49, 6.59)	3.35 (1.55, 7.11)	2.96 (1.43, 6.09)	1.12 (0.53, 2.35)	0.59 (0.27, 1.25)	0.53 (0.26, 1.09)
Lithuania	2.08 (0.99, 4.33)	2.02 (0.94, 4.30)	2.12 (1.03, 4.35)	52.96 (25.16, 110.34)	24.24 (11.23, 51.51)	28.72 (13.93, 58.83)
Luxembourg	1.45 (0.69, 3.03)	1.53 (0.71, 3.25)	1.37 (0.66, 2.81)	8.55 (4.04, 17.91)	4.55 (2.11, 9.68)	4.00 (1.94, 8.23)
Madagascar	10.71 (7.04, 15.78)	10.6 (6.98, 15.63)	10.81 (7.11, 15.92)	2415.57 (1589.16, 3559.09)	1195.14 (786.23, 1761.64)	1220.43 (802.93, 1797.45)
Malawi	11.69 (7.64, 17.36)	11.34 (7.40, 16.88)	12.01 (7.87, 17.81)	1770.79 (1158.21, 2630.47)	829.63 (541.75, 1235.41)	941.17 (616.46, 1395.06)
Malaysia	7.99 (5.32, 11.57)	7.72 (5.14, 11.2)	8.29 (5.53, 11.98)	2398.19 (1597.39, 3472.54)	1219.11 (811.44, 1767.93)	1179.08 (785.95, 1704.61)
Maldives	11.33 (7.47, 16.64)	10.75 (7.07, 15.85)	12.34 (8.17, 18.01)	52.04 (34.32, 76.42)	31.33 (20.60, 46.19)	20.72 (13.72, 30.23)
Mali	8.68 (5.68, 12.88)	8.55 (5.59, 12.7)	8.81 (5.76, 13.07)	1382.49 (904.35, 2053.13)	686.86 (449.22, 1020.58)	695.63 (455.13, 1032.54)

Country or territory	Prevalence (%, 95% CI)			Case number (thousand, 95% CI)		
	Both	Male	Female	Both	Male	Female
Malta	1.44 (0.68, 3.01)	1.49 (0.69, 3.17)	1.39 (0.67, 2.85)	6.96 (3.29, 14.54)	3.75 (1.74, 7.97)	3.21 (1.55, 6.57)
Marshall Islands	7.79 (5.15, 11.37)	7.72 (5.11, 11.27)	7.86 (5.20, 11.48)	2.35 (1.55, 3.43)	1.19 (0.79, 1.74)	1.16 (0.76, 1.69)
Martinique	11.1 (3.02, 30.45)	11.65 (3.1, 32.25)	10.64 (2.96, 28.98)	34.25 (9.33, 94.00)	16.22 (4.32, 44.91)	18.03 (5.01, 49.09)
Mauritania	7.59 (4.98, 11.23)	7.63 (5.02, 11.23)	7.55 (4.93, 11.23)	265.73 (174.23, 393.24)	129.10 (84.99, 190.13)	136.63 (89.24, 203.11)
Mauritius	17.5 (11.73, 25.08)	17.86 (12.02, 25.49)	17.13 (11.44, 24.67)	200.81 (134.63, 287.85)	102.68 (69.11, 146.57)	98.13 (65.51, 141.28)
Mayotte	8.24 (2.12, 26.38)	9.54 (2.41, 30.73)	7.10 (1.88, 22.55)	18.16 (4.68, 58.11)	9.83 (2.48, 31.66)	8.33 (2.20, 26.45)
Mexico	8.40 (5.59, 12.19)	8.55 (5.71, 12.34)	8.27 (5.48, 12.04)	9111.93 (6060.38, 13213.19)	4453.38 (2974.61, 6427.57)	4658.55 (3085.77, 6785.62)
Micronesia (Fed. States of)	14.75 (9.8, 21.45)	14.03 (9.30, 20.46)	15.45 (10.28, 22.4)	13.06 (8.68, 18.99)	6.11 (4.05, 8.92)	6.95 (4.63, 10.07)
Monaco	1.60 (0.76, 3.32)	1.71 (0.80, 3.59)	1.50 (0.73, 3.05)	0.53 (0.25, 1.09)	0.28 (0.13, 0.58)	0.25 (0.12, 0.51)
Mongolia	3.50 (2.19, 5.53)	3.70 (2.33, 5.78)	3.31 (2.05, 5.29)	93.44 (58.40, 147.61)	48.82 (30.73, 76.31)	44.62 (27.67, 71.29)
Montenegro	5.22 (3.29, 8.14)	5.46 (3.47, 8.41)	5.01 (3.13, 7.90)	28.96 (18.25, 45.16)	14.47 (9.19, 22.29)	14.50 (9.06, 22.87)
Montserrat	20.90 (5.63, 58.63)	22.54 (5.96, 63.56)	19.22 (5.30, 53.59)	0.82 (0.22, 2.30)	0.45 (0.12, 1.26)	0.37 (0.10, 1.04)
Morocco	5.22 (3.33, 8.01)	5.3 (3.40, 8.06)	5.14 (3.25, 7.95)	1630.03 (1038.87, 2501.13)	832.45 (533.84, 1266.44)	797.57 (505.03, 1234.69)
Mozambique	9.78 (6.40, 14.49)	9.09 (5.93, 13.56)	10.40 (6.83, 15.35)	2247.45 (1472.15, 3331.41)	996.33 (650.02, 1485.41)	1251.11 (822.13, 1845.99)
Myanmar	15.49 (10.29, 22.51)	15.73 (10.48, 22.78)	15.26 (10.10, 22.25)	7003.67 (4650.10, 10179.1)	3519.33 (2344.34, 5097.42)	3484.34 (2305.76, 5081.68)
Namibia	9.21 (6.05, 13.61)	9.24 (6.07, 13.63)	9.19 (6.02, 13.58)	200.59 (131.70, 296.23)	97.55 (64.14, 143.89)	103.04 (67.56, 152.34)
Nauru	3.98 (1.03, 12.72)	4.26 (1.07, 13.94)	3.69 (0.98, 11.46)	0.35 (0.09, 1.13)	0.19 (0.05, 0.63)	0.16 (0.04, 0.50)
Nepal	14.69 (9.72, 21.44)	15.26 (10.15, 22.14)	14.18 (9.34, 20.82)	3532.71 (2338.70, 5157.52)	1734.51 (1154.11, 2517.07)	1798.20 (1184.59, 2640.45)
Netherlands	1.57 (0.74, 3.27)	1.66 (0.77, 3.52)	1.47 (0.71, 3.02)	253.04 (119.81, 528.24)	133.18 (61.76, 282.28)	119.86 (58.05, 245.96)
New Caledonia	10.89 (5.59, 19.67)	12.63 (6.42, 22.79)	9.21 (4.78, 16.65)	26.98 (13.85, 48.72)	15.37 (7.82, 27.73)	11.61 (6.03, 20.99)
New Zealand	1.55 (0.73, 3.24)	1.64 (0.76, 3.49)	1.46 (0.71, 3.01)	70.19 (33.21, 146.76)	36.82 (17.06, 78.14)	33.38 (16.15, 68.62)
Nicaragua	10.27 (6.81, 14.98)	9.88 (6.54, 14.45)	10.65 (7.07, 15.49)	564.42 (374.00, 823.27)	264.99 (175.31, 387.56)	299.43 (198.69, 435.72)
Niger	11.46 (7.51, 16.97)	11.26 (7.38, 16.68)	11.67 (7.65, 17.27)	1995.56 (1308.27, 2954.69)	995.23 (652.36, 1474.28)	1000.33 (655.91, 1480.41)
Nigeria	8.83 (5.80, 13.04)	8.76 (5.75, 12.93)	8.91 (5.85, 13.15)	14355.89 (9430.83, 21194.23)	7190.77 (4724.29, 10618.18)	7165.13 (4706.54, 10576.05)
Niue	14.10 (3.80, 39.95)	15.2 (4.00, 43.5)	13.11 (3.61, 36.72)	0.22 (0.06, 0.62)	0.11 (0.03, 0.32)	0.11 (0.03, 0.30)
North Macedonia	6.79 (4.28, 10.59)	7.29 (4.63, 11.23)	6.33 (3.95, 10.00)	110.19 (69.43, 171.80)	57.21 (36.33, 88.13)	52.99 (33.09, 83.68)

Country or territory	Prevalence (%, 95% CI)			Case number (thousand, 95% CI)		
	Both	Male	Female	Both	Male	Female
Northern Mariana Islands	7.58 (2.01, 22.10)	8.14 (2.12, 23.91)	6.96 (1.89, 20.08)	2.94 (0.78, 8.57)	1.66 (0.43, 4.88)	1.28 (0.35, 3.69)
Norway	1.68 (0.79, 3.50)	1.78 (0.82, 3.77)	1.57 (0.76, 3.22)	81.98 (38.79, 171.32)	43.91 (20.35, 93.15)	38.07 (18.44, 78.17)
Oman	5.58 (2.77, 10.58)	6.21 (3.06, 11.84)	4.42 (2.25, 8.28)	231.41 (115.09, 438.82)	166.29 (81.92, 316.84)	65.12 (33.18, 121.98)
Pakistan	5.49 (3.48, 8.49)	5.58 (3.56, 8.56)	5.40 (3.40, 8.41)	10173.19 (6450.48, 15734.71)	5242.88 (3343.01, 8046.20)	4930.31 (3107.47, 7688.51)
Palau	9.00 (2.39, 26.16)	9.30 (2.41, 27.47)	8.64 (2.36, 24.60)	1.41 (0.37, 4.09)	0.79 (0.20, 2.32)	0.62 (0.17, 1.76)
Panama	9.06 (2.40, 26.89)	9.58 (2.48, 28.93)	8.54 (2.32, 24.86)	335.02 (88.69, 994.23)	176.56 (45.60, 532.91)	158.45 (43.09, 461.32)
Papua New Guinea	14.43 (9.51, 21.22)	14.45 (9.53, 21.22)	14.42 (9.48, 21.23)	1152.54 (759.27, 1694.6)	592.94 (391.12, 870.59)	559.60 (368.15, 824.01)
Paraguay	10.58 (7.01, 15.43)	10.79 (7.18, 15.68)	10.38 (6.85, 15.18)	580.51 (384.72, 846.17)	295.63 (196.58, 429.37)	284.88 (188.15, 416.8)
Peru	10.25 (6.85, 14.76)	10.01 (6.70, 14.43)	10.48 (7.01, 15.08)	2903.95 (1942.03, 4181.48)	1406.61 (940.57, 2026.81)	1497.34 (1001.46, 2154.67)
Philippines	12.15 (8.06, 17.72)	11.81 (7.81, 17.25)	12.50 (8.30, 18.17)	11397.50 (7555.49, 16612.80)	5494.05 (3636.32, 8028.89)	5903.45 (3919.17, 8583.91)
Poland	2.14 (1.01, 4.47)	2.19 (1.01, 4.66)	2.10 (1.02, 4.30)	743.75 (352.36, 1553.56)	367.84 (170.3, 781.99)	375.91 (182.06, 771.58)
Portugal	2.29 (1.09, 4.77)	2.38 (1.10, 5.03)	2.21 (1.07, 4.52)	216.09 (102.57, 449.92)	106.75 (49.54, 225.99)	109.34 (53.03, 223.93)
Puerto Rico	9.76 (2.65, 27.05)	10.01 (2.65, 28.13)	9.55 (2.65, 26.08)	290.39 (78.89, 804.49)	139.80 (37.03, 393.01)	150.59 (41.86, 411.48)
Qatar	5.07 (2.51, 9.64)	5.58 (2.75, 10.64)	3.60 (1.82, 6.81)	135.01 (66.82, 256.92)	109.95 (54.12, 209.54)	25.06 (12.70, 47.37)
Republic of Korea	1.76 (0.83, 3.69)	1.84 (0.85, 3.91)	1.69 (0.82, 3.47)	846.89 (400.53, 1771.81)	441.71 (204.50, 938.83)	405.18 (196.03, 832.99)
Republic of Moldova	7.17 (4.51, 11.21)	7.32 (4.64, 11.31)	7.04 (4.40, 11.13)	190.15 (119.53, 297.46)	87.95 (55.74, 135.95)	102.20 (63.79, 161.50)
Réunion	20.68 (10.69, 36.90)	24.09 (12.35, 42.88)	17.63 (9.21, 31.58)	155.15 (80.20, 276.89)	85.16 (43.66, 151.56)	69.98 (36.54, 125.33)
Romania	2.33 (1.10, 4.86)	2.37 (1.10, 5.03)	2.30 (1.11, 4.71)	396.86 (188.08, 828.66)	194.34 (89.99, 413.05)	202.51 (98.09, 415.61)
Russian Federation	1.69 (0.80, 3.53)	1.69 (0.78, 3.60)	1.68 (0.81, 3.46)	2161.64 (1024.67, 4517.22)	996.20 (460.71, 2122.17)	1165.43 (563.96, 2395.05)
Rwanda	13.48 (8.88, 19.83)	12.92 (8.49, 19.06)	14.01 (9.25, 20.55)	1389.25 (915.15, 2043.35)	641.44 (421.69, 946.45)	747.81 (493.46, 1096.90)
Saint Barthélemy	18.94 (5.11, 53.40)	19.84 (5.22, 56.64)	18.17 (5.01, 50.59)	1.81 (0.49, 5.11)	0.88 (0.23, 2.52)	0.93 (0.26, 2.59)
Saint Helena	22.57 (11.79, 39.47)	26.26 (13.65, 45.58)	18.86 (9.92, 33.31)	1.08 (0.57, 1.89)	0.63 (0.33, 1.10)	0.45 (0.24, 0.80)
Saint Kitts and Nevis	14.42 (3.84, 42.08)	15.48 (4.02, 45.73)	13.45 (3.66, 38.76)	5.88 (1.56, 17.17)	3.01 (0.78, 8.89)	2.87 (0.78, 8.27)
Saint Lucia	20.78 (13.91, 29.85)	20.06 (13.42, 28.88)	21.47 (14.39, 30.79)	32.88 (22.02, 47.23)	15.65 (10.47, 22.53)	17.23 (11.55, 24.70)
Saint Martin (French part)	18.98 (5.12, 53.42)	19.96 (5.26, 56.82)	18.14 (5.01, 50.49)	4.47 (1.21, 12.57)	2.17 (0.57, 6.19)	2.29 (0.63, 6.38)
Saint Pierre and Miquelon	1.56 (0.74, 3.25)	1.65 (0.76, 3.50)	1.46 (0.71, 3.01)	0.08 (0.04, 0.17)	0.04 (0.02, 0.09)	0.04 (0.02, 0.08)

Country or territory	Prevalence (%, 95% CI)			Case number (thousand, 95% CI)		
	Both	Male	Female	Both	Male	Female
Saint Vincent and the Grenadines	15.74 (10.59, 22.47)	15.60 (10.50, 22.27)	15.89 (10.69, 22.67)	13.69 (9.21, 19.54)	6.93 (4.67, 9.90)	6.76 (4.55, 9.64)
Samoa	16.09 (10.71, 23.31)	15.90 (10.59, 23.05)	16.28 (10.84, 23.58)	25.64 (17.07, 37.15)	12.65 (8.42, 18.34)	12.99 (8.65, 18.81)
San Marino	1.40 (0.66, 2.92)	1.49 (0.69, 3.16)	1.31 (0.63, 2.69)	0.43 (0.20, 0.90)	0.23 (0.10, 0.48)	0.20 (0.10, 0.42)
Sao Tome and Principe	7.73 (5.10, 11.34)	7.71 (5.08, 11.32)	7.74 (5.11, 11.35)	13.19 (8.70, 19.35)	6.51 (4.30, 9.57)	6.67 (4.40, 9.79)
Saudi Arabia	5.67 (2.83, 10.68)	6.44 (3.19, 12.16)	4.38 (2.23, 8.22)	1580.23 (789.05, 2976.97)	1122.84 (556.24, 2119.02)	457.39 (232.81, 857.95)
Senegal	9.09 (5.98, 13.38)	8.92 (5.88, 13.13)	9.25 (6.09, 13.63)	1209.43 (796.14, 1780.64)	599.88 (395.16, 882.71)	609.55 (400.98, 897.94)
Serbia	6.70 (4.23, 10.41)	7.08 (4.50, 10.87)	6.36 (3.98, 10.01)	408.08 (257.53, 634.61)	203.89 (129.75, 313.16)	204.19 (127.78, 321.45)
Seychelles	10.82 (2.85, 32.26)	11.04 (2.84, 33.65)	10.53 (2.87, 30.50)	11.92 (3.14, 35.55)	6.8 (1.75, 20.72)	5.12 (1.39, 14.83)
Sierra Leone	9.82 (6.46, 14.48)	9.63 (6.33, 14.21)	10.01 (6.58, 14.74)	610.79 (401.59, 900.62)	297.51 (195.50, 439.20)	313.28 (206.10, 461.42)
Singapore	7.02 (1.87, 20.46)	7.26 (1.89, 21.54)	6.76 (1.85, 19.29)	372.57 (99.22, 1085.82)	199.91 (51.96, 592.75)	172.65 (47.26, 493.07)
Sint Maarten (Dutch part)	7.64 (2.04, 21.83)	8.28 (2.17, 23.80)	7.04 (1.92, 20.01)	2.96 (0.79, 8.47)	1.55 (0.41, 4.45)	1.41 (0.39, 4.02)
Slovakia	2.24 (1.06, 4.68)	2.30 (1.06, 4.89)	2.18 (1.06, 4.49)	109.95 (52.04, 229.95)	54.90 (25.40, 116.83)	55.04 (26.64, 113.12)
Slovenia	2.40 (1.14, 5.01)	2.48 (1.15, 5.26)	2.31 (1.12, 4.75)	45.46 (21.53, 94.91)	23.69 (10.98, 50.28)	21.77 (10.55, 44.64)
Solomon Islands	12.84 (8.47, 18.88)	12.85 (8.48, 18.88)	12.83 (8.45, 18.87)	76.32 (50.30, 112.15)	38.94 (25.68, 57.18)	37.38 (24.61, 54.97)
Somalia	8.31 (5.44, 12.30)	7.92 (5.19, 11.75)	8.68 (5.70, 12.84)	1002.89 (657.39, 1485.19)	476.12 (311.87, 705.99)	526.77 (345.52, 779.20)
South Africa	4.90 (3.12, 7.55)	4.92 (3.14, 7.53)	4.89 (3.09, 7.58)	2546.44 (1618.87, 3922.84)	1235.19 (789.04, 1890.66)	1311.25 (829.83, 2032.18)
South Sudan	13.04 (8.57, 19.24)	12.55 (8.24, 18.55)	13.51 (8.89, 19.88)	1114.24 (732.34, 1643.42)	522.39 (342.92, 772.10)	591.85 (389.42, 871.32)
Spain	1.85 (0.88, 3.87)	1.94 (0.90, 4.11)	1.77 (0.86, 3.63)	803.94 (380.92, 1677.79)	413.43 (191.68, 876.84)	390.5 (189.23, 800.94)
Sri Lanka	20.85 (14.02, 29.79)	19.91 (13.36, 28.53)	21.71 (14.62, 30.95)	4081.97 (2744.32, 5832.47)	1870.42 (1255.50, 2680.20)	2211.55 (1488.82, 3152.27)
State of Palestine	3.55 (2.25, 5.50)	3.68 (2.35, 5.66)	3.42 (2.15, 5.34)	141.04 (89.34, 218.49)	72.03 (45.88, 110.72)	69.01 (43.46, 107.77)
Sudan	10.29 (6.77, 15.17)	10.08 (6.64, 14.84)	10.49 (6.89, 15.49)	3673.77 (2416.22, 5415.26)	1768.83 (1164.89, 2603.75)	1904.94 (1251.33, 2811.51)
Suriname	11.17 (7.46, 16.11)	10.82 (7.21, 15.64)	11.53 (7.71, 16.58)	58.08 (38.79, 83.75)	28.03 (18.69, 40.54)	30.05 (20.1, 43.21)
Sweden	1.64 (0.78, 3.42)	1.73 (0.80, 3.66)	1.55 (0.75, 3.17)	151.79 (71.92, 316.59)	80.67 (37.44, 170.85)	71.12 (34.48, 145.74)
Switzerland	1.98 (0.94, 4.13)	2.08 (0.96, 4.41)	1.88 (0.91, 3.86)	156.21 (73.99, 326.06)	81.58 (37.83, 172.96)	74.63 (36.16, 153.10)
Syrian Arab Republic	4.46 (2.83, 6.90)	4.44 (2.83, 6.82)	4.48 (2.83, 6.98)	859.22 (544.85, 1328.64)	425.67 (271.43, 653.19)	433.55 (273.41, 675.45)
Tajikistan	4.26 (2.66, 6.73)	4.63 (2.91, 7.24)	3.90 (2.42, 6.25)	329.09 (205.44, 520.76)	173.69 (109.24, 271.81)	155.39 (96.19, 248.94)

Country or territory	Prevalence (%, 95% CI)			Case number (thousand, 95% CI)		
	Both	Male	Female	Both	Male	Female
Thailand	17.71 (11.96, 25.15)	17.05 (11.50, 24.27)	18.33 (12.39, 25.99)	11435.81 (7720.06, 16241.95)	5343.62 (3603.70, 7605.66)	6092.18 (4116.36, 8636.29)
Timor-Leste	12.84 (8.51, 18.74)	12.77 (8.46, 18.64)	12.92 (8.56, 18.85)	136.36 (90.34, 199.02)	68.15 (45.14, 99.52)	68.21 (45.19, 99.50)
Togo	10.60 (6.98, 15.62)	10.56 (6.94, 15.56)	10.65 (7.01, 15.69)	713.64 (469.46, 1051.44)	357.08 (234.92, 526.23)	356.57 (234.55, 525.21)
Tokelau	15.46 (4.09, 46.03)	15.73 (4.05, 48.08)	15.19 (4.14, 44.03)	0.31 (0.08, 0.91)	0.15 (0.04, 0.47)	0.15 (0.04, 0.44)
Tonga	14.75 (9.79, 21.44)	15.22 (10.15, 21.99)	14.35 (9.49, 20.97)	11.80 (7.84, 17.16)	5.62 (3.75, 8.12)	6.18 (4.09, 9.04)
Trinidad and Tobago	12.22 (3.25, 35.53)	12.75 (3.31, 37.71)	11.69 (3.20, 33.4)	161.90 (43.13, 470.83)	83.31 (21.64, 246.38)	78.59 (21.48, 224.45)
Tunisia	5.18 (3.31, 7.94)	5.23 (3.36, 7.94)	5.14 (3.26, 7.93)	529.65 (338.04, 810.9)	262.65 (168.71, 398.56)	267.01 (169.33, 412.34)
Türkiye	3.81 (2.39, 5.98)	4.04 (2.55, 6.26)	3.58 (2.23, 5.69)	2844.31 (1785.28, 4462.49)	1502.09 (950.07, 2330.01)	1342.22 (835.21, 2132.49)
Turkmenistan	3.62 (2.26, 5.73)	3.74 (2.35, 5.85)	3.51 (2.17, 5.61)	207.50 (129.52, 328.41)	103.77 (65.23, 162.55)	103.73 (64.29, 165.86)
Turks and Caicos Islands	8.70 (4.46, 15.73)	10.12 (5.14, 18.27)	7.27 (3.78, 13.16)	3.56 (1.83, 6.44)	2.08 (1.06, 3.75)	1.49 (0.77, 2.69)
Tuvalu	10.49 (7.00, 15.13)	9.79 (6.52, 14.20)	11.21 (7.51, 16.09)	0.80 (0.53, 1.15)	0.38 (0.25, 0.55)	0.42 (0.28, 0.60)
Uganda	10.77 (7.03, 16.05)	10.52 (6.86, 15.71)	11.01 (7.20, 16.38)	3625.79 (2366.40, 5401.82)	1742.85 (1135.82, 2602.27)	1882.94 (1230.57, 2799.55)
Ukraine	5.64 (3.55, 8.79)	5.7 (3.62, 8.80)	5.58 (3.49, 8.79)	1930.56 (1215.79, 3012.23)	903.72 (573.48, 1394.55)	1026.83 (642.31, 1617.68)
United Arab Emirates	5.43 (2.70, 10.32)	6.1 (3.01, 11.62)	4.15 (2.10, 7.84)	513.68 (255.02, 975.85)	379.01 (186.75, 721.51)	134.67 (68.27, 254.34)
United Kingdom	1.69 (0.80, 3.53)	1.78 (0.83, 3.77)	1.61 (0.78, 3.29)	1020.86 (483.68, 2130.35)	527.97 (244.90, 1119.00)	492.89 (238.78, 1011.34)
United Republic of Tanzania	10.55 (6.93, 15.61)	10.21 (6.69, 15.14)	10.89 (7.16, 16.06)	4884.64 (3205.43, 7222.61)	2322.56 (1521.40, 3443.65)	2562.08 (1684.03, 3778.96)
United States of America	1.60 (0.76, 3.35)	1.69 (0.78, 3.59)	1.52 (0.73, 3.12)	4844.38 (2291.44, 10130.72)	2564.61 (1188.13, 5445.86)	2279.78 (1103.31, 4684.85)
United States Virgin Islands	9.51 (2.58, 26.34)	9.78 (2.59, 27.48)	9.27 (2.57, 25.32)	7.22 (1.96, 20.00)	3.52 (0.93, 9.89)	3.70 (1.03, 10.11)
Uruguay	4.37 (2.14, 8.56)	4.70 (2.26, 9.31)	4.07 (2.03, 7.86)	128.98 (63.14, 252.56)	67.21 (32.28, 133.14)	61.78 (30.86, 119.42)
Uzbekistan	3.93 (2.46, 6.19)	4.15 (2.62, 6.48)	3.70 (2.30, 5.90)	1088.76 (681.21, 1716.83)	576.57 (363.32, 899.7)	512.19 (317.89, 817.14)
Vanuatu	13.29 (8.79, 19.46)	13.44 (8.9, 19.65)	13.15 (8.68, 19.28)	31.08 (20.54, 45.51)	15.75 (10.42, 23.02)	15.33 (10.12, 22.49)
Venezuela (Bolivarian Republic of)	7.84 (5.25, 11.25)	7.51 (5.02, 10.80)	8.16 (5.48, 11.69)	1851.43 (1240.68, 2657.65)	870.16 (582.31, 1252.22)	981.27 (658.37, 1405.43)
Viet Nam	17.02 (11.39, 24.49)	16.21 (10.81, 23.41)	17.79 (11.93, 25.52)	14423.79 (9648.45, 20755.28)	6667.05 (4449.04, 9631.24)	7756.74 (5199.41, 11124.04)
Wallis and Futuna Islands	19.19 (5.14, 55.00)	20.59 (5.40, 59.58)	17.93 (4.91, 50.91)	1.90 (0.51, 5.46)	0.96 (0.25, 2.79)	0.94 (0.26, 2.67)
Western Sahara	7.31 (4.84, 10.66)	7.63 (5.07, 11.08)	6.91 (4.55, 10.13)	35.66 (23.63, 52.00)	20.82 (13.84, 30.23)	14.85 (9.79, 21.77)
Yemen	9.34 (6.12, 13.85)	8.96 (5.87, 13.31)	9.73 (6.37, 14.4)	2587.36 (1694.18, 3837.11)	1251.41 (818.77, 1858.38)	1335.95 (875.40, 1978.73)

Country or territory	Prevalence (%, 95% CI)			Case number (thousand, 95% CI)		
	Both	Male	Female	Both	Male	Female
Zambia	9.08 (5.93, 13.54)	8.93 (5.82, 13.33)	9.24 (6.03, 13.74)	1334.67 (870.69, 1989.14)	646.44 (421.24, 965.11)	688.23 (449.44, 1024.03)
Zimbabwe	10.68 (7.01, 15.79)	10.06 (6.60, 14.91)	11.22 (7.38, 16.55)	1260.34 (827.50, 1862.28)	552.95 (362.42, 819.34)	707.39 (465.08, 1042.94)

Notes: CI, confidence interval.

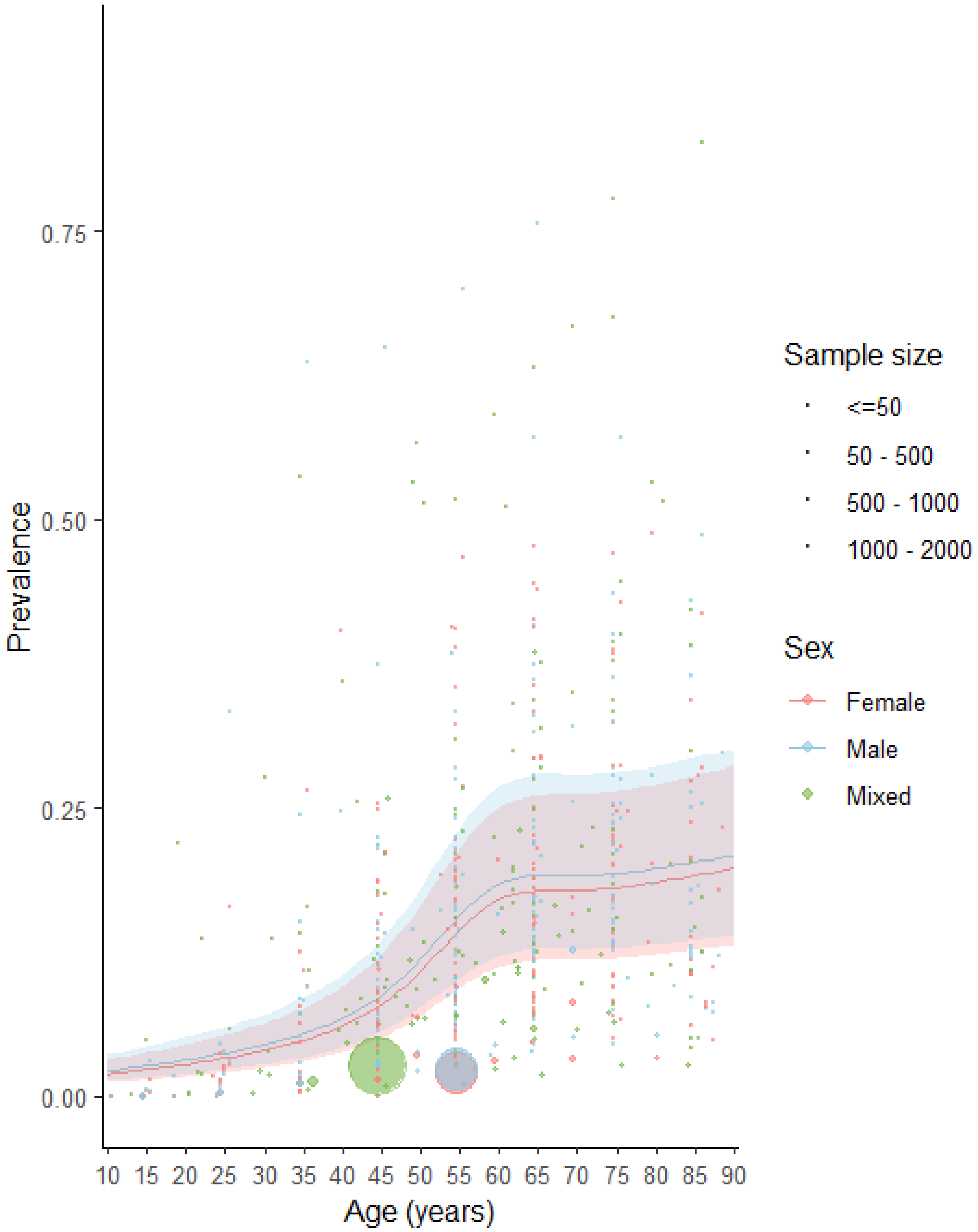

Figure S1. Multilevel mixed-effects meta-regression models for prevalence of pterygium.

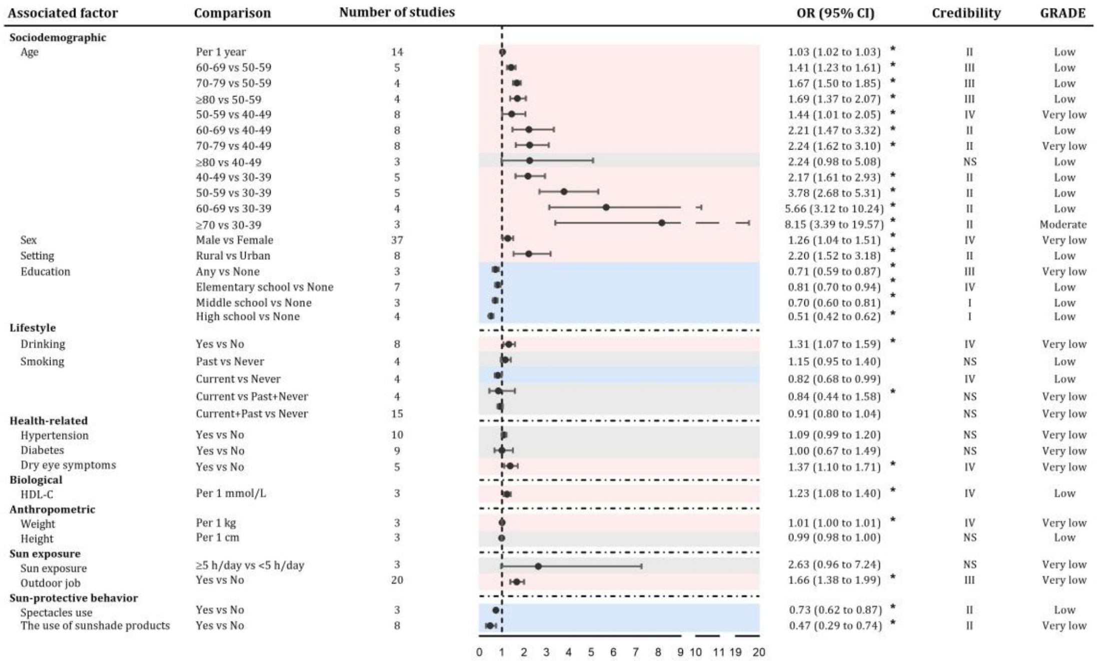

Figure S2. Summary of associated factors of pterygium.

Notes: HDL-C, high-density lipoprotein cholesterol; OR, odds ratio; CI, confidence interval; NS, not significant.

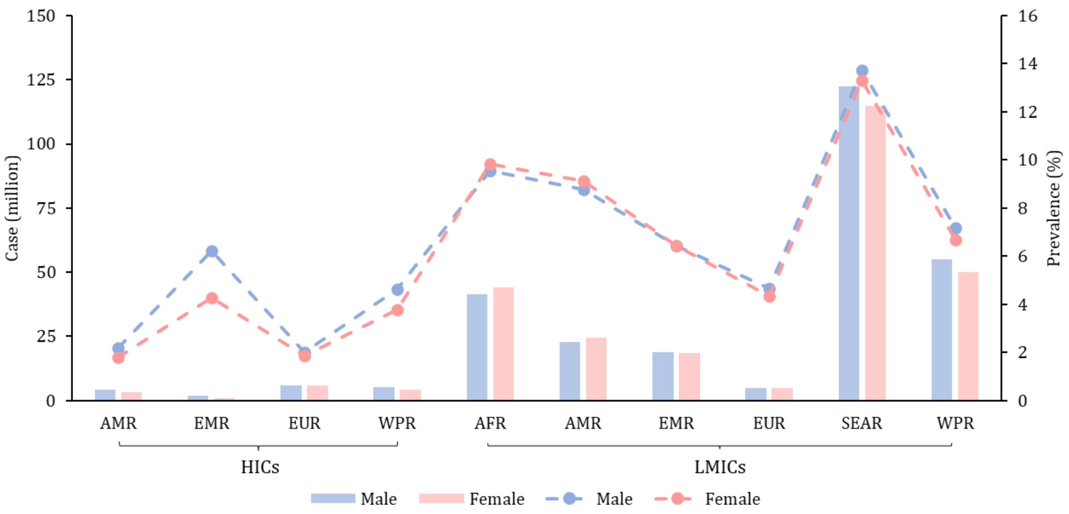

Figure S3. Regional sex-specific prevalence and case number of pterygium.

Notes: HICs, high-income countries; LMICs, low- and middle-income countries; WHO, World Health Organization; WB, World Bank; AFR, African Region; AMR, Region of the Americas; EMR, Eastern Mediterranean Region; EUR, European Region; SEAR, South-East Asia Region; WPR, Western Pacific Region.

Appendix 4. Full list of the included articles (n=103).

ID	Reference
S01	Dong YP, Wang YJ, Wang JM, et al. 董彦平, 王玉瑾, 王洁敏, 等. Investigation on the prevalence and influencing factors of pterygium in Gannan ethnic minority areas (甘南少数民族地区翼状胬肉患病率及影响因素调查研究)[J]. Anhui Medical and Pharmaceutical Journal (安徽医药). 2024;28(3).
S02	Adriano L, de Souza Persona EL, de Souza Persona IG, et al. Correlation between the presumed pterygium with dry eye and with systemic and ocular risk factors. Arq Bras Oftalmol. 2022;85(2):136-143.
S03	Zhang XY, Yan X, Guan RJ, Li L. 张晓英, 晏鑫, 关瑞娟, 李凌. Analysis of risk factors related to pterygium and establishment of prediction model in plateau area (高原地区翼状胬肉相关危险因素分析及预测模型的建立)[J]. International Eye Science (国际眼科杂志). 2022;22(7):1215-1219.
S04	Zang S, Chen Y, Guo H, et al. High HDL-C and high LDL-C are risk factors of pterygium in a population-based cross-sectional study in Southern China: the Dongguan Eye Study. BMJ Open. 2022;12(6).
S05	Tandon R, Vashist P, Gupta N, et al. The association of sun exposure, ultraviolet radiation effects and other risk factors for pterygium (the SURE RISK for pterygium study) in geographically diverse adult (≥40 years) rural populations of India -3rd report of the ICMR-EYE SEE study group. PLoS One. 2022;17(7):e270065.
S06	Padhy D, Majhi D, Marmamula S, et al. Tribal Odisha Eye Disease Study # 11 - Particularly vulnerable tribal group eye health program. Program protocol and validation. Indian J Ophthalmol. 2022;70(4):1376-1380.
S07	Ke HQ, Dong YJ, Liu H, et al. 柯红琴, 董宇婕, 刘海, 等. Prevalence and risk factors for pterygium in six rural regions of Yunnan Province (云南省 6 个地区农村人群翼状胬肉患病率及其影响因素的研究)[J]. Zhonghua Yan Ke Za Zhi (中华眼科杂志). 2022;58(10):769-777.
S08	Xiao Li, Guoqing Li, Jing Xu, et al. 李晓, 李国庆, 徐静, 等. The prevalence and risk factors of pterygium in Han and Kazak Ethnic groups in Tacheng, Xinjiang (新疆塔城地区汉族和哈萨克族人群翼状胬肉患病率及危险因素研究)[J]. Chin J Optom Ophthalmol Vis Sci (中华眼视光学与视觉科学杂志). 2021;23(12):903-909.
S09	Hatsusaka N, Yamamoto N, Miyashita H, et al. Association among pterygium, cataracts, and cumulative ocular ultraviolet exposure: A cross-sectional study in Han people in China and Taiwan. PLoS One. 2021;16(6):e253093.
S10	Zhang Yanling, Xing Lijuan, Jia Qi, et al. 张艳玲, 邢丽娟, 贾琪, 等. Epidemiological Investigation of Eye Diseases Among Adults Aged 50 Years or Above of Futian A District (某区 50 岁及以上人群眼病及影响因素的分析)[J]. Guide of China Medicine (中国医药指南). 2020;18(21):5-7.
S11	Liu Yanjun. 刘艳君. Analysis of Eye Health Screening of People Over 50 Years Old in Communities (社区 50 岁以上人群眼健康筛查的结果分析)[J]. China Health Standard Management (中国卫生标准管理). 2020;11(22):17-20.
S12	Wang Y, Shan G, Gan L, et al. Prevalence and associated factors for pterygium in Han and Mongolian adults: a cross-sectional study in inner Mongolian, China. BMC Ophthalmol. 2020;20(1):45.
S13	Pan ZX, Shan GL, Wang XJ, et al. 潘周娴, 单广良, 王雪娇, 等. Prevalence and risk factors of pterygium in Han and Yugur populations in Gansu Province, China (甘肃省汉族和裕固族的翼状胬肉患病率及相关危险因素研究)[J]. Zhonghua Yan Ke Za Zhi (中华眼科杂志). 2020;56(8):600-607.
S14	Fekadu SA, Assem AS, Adimassu NF. Prevalence of pterygium and its associated factors among adults aged 18 years and above in Gambella town, Southwest Ethiopia, May 2019. PLoS One. 2020;15(9):e237891.
S15	Alemayehu TK, Addis Y, Bizuneh ZY, et al. Prevalence and associated factors of pterygium among adults living in Kolla Diba Town, Northwest Ethiopia. Clin Ophthalmol. 2020;14:245-255.
S16	Fernandes AG, Salomão SR, Ferraz NN, et al. Pterygium in adults from the Brazilian Amazon Region: prevalence, visual status and refractive errors. Br J Ophthalmol. 2020;104(6):757-763.
S17	Wu Xiaolan, Yi Quanyong, Wu Yinan, et al. 吴晓兰, 易全勇, 邬一楠, 等. Epidemiological investigation of ophthalmopathy among adults aged 50 years or above in Ningbo (宁波地区 50 岁及以上人群眼病流行病学调查)[J]. Chinese Journal of General Practice (中华全科医学). 2019;17(3):491-495.
S18	Zhang J, Yang M, Zhu R, et al. 张俊芳, 杨梅, 朱蓉嵘, 等. Prevalence and associated factors for pterygium in rural people aged 50 years and above in Funing County, Jiangsu Province (江苏省阜宁县农村 50 岁及以上人群翼状胬肉流行病学调查及相关危险因素分析)[J]. Zhonghua Shiyan Yanke Zazhi/Chinese Journal of Experimental Ophthalmology (中华实验眼科杂志). 2019;37(3):212-217.

ID	Reference
S19	Pan Z, Cui J, Shan G, et al. Prevalence and risk factors for pterygium: a cross-sectional study in Han and Manchu ethnic populations in Hebei, China. BMJ Open. 2019;9(2):e25725.
S20	Lin YH, Sun CC, Yeung L, et al. Epidemiologic study of pterygium in Taiwan. Jpn J Ophthalmol. 2019;63(4):297-303.
S21	Bikbov MM, Zainullin RM, Kazakbaeva GM, et al. Pterygium Prevalence and Its Associations in a Russian Population: The Ural Eye and Medical Study. Am J Ophthalmol. 2019;205:27-34.
S22	Zhao Lizhen, Zhan Yan, Chen Shangru, et al. 赵丽珍, 詹研, 陈尚茹, 等. Epidemiological investigation of the incidence of pterygium in the physical examination population of Xinhui district (新会区体检人群翼状胬肉患病率的流行病学调查)[J]. China medicine and pharmacy (中国医药科学). 2017;7(14):174-176.
S23	Yang Chengyi. 杨成毅. The incidence and clinical characteristics of postoperative recurrence of pterygium in plateau area (高原地区翼状胬肉发病的相关危险因素及术后复发情况)[J]. Journal of Chinese Physician (中国医师杂志). 2017;19(09):1334-1337.
S24	Su Xiaolong, Zhao Fentu, Li Zhihui, et al. 苏小龙, 赵奋图, 李志辉, 等. Epidemiologic Survey and Analysis of Related Risk Factors for Ocular Pterygium in Shunde District of Foshan City (佛山市顺德地区眼翼状胬肉的流行病学调查及相关危险因素分析)[J]. China Health Standard Management (中国卫生标准管理). 2017;8(03):3-5.
S25	Sitompul R, Lestari YD, Siregar S, et al. The burden of ocular diseases in an underdeveloped village in Southwest Sumba, Eastern Indonesia, 2016. Med J Indones. 2017;26(4):277-285.
S26	Rim TH, Kang MJ, Choi M, et al. The incidence and prevalence of pterygium in South Korea: A 10-year population-based Korean cohort study. PLoS One. 2017;12(3):e171954.
S27	Hashemi H, Khabazkhoob M, Yekta A, et al. The prevalence and determinants of pterygium in rural areas. J Curr Ophthalmol. 2017;29(3):194-198.
S28	Cao XG, Li XX, Bao YZ. Relationship between pterygium and age-related cataract among rural populations living in two different latitude areas in China. Int J Clin Exp Med. 2017;10(2):3494-3501.
S29	Anbesse DH, Kassa T, Kefyalew B, et al. Prevalence and associated factors of pterygium among adults living in Gondar city, Northwest Ethiopia. PLoS One. 2017;12(3):e174450.
S30	Li Ming, Ru Lijuan, Liu Chuanmin, et al. 李明, 汝丽娟, 刘传敏, 等. Epidemiological investigation of pterygium among villagers in inland areas of Qingdao City* (青岛市内陆地区村民翼状胬肉的流行病学调查)[J]. World Latest Medicine Information (Electronic Version) (世界最新医学信息文摘). 2016;16(22):174-175.
S31	Li Mengqi, Li Xuan, Zhang Xiaoping, et al. 李梦琪, 李璇, 张晓萍, 等. Epidemiological survey on pterygium among the middle-aged and the elderly, Qingdao city (青岛市中老年人群翼状胬肉的流行病学调查)[J]. Prev Med Trib (预防医学论坛). 2016;22(09):667-669.
S32	Gan Linyang. 干霖洋. Prevalence and Risk Factors among Han and Titetan Population in Qinghai Province (青海省汉藏族翼状胬肉患病率及危险因素分析)[D]. Peking Union Medical College (北京协和医学院); 2016.
S33	Bi Yun, Wang Yonghong, Wu Mingyu. 毕云, 王永红, 吴鸣宇. A study of pterygium disease among healthy aged people in a hospital* (某院健康体检中老年人群翼状胬肉患病状况研究)[J]. Lab Med Clin (检验医学与临床). 2016;13(23):3403-3405.
S34	Zhong H, Chen Q, Li J, et al. Ethnic Variations in Pterygium in a Rural Population in Southwestern China: The Yunnan Minority Eye Studies. Ophthalmic Epidemiol. 2016;23(2):116-121.
S35	Wang JW, Zhang Y, Chen XD, et al. 王钧蔚, 张毅, 陈雪冬, 等. Epidemiologic survey of pterygium in the middle-aged and the senile in defined rural area of Heilongjiang Province (黑龙江省农村限定地区中老年人群翼状胬肉流行病学调查)[J]. International Eye Science (国际眼科杂志). 2016;16(5):930-933.
S36	Lin AD, Miles K, Brinks MV. Prevalence of Pterygia in Hawaii: Examining Cumulative Surfing Hours as a Risk Factor. Ophthalmic Epidemiol. 2016;23(4):264-268.
S37	Chen T, Ding L, Shan G, et al. Prevalence and racial differences in pterygium: a cross-sectional study in Han and Uygur adults in Xinjiang, China. Invest Ophthalmol Vis Sci. 2015;56(2):1109-1117.
S38	Ma Wenzun. 马文尊. Prevalence and associated risk factors of pterygium among adults aged 50 years and above in Juancheng County,Shandong province (鄄城县 50 岁及以上农村居民翼状胬肉流行现状及影响因素研究)[D]. Shandong: Shandong University (山东:山东大学). 2015.
S39	Ma Feng. 马峰. Epidemiological characteristics of pterygium in older population in Zhangjiachuan County,Gansu Provincce* (甘肃省张家川县中老年农民翼状胬肉的流行病学特点)[J]. Gansu Science and Technology (甘肃科技). 2015,31(13):117-118.
S40	Cui Xinhan, Xu Jianjiang, Lei Qihua, et al. 崔心瀚, 徐建江, 乐琦骅, 等. Prevalence and risk factors of pterygiam among people aged 50 or above in Jinshan District of shanghai (上海市金山区≥50 岁者翼状胬肉流行病学调查)[J]. Chin J Pract Ophthalmol (中国实用眼科杂志). 2015,33(4):436-440.

ID	Reference
S41	Shrestha S, Shrestha SM. Comparative study of prevalence of pterygium at high altitude and Kathmandu Valley. J Nepal Health Res Counc. 2014;12(28):187-190.
S42	Maharjan IM, Shreshth E, Gurung B, et al. Prevalence of and associated risk factors for pterygium in the high altitude communities of Upper Mustang, Nepal. Nepal J Ophthalmol. 2014;6(11):65-70.
S43	Li Z, Wu S, Mai J, et al. Prevalence of and risk factors for pterygia in a rural Northern Chinese population. Ophthalmic Epidemiol. 2014;21(6):378-383.
S44	Jiao W, Zhou C, Wang T, et al. Prevalence and risk factors for pterygium in rural older adults in Shandong Province of China: a cross-sectional study. Biomed Res Int. 2014;2014:658648.
S45	Jiang Zhiying, Tan Gang, Han Guiping, et al. 蒋志英, 谈刚, 韩贵屏, 等. Analysis of Epidemiological Characteristics of Pterygium in Middle-aged and Elderly People in Miao Autonomous County* (苗族自治县中老年人翼状胬肉的流行病学特点分析)[J]. Health Care Today (现代养生 B). 2014(4):286-286,287.
S46	Li Changjiang, Huang Mengyun, Li Qiuhui. 黎昌江, 黄梦运, 李秋慧. The Status of Eye Diseases and Influencing Factors of Non-Attendance to Medical Treatment among Residents Aged 60 and Above in Qionghai City* (琼海市≥60 岁居民眼疾病状况及未就诊影响因素)[J]. Chinese Journal of Gerontology (中国老年学杂志). 2014(17):4952-4953,4954.
S47	Luo Zhongling, Zhang Lixin, Zhu Guoping, et al. 罗中伶,张立新,朱国平,等. Prevalence survey on pterygium among people aged 40 and above in Hengli Town of Dongguan (东莞市横沥镇 40 岁及以上人群翼状胬肉患病率调查)[J]. Int Eye Sci (国际眼科杂志). 2014(7):1292-1294.
S48	Shen Furong. 申芙蓉. Epidemiological Investigation of Pterygium in People Over 50 Years Old in Sanhe Town, Jincheng City* (晋城市三河镇 50 岁以上人群翼状胬肉流行病学调查)[J]. Medical Information (医学信息). 2014(8):120-120.
S49	Tano T, Ono K, Hiratsuka Y, et al. Prevalence of pterygium in a population in Northern Japan: the Locomotive Syndrome and Health Outcome in Aizu Cohort Study. Acta Ophthalmol. 2013;91(3):e232-e236.
S50	Sun LP, Lv W, Liang YB, et al. The prevalence of and risk factors associated with pterygium in a rural adult Chinese population: the Handan Eye Study. Ophthalmic Epidemiol. 2013;20(3):148-154.
S51	Rim THT, Nam J, Kim EK, et al. Risk factors associated with pterygium and its subtypes in Korea: the Korean National Health and Nutrition Examination Survey 2008-2010. Cornea. 2013;32(7):962-970.
S52	Nangia V, Jonas JB, Nair D, et al. Prevalence and associated factors for pterygium in rural agrarian central India. The central India eye and medical study. PLoS One. 2013;8(12):e82439.
S53	Marmamula S, Khanna RC, Rao GN. Population-based assessment of prevalence and risk factors for pterygium in the South Indian state of Andhra Pradesh: the Andhra Pradesh Eye Disease Study. Invest Ophthalmol Vis Sci. 2013;54(8):5359-5366.
S54	Li Z, Cui H. Prevalence and associated factors for pterygium in a rural adult population (the Southern Harbin Eye Study). Cornea. 2013;32(6):806-809.
S55	Jiang Ying, Zhang Lijun. 蒋英, 张利军. Survey on Pterygium Disease in Jungereen Banner, Inner Mongolia Autonomous Region* (内蒙古自治区准格尔旗翼状胬肉患病情况调查)[J]. World Health Digest (中外健康文摘). 2013(20):420-420.
S56	Lu Hongyu, Ouyang Chunlian, Yi Zhiyun, et al. 卢红宇, 欧阳春莲, 易志云, 等. The epidemiological survey of the prevalence rate of pterygium in the southern Jiangxi rare earth mining area (赣南稀土矿区翼状胬肉流行病学调查及手术)[J]. Chin J Ocul Traum Occupat Eye Dis (中华眼外伤职业眼病杂志). 2013,35(3):180-182.
S57	Xie Mingjie, Li Youyi, Zhang Jun, et al. 谢明捷, 李友谊, 张俊, 等. Prevalence survey on peterygium among people aged 40 or above in Luzhou city of Sichuan province in 2011 (2011 年泸州市 40 岁及以上人群翼状胬肉患病率调查)[J]. Chin J Prac:t Ophthalmol (中国实用眼科杂志). 2013,31(9):1208-1211.
S58	Zhong H, Cha X, Wei T, et al. Prevalence of and risk factors for pterygium in rural adult chinese populations of the Bai nationality in Dali: the Yunnan Minority Eye Study. Invest Ophthalmol Vis Sci. 2012;53(10):6617-6621.
S59	Rezvan F, Hashemi H, Emamian MH, et al. The prevalence and determinants of pterygium and pinguecula in an urban population in Shahroud, Iran. Acta Med Iran. 2012;50(10):689-696.
S60	Ang M, Li X, Wong W, et al. Prevalence of and racial differences in pterygium: a multiethnic population study in Asians. Ophthalmology. 2012;119(8):1509-1515.
S61	Liu Caishuang. 刘彩双. Analysis of epidemiological investigation for pterygium prevalence rates in Chengde (承德市翼状胬肉患病率的流行病学调查)[J]. Modern Preventive Medicine (现代预防医学). 2012,39(19):4948-4949+4955.
S62	Liu Lili, Wang Wentian, Zhang Feng, et al. 刘利莉, 王文田, 张丰, 等. Epidemiological survey of pterygium in rural aged population in Chicheng County of Zhangjiakou City (张家口市赤诚县农村老年居民翼状胬肉的流行病学调查)[J]. Int Eye Sci (国际眼科杂志). 2013,13(01):153-155.

ID	Reference
S63	Yue Jianjun, Wu Lan, Zhang Jianjun. 岳建军, 伍岚, 张建军. A epidemiologic survey of the prevalence of pterygium in Dayawan,Guangdong province* (广东省大亚湾地区翼状胬肉患病率的流行病学调查)[J]. China Prac Med (中国实用医药). 2012,7(11):251.
S64	Sherwin JC, Hewitt AW, Kearns LS, et al. The association between pterygium and conjunctival ultraviolet autofluorescence: the Norfolk Island Eye Study. Acta Ophthalmol. 2013;91(4):363-370.
S65	Asokan R, Venkatasubbu RS, Velumuri L, et al. Prevalence and associated factors for pterygium and pinguecula in a South Indian population. Ophthalmic Physiol Opt. 2012;32(1):39-44.
S66	Yoon KC, Mun GH, Kim SD, et al. Prevalence of eye diseases in South Korea: data from the Korea National Health and Nutrition Examination Survey 2008-2009. Korean J Ophthalmol. 2011;25(6):421-433.
S67	Landers J, Henderson T, Craig J. Prevalence of pterygium in indigenous Australians within central Australia: the Central Australian Ocular Health Study. Clin Exp Ophthalmol. 2011;39(7):604-606.
S68	Tian Bingyu, Chen Li, He Yuan, et al. 田冰玉, 陈莉, 何媛, 等. Epidemiological Investigation of Pterygium in Rural Areas of Shaanxi Province* (陕西省农村翼状胬肉流行病学调查)[J]. Zhejiang Clinical Medical Journal (浙江临床医学). 2011,13(11):1272-1275.
S69	Chen Yaqiong, Yuan Yuan, Hu Yao. 陈雅琼,袁媛,胡耀. Epidemiologic survey of the prevalence of pterygium in the urban and rural areas of Wuhan (武汉城区及周边农村翼状胬肉患病率的流行病学调查)[J]. International Eye Science (国际眼科杂志). 2011,11(2):301-302.
S70	Chen Yongqin, Liu Xuewen, Chen Lijun, et al. 陈永勤, 刘学文, 陈立军, 等. A survey of the prevalence of pterygium in Bayinguoleng* (巴音郭楞蒙古自治州 5 县市翼状胬肉患病率的调查)[J]. Chinese Journal of Chinese Ophthalmology (中国中医眼科杂志). 2001,11(4):232-233.
S71	Liu Qingxia, Li Huiping, Du Wenzhang, et al. 刘青霞, 李慧平, 杜文章, 等. Epidemiological investigation of the prevalence rate of pterygium in Ningxia region (宁夏地区翼状胬肉患病率的流行病学调查)[J]. International Eye Science (国际眼科杂志). 2011,11(12):2200-2202.
S72	Lei Chuntao, Qiao Lifeng, Fan Yingchuan, et al. 雷春涛, 乔利峰, 樊映川, 等. Epidemiological survey of pterygium among people over 50 years old in An'yue County of Sichuan province (四川省安岳县 50 岁以上人群翼状胬肉流行病学调查)[J]. Practical Journal of Clinical Medicine (实用医院临床杂志). 2011,8(3):110-111.
S73	Viso E, Gude F, Rodríguez-Ares MT. Prevalence of pinguecula and pterygium in a general population in Spain. Eye (Lond). 2011;25(3):350-357.
S74	Liang QF, Xu L, Jin XY, et al. Epidemiology of pterygium in aged rural population of Beijing, China. Chin Med J (Engl). 2010;123(13):1699-1701.
S75	Feng Wangqiang, Su xiaoxia, Wang Aijun, et al. 冯旺强,苏小夏,王爱君,等. Analysis of Pterygium Disease Among Elderly Residents in Dongtou County* (洞头县老年居民翼状胬肉患病情况分析)[J]. China Modern Doctor (中国现代医生). 2010,48(33):103-104.
S76	Cajucom-Uy H, Tong L, Wong TY, et al. The prevalence of and risk factors for pterygium in an urban Malay population: the Singapore Malay Eye Study (SiMES). Br J Ophthalmol. 2010;94(8):977-981.
S77	West S, Muñoz B. Prevalence of pterygium in Latinos: Proyecto VER. Br J Ophthalmol. 2009;93(10):1287-1290.
S78	Shiroma H, Higa A, Sawaguchi S, et al. Prevalence and risk factors of pterygium in a southwestern island of Japan: the Kumejima Study. Am J Ophthalmol. 2009;148(5):766-771.
S79	Lu J, Wang Z, Lu P, et al. Pterygium in an aged Mongolian population: a population-based study in China. Eye (Lond). 2009;23(2):421-427.
S80	Yu Song, Zhou Minjun, Yu Xianglin, et al. 于松,周敏君,喻相林等. Survey on Pterygium Disease among Urban and Rural Residents in Xiangshan County* (象山县城乡居民翼状胬肉患病情况调查)[J]. Zhejiang Clinical Medical Journal (浙江预防医学). 2009,21(4):29-30. DOI:10.3969/j.issn.1007-0931.2009.04.015.
S81	Wu Xiaye. 吴侠业. Epidemiological Investigation of Pterygium in Ganyu County, Jiangsu Province* (江苏省赣榆县翼状胬肉流行病学调查)[J]. China Modern Doctor (中国现代医生). 2009,47(28):120-121.
S82	Gao Xining, Ge Jianjie, Bi Guofeng, et al. 高夕宁, 葛建杰, 毕国风, 等. Epidemiological characteristics of pterygium in Wendeng middle and old aged coastal residents (文登沿海乡镇中老年翼状胬肉的流行病学特点)[J]. Chin J Ophthalmol and Otorhinolaryngol (中国眼耳鼻喉科杂志). 2009,9(5):312-313.

ID	Reference
S83	He Quan, Zhu Shuangjun. 何全,朱双军. Epidemiological Investigation of Pterygium on Gaotang Island, Zhejiang Province* （浙江省高塘岛翼状胬肉的流行病学调查）[J]. Zhejiang Clinical Medical Journal (浙江临床医学). 2008,10(10):1383-1384.
S84	Qi Qige, Deng Shijing, Guo Zhenshan, et al. 其其格,邓世靖,郭振山,等. Epidemic Characteristics of Climatic Droplet Kerotopathy Combined with Pterygium (气候性滴状角膜变性合并翼状胬肉流行病学特点)[J]. Inner Mongolia Medical Journal (内蒙古医学杂志). 2008,40(4):459-462.
S85	Wu Xuan. 武烜. Survey on prevalence of pterygium in DaLiYaBoYi country of YuTian city (新疆于田县达里雅博依乡翼状胬肉患病率调查)[D]. Xinjiang: Xinjiang Medical University (新疆:新疆医科大学). 2008.
S86	Durkin SR, Abhary S, Newland HS, et al. The prevalence, severity and risk factors for pterygium in central Myanmar: the Meiktila Eye Study. Br J Ophthalmol. 2008;92(1):25-29.
S87	Ma K, Xu L, Jie Y, et al. Prevalence of and factors associated with pterygium in adult Chinese: the Beijing Eye Study. Cornea. 2007;26(10):1184-1186.
S88	Lu P, Chen X, Kang Y, et al. Pterygium in Tibetans: a population-based study in China. Clin Exp Ophthalmol. 2007;35(9):828-833.
S89	Li Jie. 李洁. Epidemiological survey of external ophthalmopathy and visual impairment among adults 40 years or older in Pingxiang County HeBei Province (河北省平乡县 40 岁及以上人群外眼病与视力损害的流行病学调查)[D]. Hebei: Hebei Medical University (河北:河北医科大学). 2007.
S90	Tan CS, Lim TH, Koh WP, et al. Epidemiology of pterygium on a tropical island in the Riau Archipelago. Eye (Lond). 2006;20(8):908-912.
S91	Paula JS, Thorn F, Cruz AA. Prevalence of pterygium and cataract in indigenous populations of the Brazilian Amazon rain forest. Eye (Lond). 2006;20(5):533-536.
S92	Cao Xuechuan. 曹雪川. The Epidemiological Survey and Risk Factor's Study on Waizhang Eye Diseases and Ocular Surface Diseases in Tibetan High-altitude Area (雪域高原外障眼病流行病学调查及其危险因素研究)[D]. Yunnan: Yunnan University of Traditional Chinese Medicine (云南:云南中医学院). 2006.
S93	Han Shuxia, Zheng Yuezhong. 韩曙霞,郑曰忠. A epidemiologic survey of pterygium in the middle-aged and the elderly in Dagang District,Tianjin City (天津市大港区中老年人翼状胬肉的流行病学调查)[J]. Chin J Pract Ophthalmol (中国实用眼科杂志). 2006,24(4):435-437.
S94	Wu K, He M, Xu J, et al. Pterygium in aged population in Doumen County, China. Yan Ke Xue Bao. 2002;18(3):181-184.
S95	Gazzard G, Saw SM, Farook M, et al. Pterygium in Indonesia: prevalence, severity and risk factors. Br J Ophthalmol. 2002;86(12):1341-1346.
S96	Chen Yongqin, Xu Kai, Zhang Li. 陈永勤, 徐凯, 张莉, 等. Epidemiologic characteristics of the prevalence of pterygium in older adults in Kuerle (库尔勒市中老年人翼状胬肉的流行病学特点)[J]. Chin J Pract Ophthalmol (中国实用眼科杂志). 2002,20(12):941-942.
S97	Wong TY, Foster PJ, Johnson GJ, et al. The prevalence and risk factors for pterygium in an adult Chinese population in Singapore: the Tanjong Pagar survey. Am J Ophthalmol. 2001;131(2):176-183.
S98	Luthra R, Nemesure BB, Wu SY, et al. Frequency and risk factors for pterygium in the Barbados Eye Study. Archives of ophthalmology (Chicago, Ill. : 1960). 2001;119(12):1827-1832.
S99	Liu H, Yang J, Zhong L. 刘汉生, 杨洁, 钟烈红, 等. Prevalence survey on pterygium in two counties of Hainan Province (海南省两县（市）翼状胬肉患病率的调查)[J]. Zhonghua Yan Ke Za Zhi (中华眼科杂志). 2001;37(1):21-23.
S100	McCarty CA, Fu CL, Taylor HR. Epidemiology of pterygium in Victoria, Australia. Br J Ophthalmol. 2000;84(3):289-292.
S101	Fang Yan, Zhao Changlong. 方严, 赵长龙. A Survey on Prevalence of Pterygium in Anhui Fengtai (安徽省凤台县翼状胬肉患病情况调查)[J]. Rec Adv Ophthalmol (眼科新进展). 2000,20(3):204-205.
S102	Panchapakesan J, Hourihan F, Mitchell P. Prevalence of pterygium and pinguecula: the Blue Mountains Eye Study. Aust N Z J Ophthalmol. 1998;26 Suppl 1:S2-S5.
S103	Newland HS, Woodward AJ, Taumoepeau LA, et al. Epidemiology of blindness and visual impairment in the kingdom of Tonga. The British journal of ophthalmology. 1994;78(5):344-348.

Notes: The Chinese publication list uses the official English names or abbreviations of the journals. English titles were obtained from the journals themselves or from literature databases (China National Knowledge Infrastructure, Wanfang, Chinese Biomedicine Literature Database, and China Science and Technology Journal Database). If an official

English translation of a journal name is not available, a Pinyin title is used. If an English translation of a title is not a vailable, we have translated the title, marked it with an asterisk (*), and highlighted it in green.
